# Genome-wide expression in human whole blood for diagnosis of latent tuberculosis infection: a multicohort research

**DOI:** 10.3389/fmicb.2025.1584360

**Published:** 2025-05-09

**Authors:** Fan Jiang, Yanhua Liu, Linsheng Li, Ruizi Ni, Yajing An, Yufeng Li, Lingxia Zhang, Wenping Gong

**Affiliations:** ^1^Institute of Tuberculosis Research, Senior Department of Tuberculosis, The Eighth Medical Center of PLA General Hospital, Beijing, China; ^2^Section of Health, No. 94804 Unit of the Chinese People’s Liberation Army, Shanghai, China; ^3^Resident Standardization Training Cadet Corps, Air Force Medical Center, Beijing, China; ^4^Graduate School, Hebei North University, Zhangjiakou, Hebei, China

**Keywords:** active tuberculosis, latent tuberculosis infection, diagnostic model, biomarkers, multicohort analysis

## Abstract

**Background:**

Tuberculosis (TB) remains a significant global health challenge, necessitating reliable biomarkers for differentiation between latent tuberculosis infection (LTBI) and active tuberculosis (ATB). This study aimed to identify blood-based biomarkers differentiating LTBI from ATB through multicohort analysis of public datasets.

**Methods:**

We systematically screened 18 datasets from the NIH Gene Expression Omnibus (GEO), ultimately including 11 cohorts comprising 2,758 patients across 8 countries/regions and 13 ethnicities. Cohorts were stratified into training (8 cohorts, *n* = 1,933) and validation sets (3 cohorts, *n* = 825) based on functional assignment.

**Results:**

Through Upset analysis, LASSO (Least Absolute Shrinkage and Selection Operator), SVM-RFE (Support Vector Machine Recursive Feature Elimination), and MCL (Markov Cluster Algorithm) clustering of protein–protein interaction networks, we identified S100A12 and S100A8 as optimal biomarkers. A Naive Bayes (NB) model incorporating these two markers demonstrated robust diagnostic performance: training set AUC: median = 0.8572 (inter-quartile range 0.8002, 0.8708), validation AUC = 0.5719 (0.51645, 0.7078), and subgroup AUC = 0.8635 (0.8212, 0.8946).

**Conclusion:**

Our multicohort analysis established an NB-based diagnostic model utilizing S100A12/S100A8, which maintains diagnostic accuracy across diverse geographic, ethnic, and clinical variables (including HIV co-infection), highlighting its potential for clinical translation in LTBI/ATB differentiation.

## 1 Introduction

Tuberculosis (TB) remains a leading global cause of morbidity and mortality, ranking as the top fatal infectious disease before the COVID-19 pandemic, surpassing even HIV/AIDS (Chen et al., 2024; An et al., 2025; Zhuang et al., 2024b). Despite being diagnosable, preventable, and treatable, persistent diagnostic challenges contribute to its high disease burden (Fortún and Navas, 2022). Current diagnostic approaches primarily rely on tuberculin skin tests (TST, Diaskintest, C-Tb, EC-test) and interferon-gamma release assays (IGRAs: T-SPOT.TB, QFT-GIT, QFT-Plus, LIASON QFT-Plus, LIOFeron TB/LTBI) (Gong and Wu, 2021; Li et al., 2024; Li et al., 2023). While these methods effectively distinguish active TB (ATB) from healthy controls (HCs), they lack precision in differentiating latent TB infection (LTBI) from ATB (Peng et al., 2024; Cheng et al., 2023; Wang et al., 2024; Jiang et al., 2023a; Jiang et al., 2023c; Jiang et al., 2023d).

To address this gap, the World Health Organization (WHO) has outlined target product profiles for novel diagnostics requiring: (1) non-sputum sampling (e.g., blood), (2) > 80% sensitivity in HIV co-infected patients, (3) > 66% sensitivity in pediatric culture-positive TB, and (4) operational simplicity [Global Programme on Tuberculosis and Lung Health (GTB), 2014]. This has spurred investigations into blood-based biomarkers using microarray technologies (Lu et al., 2019; Natarajan et al., 2022; Shao et al., 2021), complemented by emerging approaches in epigenetics (Esterhuyse et al., 2015), urinary metabolomics (Deng et al., 2021), Raman spectroscopy (Kaewseekhao et al., 2020), sputum proteomics/microbiomics (HaileMariam et al., 2021), NMR-based metabolomics (Izquierdo-Garcia et al., 2020), and machine learning-driven multi-marker profiling (Wang et al., 2024; Robison et al., 2019).

Nevertheless, critical limitations persist. Few studies have validated biomarkers in cohorts exceeding 2,000 cases, with scant evaluation in HIV co-infected or pediatric populations. Most proposed markers lack clinical trial validation (Jiang et al., 2023e; Jiang et al., 2023b), and while histological data mining shows promise, few studies leverage advanced computational methods (e.g., machine/deep learning) to enhance biomarker reliability.

To overcome these constraints, we conducted the largest GEO-based multicohort analysis to date (*n* = 2,758 across 8 countries/regions), integrating machine learning with single-cell validation. This study systematically explores LTBI/ATB diagnostic biomarkers through the rigorous reuse of NIH GEO datasets, aiming to advance translational TB research.

## 2 Methods

### 2.1 Cohort acquisition and curation

We systematically queried the NIH Gene Expression Omnibus (GEO) using: ((“tuberculosis” [MeSH Terms] OR tuberculosis [All Fields]) OR TB [All Fields]) AND “*Homo sapiens*” [porgn] AND “GDS” [Filter].

#### 2.1.1 Inclusion criteria

Studies involving whole or peripheral blood samples from patients with ATB (*n* = 11).

#### 2.1.2 Exclusion criteria

Studies focused on vaccines or cell cultures, two-sample arrays, non-blood samples, datasets excluding S100 genes (e.g., GSE144127), inconsistencies in data format, or unavailable matrices (*n* = 7).

The final cohorts included 2,758 patients from 8 countries/regions and 13 ethnicities (Table 1). LTBI and ATB classifications were based on the original study protocols, with household contacts categorized as LTBI (non-progressors) versus ATB (progressors). Given the heterogeneity of the 11 included cohorts and differences in sequencing platforms, we did not integrate all expression profiles but instead processed each cohort’s expression data individually. Feature selection and model development were also performed separately for each dataset.

**Table 1:** Basic information about the datasets.

Classification of data sets by purpose	Name of datasets	Availability of GEO2R analysis	Total number of patients	Number of ATB Patients	Number of LTBI Patients	Number of patients enrolled	Organization sources	Number of DEGs obtainable by GEO2R analysis
Discovery	GSE37250	Yes	537	195	167	362	Blood	113
	GSE39939	Yes	157	79	14	93	Blood	284
	GSE39940	Yes	334	111	54	165	Blood	264
	GSE101705	Yes	44	28	16	44	Blood	1,126
	GSE112104	Yes	51	29	21	50	Blood	3,389
	GSE19491	Yes	498	75	69	144	Blood	190
	GSE28623	Yes	108	46	25	71	Blood	821
	GSE40553	Yes	204	166	38	204	Blood	151
Validation	GSE94438	Yes	434	101	327	428	Blood	53
	GSE79362	Yes	355	110	245	355	Blood	31
	GSE84076	Yes	36	6	16	22	Blood	26
Excluded	GSE144127	Yes	628	301	13	314	Blood	20
	GSE83456	Yes	202	/	/	/	Blood	/
	GSE62147	Yes	52	/	/	/	Blood	/
	GSE41055	Yes	27	/	/	/	Blood	/
	GSE34608	Yes	24	/	/	/	Blood	/
	GSE84152	Yes	470	/	/	/	Blood	/
	GSE107995	No	414	/	/	/	Blood	/

### 2.2 Cohort stratification

Differential expression analysis (LTBI vs. ATB) identified genes with |logFC| ≥ 1 and adjusted *p* ≤ 0.05. Training set selection prioritized cohorts with consistent DEG numbers (8 cohorts, *n* = 1,933), while the validation set comprised outliers (3 cohorts, *n* = 825).

### 2.3 Training set analysis pipeline

Stable differential genes (SDGs) were defined as genes recurrently dysregulated in >50% of training cohorts, identified via Upset analysis. Feature selection was refined using two machine learning approaches: Least Absolute Shrinkage and Selection Operator (LASSO) regression and Support Vector Machine Recursive Feature Elimination (SVM-RFE). Protein–protein interaction (PPI) networks for SDGs were constructed using the STRING database, and functional modules were clustered via the Markov Cluster Algorithm (MCL)^1^. The diagnostic performance of gene clusters was evaluated through receiver operating characteristic (ROC) curves, with nested one-way ANOVA comparing sensitivity, specificity, positive/negative predictive values, and AUC metrics. Six machine learning models (Naïve Bayes, SVM, Elastic Net, LASSO, Logistic Regression, Ridge Regression) were iteratively tested to optimize diagnostic accuracy.

### 2.4 Validation set assessment

The validated diagnostic model was rigorously evaluated in three independent cohorts (*n* = 825) to ensure generalizability. ROC curves were generated to assess diagnostic performance metrics, including AUC, sensitivity, specificity, positive predictive value (PPV), and negative predictive value (NPV). The statistical significance of gene expression differences between LTBI and ATB groups was tested using the Mann–Whitney U test with a threshold of *p* < 0.05. Expression patterns were further validated against clinical metadata to ensure biological relevance.

### 2.5 Machine learning frameworks

#### 2.5.1 LASSO regression

The Least Absolute Shrinkage and Selection Operator (LASSO) regression was implemented using the glmnet R package. The algorithm applied L1 regularization to minimize the residual sum of squares, iteratively shrinking non-informative coefficients to zero. Ten-fold cross-validation was performed to optimize the penalty parameter (*λ*), and features retained at the minimum cross-validated error were selected for downstream analysis.

#### 2.5.2 SVM-RFE

Support Vector Machine Recursive Feature Elimination (SVM-RFE) utilized the Caret and kernlab packages. A radial basis function kernel was employed, and recursive feature elimination was conducted through five-fold cross-validation. Features were ranked by their contribution to the classification margin, with the least important features iteratively removed until an optimal subset was identified.

### 2.6 Network analysis and functional clustering

Protein–protein interaction (PPI) networks were constructed using the STRING database (version 11.5) with a combined interaction score threshold >0.4. The Markov Cluster Algorithm (MCL) was applied to partition the network into functional modules. Inflation parameters were automatically optimized to balance cluster granularity. FRIENDS analysis, implemented via custom scripts, calculated node centrality metrics (degree, betweenness, closeness) to identify hub genes within the network.

### 2.7 Statistical evaluation metrics

Nested one-way ANOVA was performed using GraphPad Prism 9.5.0 to assess hierarchical variance components across diagnostic metrics. The analysis tested interactions between sensitivity/specificity and PPV/NPV, as well as between cutoff values and AUC. Assumptions of normality (Shapiro–Wilk test) and homoscedasticity (Levene’s test) were verified prior to analysis. ROC curves were generated using the ROCR and pROC packages, with optimal cutoff values determined by maximizing Youden’s index (J = sensitivity + specificity − 1).

### 2.8 External validation resources

#### 2.8.1 CIBERSORT immune profiling

The CIBERSORT algorithm^2^ was executed with the LM22 leukocyte gene signature matrix. Bulk RNA-seq data were normalized using quantile normalization, and 1,000 permutations were performed to estimate immune cell proportions. Results were filtered for *p* < 0.05 to ensure confidence in deconvolution accuracy.

#### 2.8.2 Single-cell validation

The Broad Institute’s Single Cell Portal^3^ was queried for tuberculosis-related single-cell RNA-seq datasets. Gene expression patterns were visualized across cell types using embedded tools, with specificity confirmed by comparing expression levels in myeloid cells (monocytes, macrophages) versus lymphoid populations.

#### 2.8.3 GenDoma pathway analysis

GenDoma^4^ was accessed to map candidate biomarkers to disease pathways, regulatory networks (miRNA-gene, lncRNA-gene), and functional annotations. Enrichment analysis utilized Fisher’s exact test with Benjamini-Hochberg correction for multiple comparisons (q < 0.05).

### 2.9 Computational tools and workflow

Raw microarray data were preprocessed using GEOquery for dataset retrieval and limma for background correction and quantile normalization. Probe-to-gene annotation was performed with hgu133plus2.db for Affymetrix platforms. Network visualizations were generated using Cytoscape (v3.9.1) for PPI networks and ggplot2 for ROC curves. All code and reproducibility workflows are archived in Supplementary material 1.

## 3 Result

### 3.1 Dataset screening and stratification

**Figure 1:**
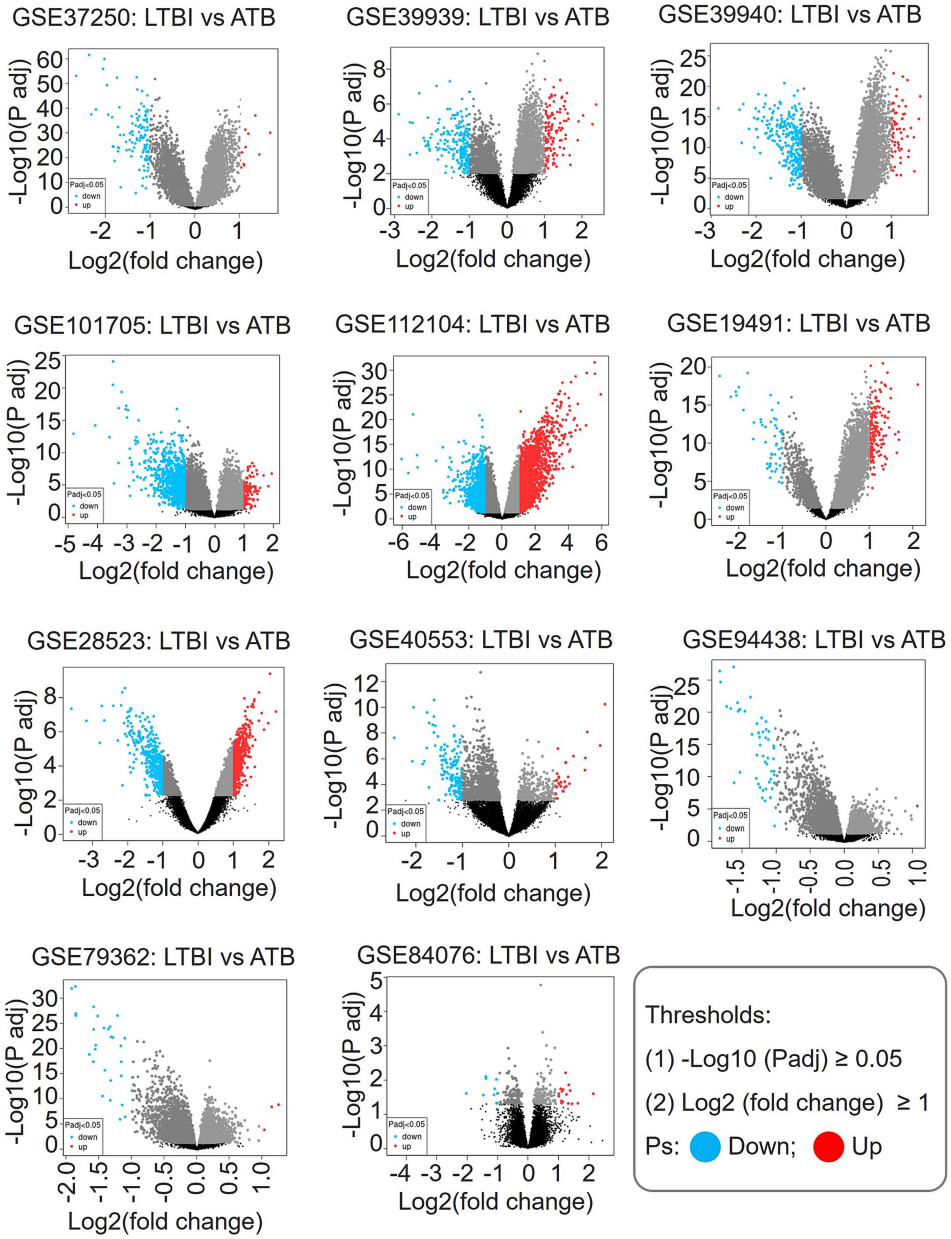
Differential gene volcano plot. The horizontal coordinate is the Log2FC value, the vertical coordinate is −Log10 (Padj), and genes with Padj < 0.05 are marked in blue for negative Log2FC values, in red for positive Log2FC values, and in black for genes with Padj > 0.05.

Eleven GEO datasets were analyzed, with eight assigned to the training set (GSE37250, GSE39939, GSE39940, GSE101705, GSE112104, GSE19491, GSE28623, GSE40553) and three to the validation set (GSE94438, GSE79362, GSE84076). Seven datasets were excluded due to non-blood samples or technical limitations (Table 1). Differential expression analysis (adjusted *p* ≤ 0.05, |logFC| ≥ 1) revealed substantial variability in DEG counts across cohorts, ranging from 26 (GSE84076) to 3,389 (GSE112104). Volcano plots and tabulated results (Figure 1; Table 1) highlight this heterogeneity, with GSE101705 and GSE112104 exhibiting the highest DEG counts (1,126 and 3,389, respectively).

### 3.2 Identification of stable differential genes (SDGs)

**Figure 2:**
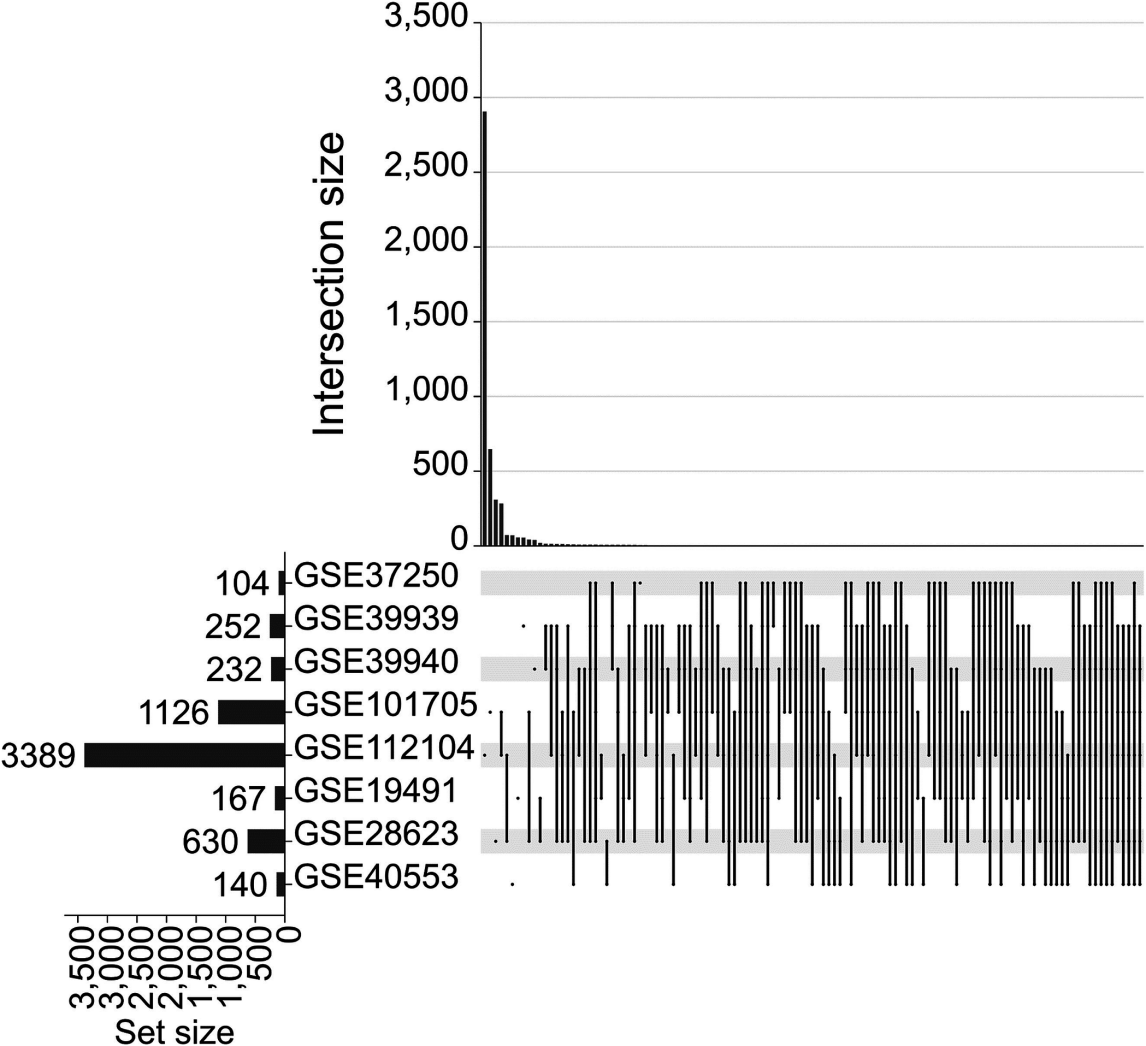
Upset graph for stable differential gene (SDG) screening. The set size indicates the number of all genes contained below this dataset. Dots indicate whether the interactable set is in a particular dataset. Dots connected by short lines indicate the presence of intersections in certain datasets. The number of genes that can be intersected together corresponds to the intersection size above.

**Figure 3:**
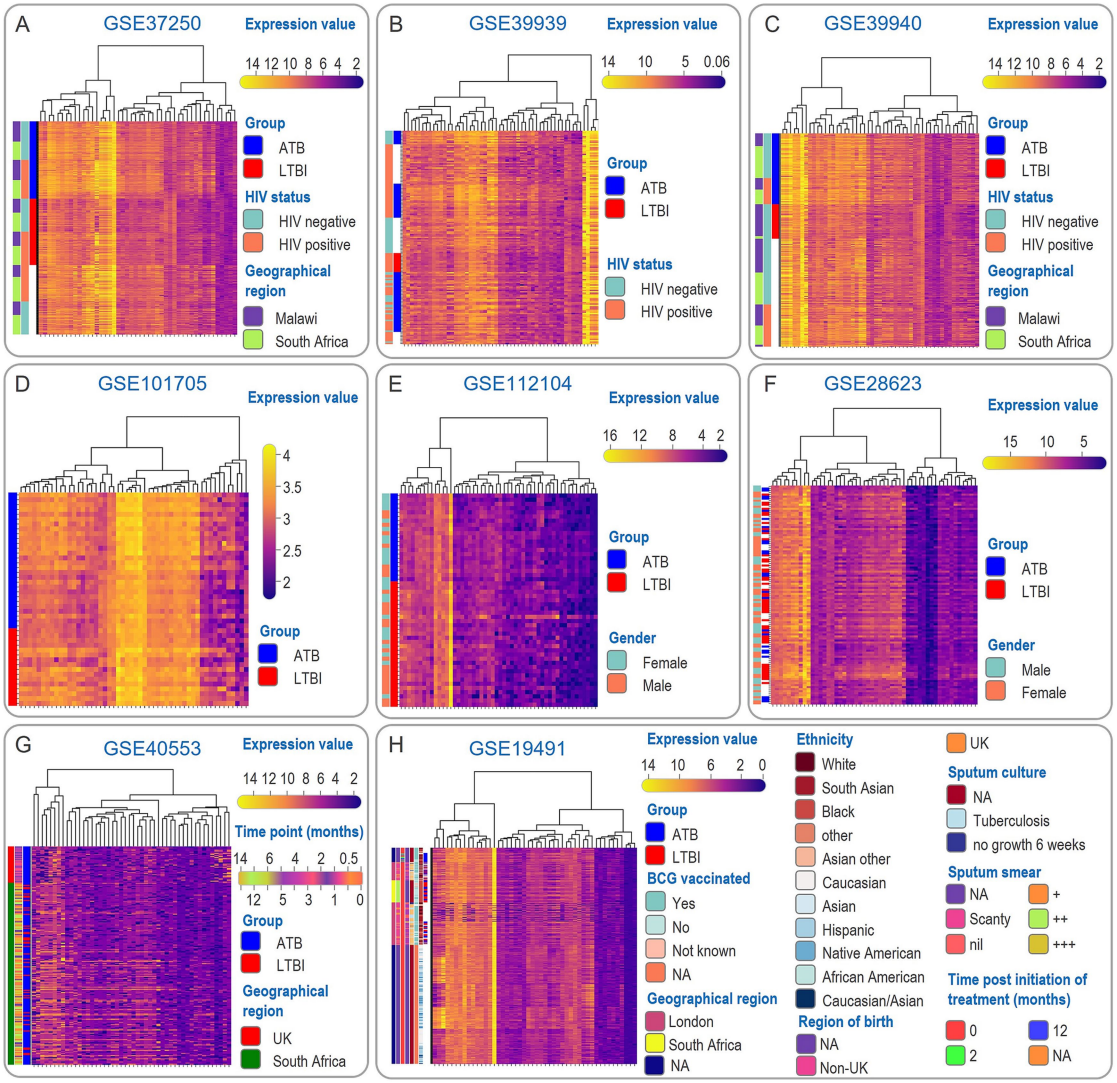
Heat map of SDG expression and clinical information. The SDGs obtained from Upset analysis were displayed as heatmaps of gene expression levels based on the clinical information in the respective datasets GSE37250 **(A)**, GSE39939 **(B)**, GSE39940 **(C)**, GSE101705 **(D)**, GSE112104 **(E)**, GSE28623 **(F)**, GSE40553 **(G)**, GSE19491 **(H)** and the clinical information corresponded to them to facilitate the visualization of the basic situation of the whole cohort.

Upset analysis of DEGs across eight training cohorts identified 55 SDGs recurrently intersected in >50% of datasets (Figure 2). These included immune-related genes (e.g., S100A12, S100A8, GBP5), inflammatory mediators (CXCR5, ELANE), and metabolic regulators (CYP1B1, MGST1). Hierarchical clustering of expression profiles (Figure 3) demonstrated consistent upregulation of S100A12 and S100A8 in ATB versus LTBI across training cohorts.

### 3.3 Machine learning-driven feature refinement and functional module discovery via PPI and MCL clustering

**Figure 4:**
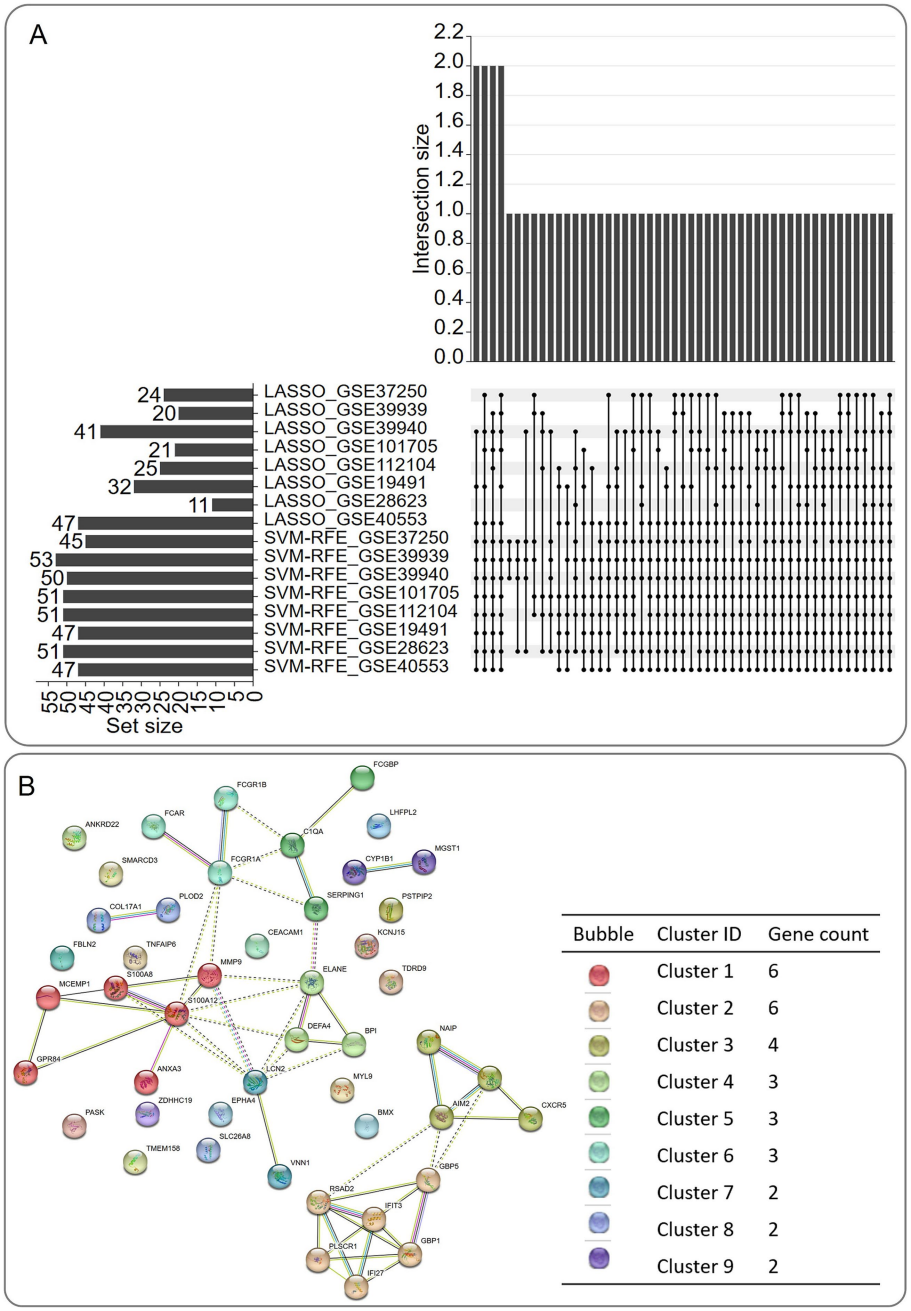
Screening of machine learning algorithm results and schematic diagram of protein–protein interaction (PPI) and MCL clustering algorithm results. **(A)** Upset diagram of machine learning algorithm results. **(B)** Schematic diagram of PPI and MCL clustering algorithm results for the corresponding proteins of genes after machine learning screening.

LASSO regression and SVM-RFE reduced the 55 SDGs to 47 high-confidence candidates (Figure 4A). In the results of PPI analysis, the interaction network maps between the proteins corresponding to the 47 SDG are shown in Figure 4B; based on the MCL clustering algorithm (the inflation parameter was set to 3), 31 of the 47 proteins were clustered into 9 classes (Figure 4B). Cluster 1 consisted of 6 genes (ANXA3, GPR84, MCEMP1. MMP9, S100A12, S100A8), Cluster 2 consisted of 6 genes (GBP1, GBP5, IFI27, IFIT3, PLSCR1, RSAD2), Cluster 3 consisted of 4 genes (AIM2, CXCR5, NAIPNLRC4), Cluster 4 consisted of 3 genes (BPI, DEFA4, ELANE), Cluster 5 consisted of 3 genes (C1QA, FCGBPSERPING1), Cluster 6 consisted of 3 genes (FCARFCGR1A, FCGR1B), Cluster 7 consisted of 2 genes (LCN2, VNN1), Cluster 8 consisted of 2 genes (COL17A1.PLOD2) and Cluster 9 consisted of 2 genes (CYP1B1, MGST1).

**Figure 5:**
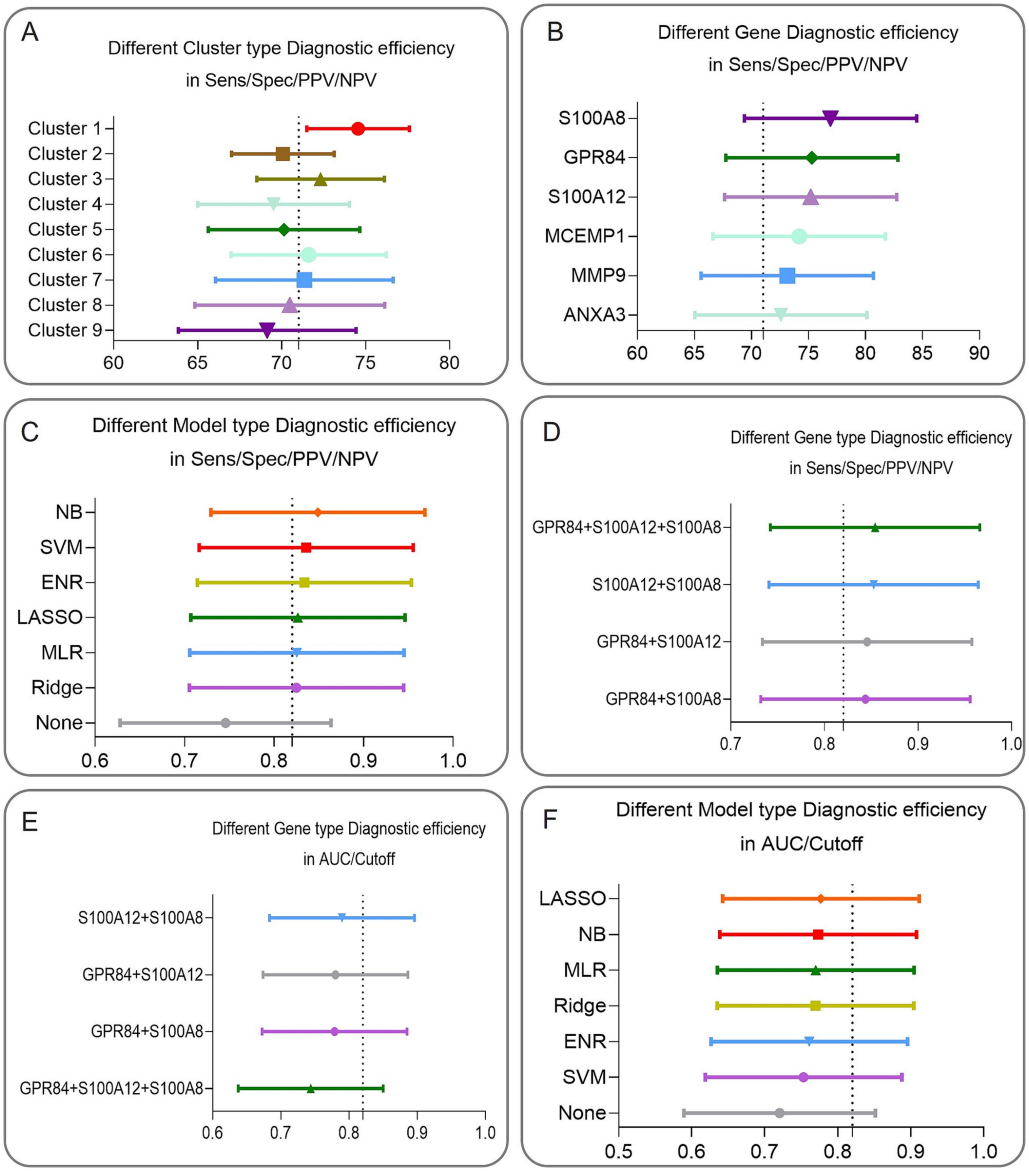
Schematic diagram of the results of nested one-way ANOVA analysis for clusters, genes, models, and gene combination types with better diagnostic efficacy. **(A)** The mean values of the nine clusters in the nested one-way ANOVA analysis under the first diagnostic efficacy perspective (consisting of Sens/Spec/PPV/NPV) are (Cluster 1 = 74.5505, Cluster 2 = 70.0766, Cluster 3 = 72.3194, Cluster 4 = 69.5216. Cluster 5 = 70.1375, Cluster 6 = 71.606, Cluster 7 = 71.3516, Cluster 8 = 70.4857, Cluster 9 = 69.1406), Cluster 1 showed the best diagnostic performance. **(B)** Nested one-way ANOVA analysis under the first perspective were (ANXA3 = 72.5781, GPR84 = 75.2875, MCEMP1 = 74.1844, MMP9 = 73.1344, S100A12 = 75.1875, S100A8 = 76.9313), where only GPR84, S100A12, and S100A8 had diagnostic efficacy greater than the overall diagnostic efficacy of 74.5505, and thus GPR84, S100A12, and S100A8 were considered as the three genes with better diagnostic efficacy. **(C)** In the first perspective, the ranking of NB was at the top 1. **(D)** In the second perspective (consisting of AUC/Cutoff value), the ranking of NB was at the top 2. **(E)** Four portfolio types (GPR84 + S100A12 + S100A8, S100A12 + S100A8, GPR84 + S100A8, GPR84 + S100A12) were evaluated, and two types (GPR84 + S100A12 + S100A8 and S100A12 + S100A8) showed superior diagnostic efficacy in the first angle. **(F)** In the second perspective, type (S100A12 + S100A8) showed better diagnostic efficacy than all the other three types.

The Sens/Spec/PPV/NPV of each of the nine clusters were obtained, and cluster 1 was found to have the highest diagnostic efficacy after descending the order of the clusters (Figure 5A). Cluster 1 contains six genes, and three genes, GPR84, S100A12, and S100A8, had higher Sens/Spec/PPV/NPV than three genes, ANXA3, MCEMP1, and MMP9, and therefore three genes, GPR84, S100A12, and S100A8, were included in the subsequent analysis (Figure 5B). The Sens/Spec/PPV/NPV of the six models constructed by three-gene signatures with a single biomarker, respectively, are NB (Average = 0.8490) > SVM (Average = 0.8360) > ENR (Average = 0.8338) > LASSO (Average = 0.8266) > MLR (Average = 0.8255) > Ridge (Average = 0.8251) > None (Average = 0.7458), indicating that the constructed model can significantly improve the prediction efficacy (Figure 5C). To further optimize the gene signature from the perspective of diagnostic efficacy, four combinations of Sens/Spec/PPV/NP for three genes were compared, GPR84 + S100A12 + S100A8 (Average = 0.8541) > S100A12 + S100A8 (Average = 0.8525) > GPR84 + S100A12 (Average = 0.8456) > GPR84 + S100A8 (Average = 0.8438, Figure 5D). For AUC/Cutoff, S100A12 + S100A (Average = 0.7897) > GPR84 + S100A12 (Average = 0.7788) > GPR84 + S100A8 (Average = 0.7801) > GPR84 + S100A12 + S100A8 (Average = 0.7440, Figure 5E). Because the 2 gene signature of S100A12 + S100A8 has been consistently ranked in the top two in terms of diagnostic efficacy, S100A12 + S100A8 is considered the optimal combination. The Sens/Spec/PPV/NPV of the six models constructed based on 2 gene signatures with gene signature were, respectively, LASSO (Average = 0.7769) > NB (Average = 0.7732) > MLR (Average = 0.7699) > Ridge (Average = 0.7696) > ENR (Average = 0.7611) > SVM (Average = 0.7532) > None (Average = 0.7205, Figure 5F). NB is regarded as the best model construction method because it is firmly in the top two in both the 3-gene signature and 2-gene signature model construction.

### 3.4 Biomarker validation across cohorts

**Figure 6:**
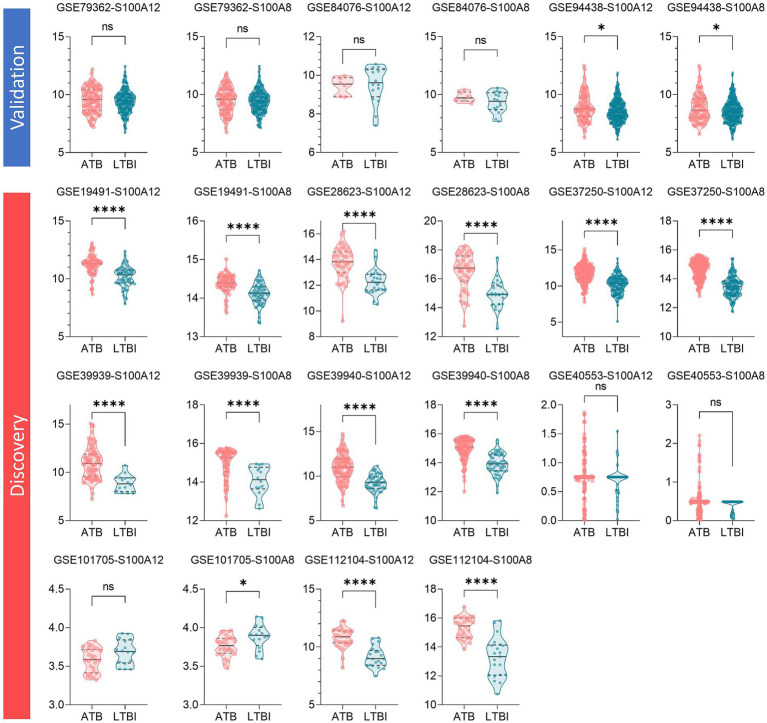
Schematic visualization of biomarker’s expression significance test results in each dataset. In the three validation sets, only GSE94438 showed significant differences in the expression of the two genes, S100A12 and S100A8, among the different populations; on the contrary, in the eight training sets, except for GSE40553 and GSE101705, the other six training sets showed significant up-regulation of the expression of the two genes, S100A12 and S100A8, in the ATB population in comparison with the LTBI population.

Mann–Whitney tests confirmed significant upregulation of S100A12 and S100A8 in ATB versus LTBI across six training cohorts (Figure 6). Validation cohorts showed variable performance (Figure 6): GSE94438 exhibited significant differential expression (*p* < 0.05), while GSE79362 and GSE84076 lacked consistency, potentially reflecting cohort-specific confounders (e.g., HIV co-infection).

### 3.5 Subgroup-specific diagnostic performance

**Figure 7:**
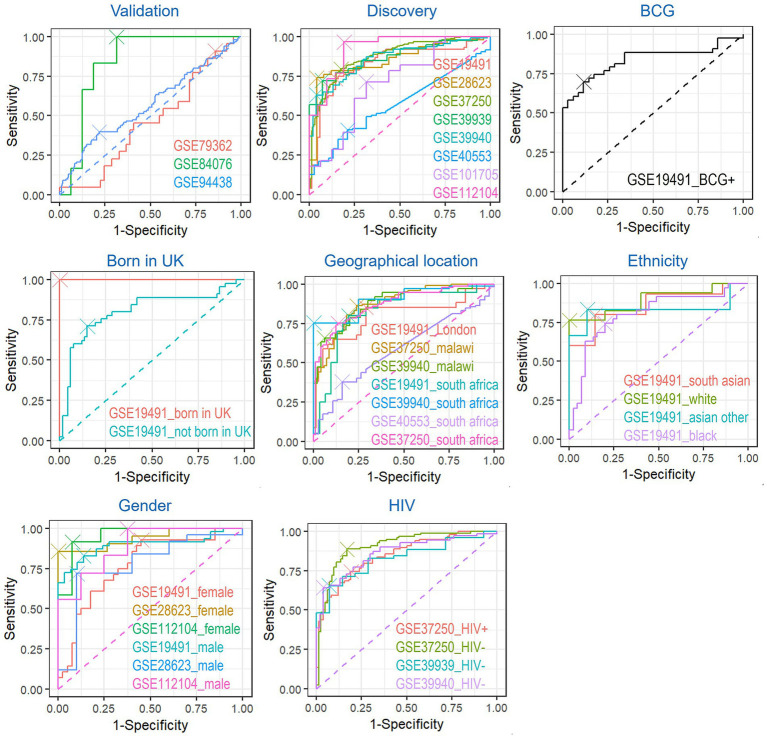
Visualization of the diagnostic performance of biomarkers after naive Bayes modeling in each dataset. The ROC curves for the 11 datasets (3 in validation and 8 in discovery) and eight subgroups were shown and visualized with the Cutoff point.

ROC analysis revealed variability across demographic and clinical subgroups (Figure 7; Table 2). The model achieved near-perfect discrimination (AUC = 1.0000) in UK-born individuals (GSE19491) and children in GSE112104. On the contrary, the 2-gene signature performed poorly in GSE79362 (AUC = 0.4610). Geographic, ethnic, and HIV status influenced accuracy: South Africa (GSE19491 = 0.8258, GSE39940 = 0.9041, GSE40553 = 0.5875, GSE37250 = 0.8730), Malawi (GSE37250 = 0.8732, GSE39940 = 0.8747), London (GSE19491 = 0.8042), Asian (GSE19491_South Asian = 0.8571, GSE19491_asian other = 0.8333) and Black (GSE19491 = 0.8044) cohorts showed robust prediction performance, while HIV-negative individuals (GSE37250 = 0.907, GSE39939 = 0.8297, GSE39940 = 0.8635) outperformed HIV co-infected patients (GSE37250 = 0.8490).

**Table 2:** Evaluation of the diagnostic efficacy of a simple Bayesian model with a two-gene signature.

No.	GSE name	Tag	Classification	AUC	Cutpoint	Sens	Spec	PPV	NPV
#1	GSE79362	Not used in subgroup analysis	Validation	0.4610	0.2885	0.9090	0.1430	0.3230	0.7780
#2	GSE84076	Not used in subgroup analysis		0.8438	0.1881	1.0000	0.6880	0.5450	1.0000
#3	GSE94438	Not used in subgroup analysis		0.5719	0.2324	0.3960	0.7830	0.3600	0.8080
25% Percentile				0.5165	0.2103	0.6525	0.4155	0.3415	0.7930
Median				0.5719	0.2324	0.9090	0.6880	0.3600	0.8080
75% Percentile				0.7079	0.2213	0.8263	0.7355	0.4525	0.9040
#4	GSE19491	BCG	Training	0.8311	0.6761	0.7330	0.8550	0.8460	0.7470
#5	GSE28623	Gender		0.8530	0.8398	0.7390	0.9600	0.9710	0.6670
#6	GSE37250	Geographical location/HIV		0.8764	0.5390	0.7950	0.8260	0.8420	0.7750
#7	GSE39939	Geographical location/HIV		0.8689	0.9220	0.7220	0.9290	0.9830	0.3710
#8	GSE39940	Geographical location/HIV		0.8614	0.8822	0.6310	0.9630	0.9720	0.5590
#9	GSE40553	Geographical location/HIV		0.5721	0.8197	0.4100	0.7890	0.8950	0.2340
#10	GSE101705	Not used in subgroup analysis		0.7076	0.6433	0.7140	0.6880	0.8000	0.5790
#11	GSE112104	Gender		0.9254	0.2813	0.9670	0.8100	0.8790	0.9440
25% Percentile				0.8002	0.6172	0.6933	0.8048	0.8450	0.5120
Median				0.8572	0.7479	0.7275	0.8405	0.8870	0.6230
75% Percentile				0.8708	0.8504	0.7530	0.9368	0.9713	0.7540
#12	GSE19491_BCG+	BCG?	Subgroup	0.8359	0.7268	0.6980	0.8860	0.8820	0.7050
#13	GSE19491_born in UK	Born in UK?		1.0000	1.0000	1.0000	1.0000	1.0000	1.0000
#14	GSE19491_not born in UK	Born in UK?		0.8027	0.5296	0.7110	0.8510	0.7620	0.8140
#15	GSE19491_London	Geographical location?		0.8042	0.4051	0.8530	0.7110	0.7250	0.8440
#16	GSE37250_Malawi	Geographical location?		0.8732	0.5236	0.8630	0.7610	0.8380	0.7940
#17	GSE39940_Malawi	Geographical location?		0.8747	0.6992	0.6580	0.9400	0.8930	0.7830
#18	GSE19491_South africa	Geographical location?		0.8258	0.4040	0.8000	0.8060	0.7270	0.8620
#19	GSE39940_South africa	Geographical location?		0.9041	0.9818	0.7530	1.0000	1.0000	0.1820
#20	GSE40553_South africa	Geographical location?		0.5875	0.7765	0.3790	0.8420	0.8930	0.2810
#21	GSE37250_South africa	Geographical location?		0.8730	0.5401	0.7530	0.8650	0.8430	0.7830
#22	GSE19491_South asian	Ethnicity?		0.8571	0.8075	0.8000	0.8570	0.9230	0.6670
#23	GSE19491_white	Ethnicity?		0.8941	0.8784	0.7650	1.0000	1.0000	0.5560
#24	GSE19491_asian other	Ethnicity?		0.8333	0.3743	0.8330	0.9000	0.8330	0.9000
#25	GSE19491_black	Ethnicity?		0.8044	0.4797	0.7430	0.8000	0.7430	0.8000
#26	GSE19491_female	Gender?		0.7714	0.2392	0.9290	0.5500	0.5910	0.9170
#27	GSE28623_female	Gender?		0.9397	0.8784	0.8570	1.0000	1.0000	0.8330
#28	GSE112104_female	Gender?		0.9551	0.4425	0.9170	0.9230	0.9170	0.9230
#29	GSE19491_male	Gender?		0.8951	0.7826	0.8300	0.8620	0.9070	0.7580
#30	GSE28623_male	Gender?		0.7760	0.7995	0.7200	0.9000	0.9470	0.5620
#31	GSE112104_male	Gender?		0.8889	0.2940	1.0000	0.6250	0.8570	1.0000
#32	GSE37250_HIV+	HIV?		0.8490	0.5855	0.7450	0.8100	0.8200	0.7310
#33	GSE37250_HIV-	HIV?		0.9070	0.4695	0.8870	0.8310	0.8600	0.8620
#34	GSE39939_HIV-	HIV?		0.8297	0.8805	0.6540	0.9290	0.9710	0.4190
#35	GSE39940_HIV-	HIV?		0.8635	0.8314	0.6430	0.9630	0.9570	0.6750
#36	GSE112104_children	Children?		1.0000	1.0000	1.0000	1.0000	1.0000	1.0000
#37	GSE19491_adult	Children?		0.8166	0.6698	0.7300	0.8410	0.8440	0.7260
#38	GSE112104_adult	Children?		0.8873	0.3329	0.9410	0.6670	0.8000	0.8890
25% Percentile				0.8212	0.4560	0.7250	0.8080	0.8265	0.6900
Median				0.8635	0.6698	0.8000	0.8620	0.8820	0.7940
75% Percentile				0.8946	0.8195	0.8750	0.9345	0.9520	0.8755

### 3.6 Immune cell correlates of biomarkers and single-cell expression validation

**Figure 8:**
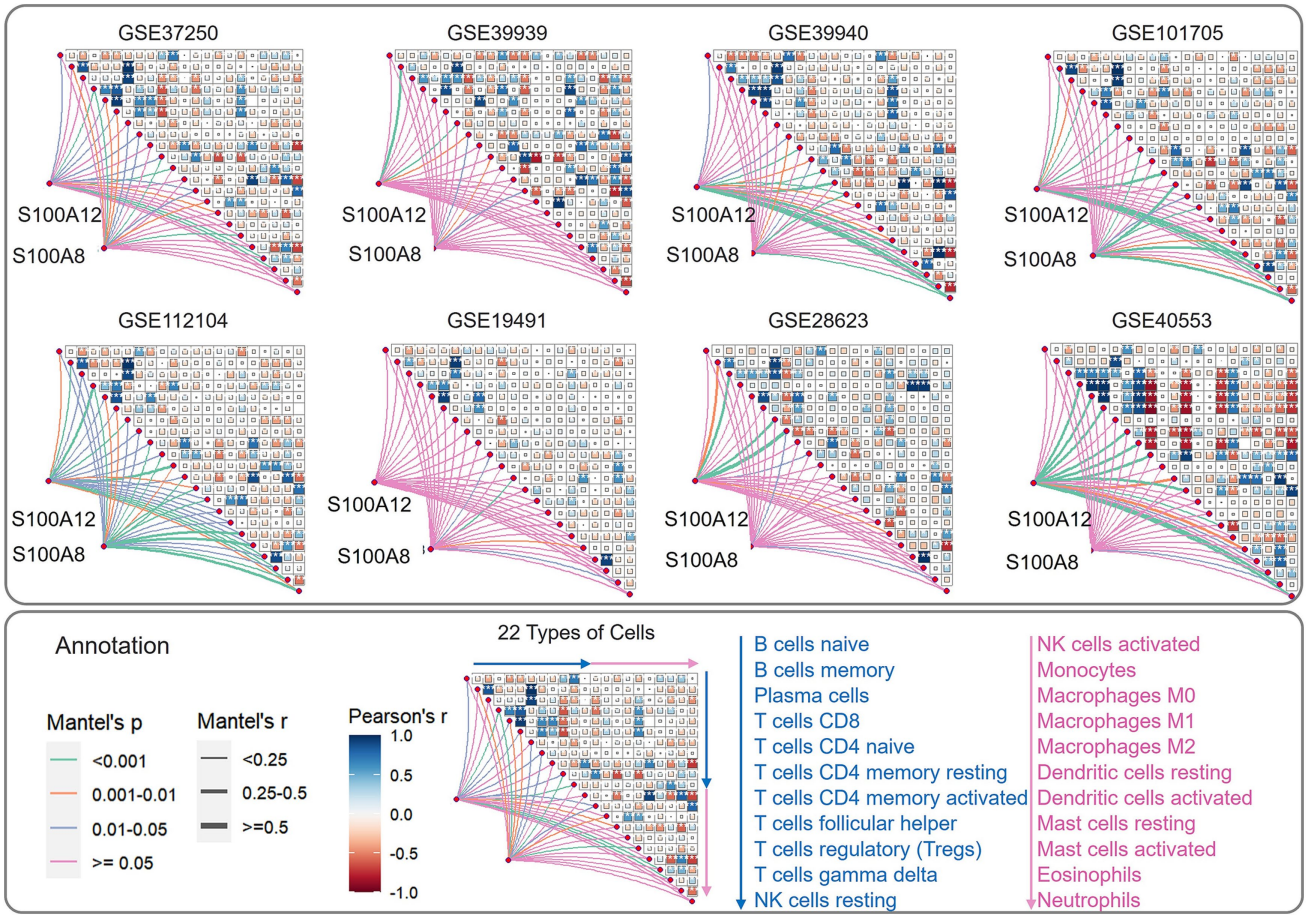
Schematic visualization of immune cell correlation based on immune infiltration analysis and mantel test for biomarker. The order of the 22 immune cells was 11 cells from top to bottom in the blue sequence, followed by 11 cells from top to bottom in the pink sequence. The order of the various immune cells in the correlation analysis was consistent with the direction of the arrows. The meaning of the heatmap in the triangular section was the heatmap analysis of the correlation of the results of immune cell infiltration in different data sets. The short lines connected to the heatmaps indicated the results of the analysis of the correlation between genes and immune cells.

CIBERSORT-based immune infiltration analysis was performed on all eight datasets, and S100A12 and S100A8 were screened against 64 immune cells with *p* < 0.05 in the Mantel test results, and a stable correlation between the three types of cells (CD4^+^ T cells, neutrophils, and NK cells) and 2 gene signature was observed after taking the intersection (Figure 8). The intersection of CD4^+^ T cells, neutrophils, and NK cells showed a stable correlation (Figure 8).

**Figure 9:**
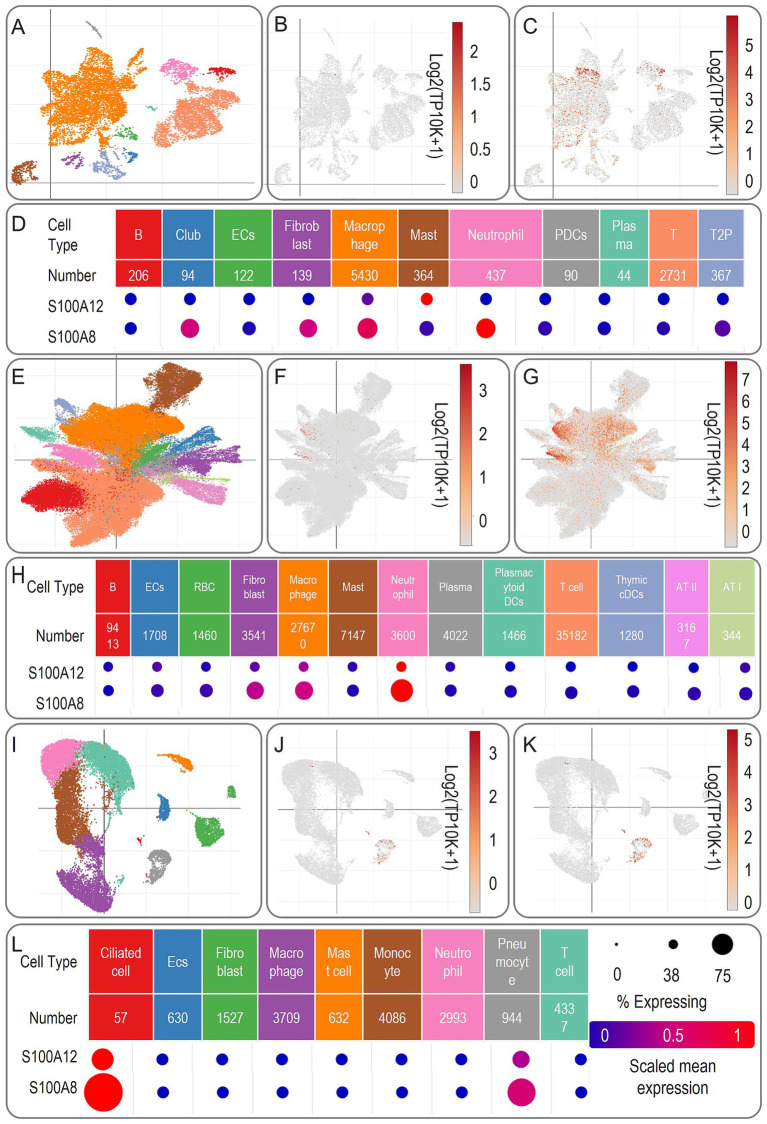
Schematic representation of biomarker expression in single-cell sequencing results. **(A,E,I)** Showed the planar projections of the cell numbers of single-cell sequencing results at 4 weeks, 10 weeks of tuberculosis infection, and tuberculosis and HIV co-infection. **(B,F,J)** Showed the heatmaps of the expression of S100A12 in different cells under the three conditions. **(C,G,K)** Showed the heatmaps of the expression of S100A8 in different cells under the three conditions. **(D,H,L)** Showed the matrix heatmaps of the expression of two genes, namely, S100A12 and S100A8, in different cells.

To verify in which cells the two genes S100A12 and S100A8 are highly expressed, we further validated the expression of the two genes using a single-cell dataset. First, 10,006 cells from 2 non-human primates at 6 weeks after infection with *Mycobacterium tuberculosis* (MTB)^5^ were used to observe the expression of S100A12 and S100A8 genes (Figures 9A–D). S100A12 was expressed at a high level in Mast cells, and S100A8 was expressed at a high level in Club cells (also known as bronchiolar exocrine cells), Fibroblast cells, Macrophage cells, and Neutrophil cells.

Next, 109,584 cells from 4 non-human primates at 10 weeks after infection with MTB^6^ were used to observe the expression of two genes, S100A12 and S100A8 (Figures 9E–H). S100A12 was expressed at high levels in Macrophage and Neutrophil cells, and S100A8 was expressed at high levels in Fibroblast cells, Macrophage cells, and Neutrophil cells.

Further, we used 18,915 cells from human lung tissue ACE2 + co-infected with *MTB* and HIV^7^ was performed to observe the expression of two genes, S100A12 and S100A8 (Figures 9I–L). S100A12 and S100A8 were expressed at high levels in Ciliated Cell cells and Pneumocyte cells.

### 3.7 Network enrichment and functional annotation

**Figure 10:**
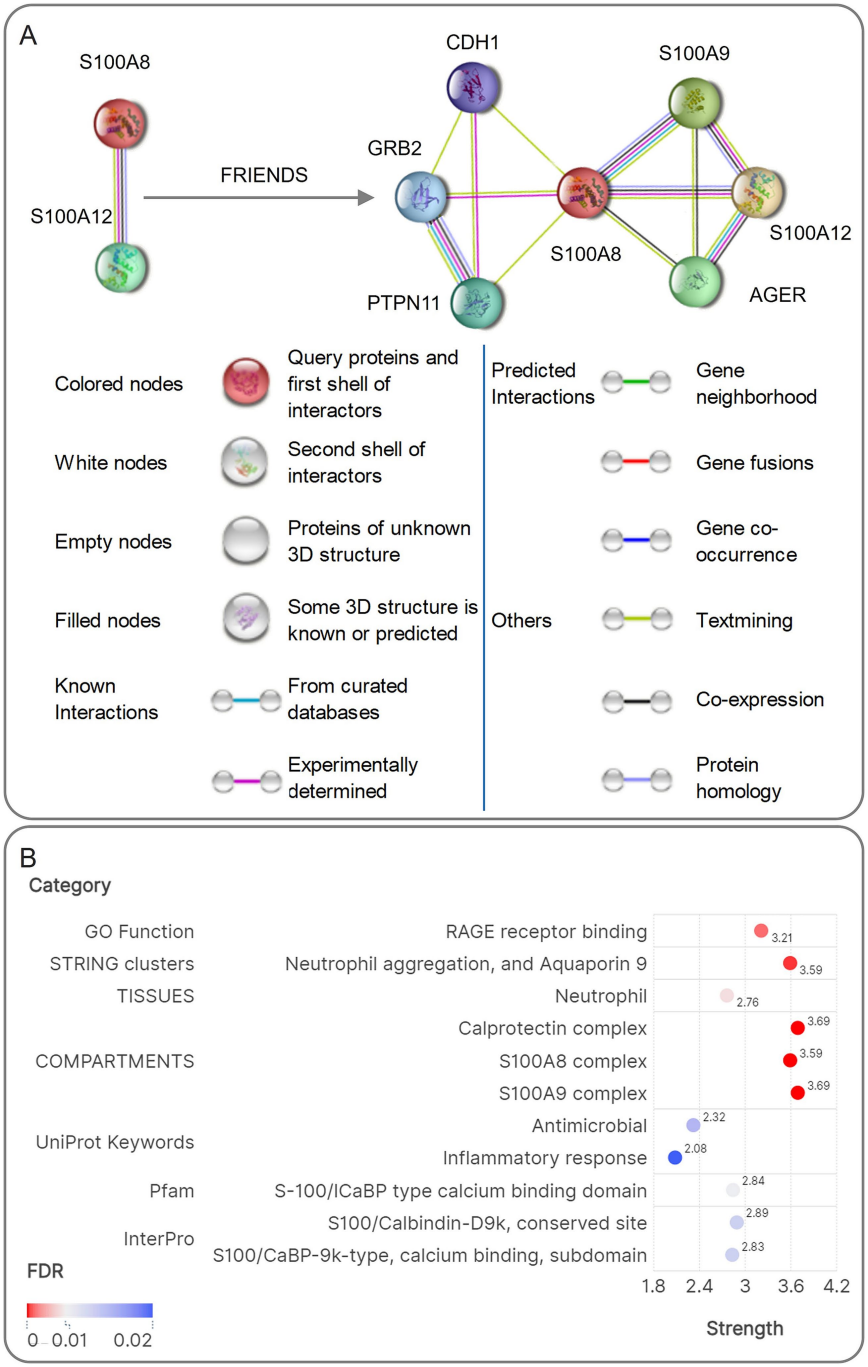
Visualization schematic of the results of PPI analysis, FRIENDS analysis, and enrichment analysis of PPI. **(A)** The PPI network diagram and the PPI of the FRIENDS analysis results for S100A12 and S100A8. **(B)** The heatmap visualization of the enrichment analysis results for the PPI network.

**Figure 11:**
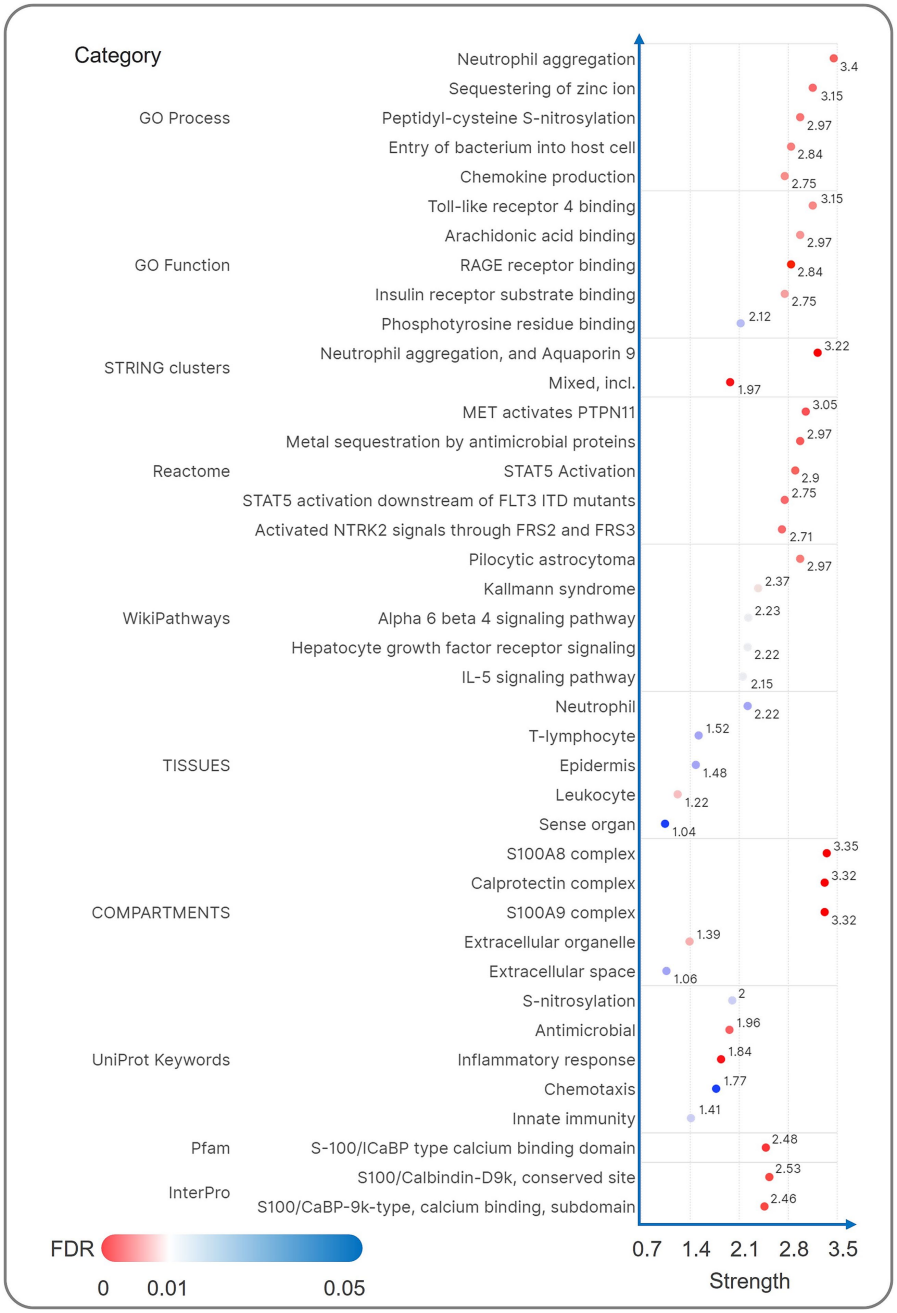
The enrichment analysis result of Schematic visualization of FRIENDS analysis. The FRIENDS analysis interaction network of S100A12 and S100A8 showed a strong association with neutrophil differentiation, Calprotein complex, and other functions in the enrichment analysis results.

STRING-FRIENDS analysis expanded the S100A12/A8 (2 gene signature) interactome to include S100A9, CDH1, AGER (RAGE receptor), and signaling adaptors (GRB2, PTPN11) (7 gene signature, Figure 10A). Functional enrichment tied these 2 genes to Calprotectin complex (Strength = 3.69), S100A9 complex (Strength = 3.69), Neutrophil aggregation, and Aquaporin 9 (Strength = 3.59), and S100A8 complex (Strength = 3.59, Figure 10B). FRIENDS analysis further revealed robust associations between 7 genes and Neutrophil aggregation, and Aquaporin 9 (Strength = 3.4 in GO Process/3.22 in STRING clusters), Toll-like receptor 4 bindings (Strength = 3.15), MET activates PTPN11 (Strength = 3.05), Calprotectin complex (Strength = 3.32), S100A9 complex (Strength = 3.32), and S100A8 complex (Strength = 3.35, Figure 11).

### 3.8 Multi-omics contextualization via GenDoma

**Figure 12:**
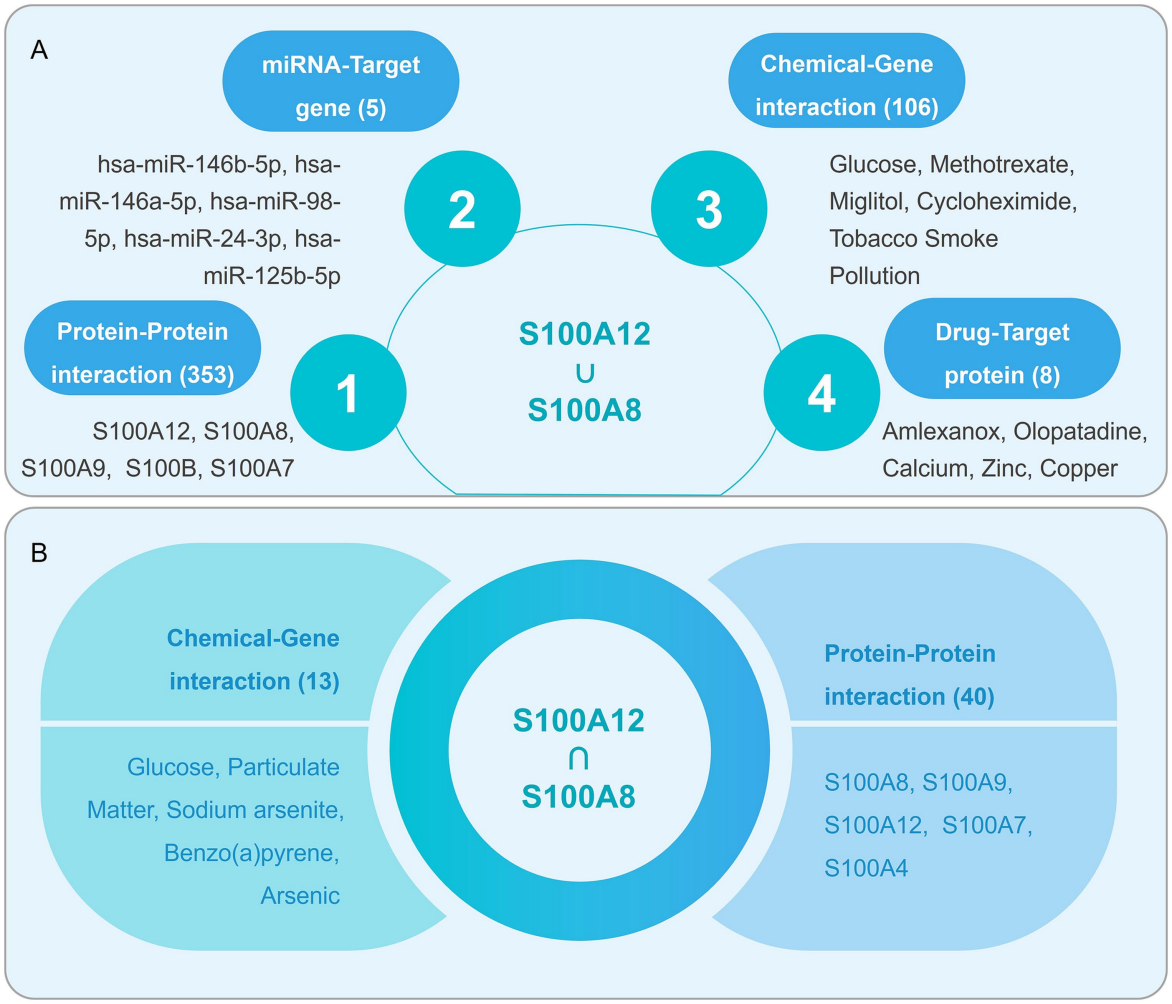
The schematic diagram for visualizing the results of high-dimensional analysis of biomarker-based on the GenDoma server and interworking network graph. **(A)** Enrichment of the PPI, mRNA-Target gene, Drug-Target protein, and Chemical-Gene in the results of the S100A12 and S100A8 concatenation analysis based on GenDoma server visualization. **(B)** PPI and Chemical-Gene enrichment in the results of the S100A12 and S100A8 intersection analyses based on GenDoma server visualization.

GenDoma revealed 353 interactions for S100A12/A8, including drug targets (e.g., tetracyclines), transcription factors (NF-κB), and disease pathways (Figures 12A,B). Literature mining highlighted their overexpression in blood dendritic cells (CD1C + B), monocytes (CD14 + CD16+), and lung basal cells (Table 3), with neutrophil depletion studies implicating S100A8/A9 in TB progression control.

**Table 3:** Literature enrichment analysis of genes.

Tissue	Cell	Biomarker	Gene	Protein ID	PMID
Blood	CD1C + _B dendritic cell	S100A12	S100A12	P80511	28428369
Peripheral blood	CD14 + CD16 + monocyte	S100A12	S100A12	P80511	29361178
Kidney	Neutrophil	S100A12	S100A12	P80511	30093597
Fetal kidney	Monocyte	S100A12	S100A12	P80511	30093597
Blood	CD1C + _B dendritic cell	S100A8	S100A8	P05109	28428369
Umbilical cord blood	Lymphoid-primed multipotent progenitor cell	S100A8	S100A8	P05109	29167569
Bone marrow	Monocyte derived dendritic cell	S100A8	S100A8	P05109	29313948
Esophagus	Secretory progenitor cell	S100A8	S100A8	P05109	29802404
Kidney	Neutrophil	S100A8	S100A8	P05109	30093597
Fetal kidney	Monocyte	S100A8	S100A8	P05109	30093597
Lung	Basal cell	S100A8	S100A8	P05109	30069046

Disease	Description	Gene	Protein ID	PMID
Tuberculosis	Depletion of neutrophils or S100A8/A9 deficiency resulted in improved MTB control during chronic but not acute TB.	S100A8	P05109	32134742

## 4 Discussion

To our knowledge, this study represents the first attempt to distinguish LTBI from ATB using a novel approach based on S100A12 and S100A8. In our study, we undertook an extensive analysis of blood transcriptomic data from 2,758 patients across 11 cohorts to identify stable differential genes that could serve as potential biomarkers for distinguishing LTBI from ATB. We focused on the S100A12 and S100A8 gene pair, which exhibited notable upregulation in ATB patients compared to those with LTBI. Our findings demonstrate the robustness of these gene signatures in diagnostic applications, as machine learning models incorporating these biomarkers achieved a significant AUC of 0.8572, indicating high predictive accuracy. Furthermore, our analysis revealed correlations between these biomarkers and immune cell populations, shedding light on their potential roles in the immune response during TB infection. These insights not only enhance our understanding of TB pathogenesis but also pave the way for future therapeutic developments aimed at improving patient outcomes (Dannenberg et al., 2000; Mitterhauser and Wadsak, 2014; Russell, 2007).

The differential expression analysis conducted across various cohorts has underscored the potential of S100A12 and S100A8 as biomarkers for distinguishing between ATB and LTBI. The identification of 55 SDGs reveals significant variability in gene expression profiles across diverse datasets, with S100A12 and S100A8 consistently exhibiting upregulation in ATB cases relative to LTBI. This notable observation indicates that these genes may serve as reliable biomarkers, enhancing diagnostic accuracy and informing treatment strategies. The variability of gene expression counts across cohorts ranging from 26 to 3,389 highlights the challenges in establishing a universal biomarker profile. However, the consistent upregulation of S100A12 and S100A8 across training cohorts suggests their potential role in the pathophysiology of TB, warranting further exploration into their mechanisms of action and clinical applicability (Li et al., 2023).

The S100 protein family, particularly S100A12 and S100A8, has garnered attention due to their roles in inflammation and immune response (Gonzalez et al., 2020). These proteins are secreted by activated immune cells and are involved in various inflammatory pathways (Donato et al., 2013). S100A8/A9 heterodimers regulate neutrophil adhesion via CD11b upregulation during MTB infection (Scott et al., 2020), while S100A12 amplifies inflammation through AGER receptor signaling (Cole et al., 2001). Studies have demonstrated that S100A12 and S100A8 are potential biomarker for disease severity and prognosis in some diseases, such as Idiopathic Pulmonary Fibrosis (Li et al., 2022), Rheumatoid Arthritis (Roszkowski et al., 2022), Blau syndrome (Wang et al., 2018), Chronic Spontaneous Urticaria (Zhou et al., 2019), active lupus nephritis (Davies et al., 2020), and dilated cardiomyopathy (Yu et al., 2024). While S100A12/S100A8 are widely studied in these diseases, their specificity to TB remains an open question. In this study, we found that the correlation between their expression levels and immune cell populations, particularly CD4^+^ T cells, neutrophils, and natural killer (NK) cells, provides insights into the immune landscape in ATB versus LTBI. Understanding the dynamics between these biomarkers and immune cell infiltration could reveal critical pathways for therapeutic intervention (Li et al., 2023; Zhuang et al., 2024a). The immune profile of ATB patients, characterized by increased neutrophil activity and altered CD4^+^ T cell responses, suggests that S100A12 and S100A8 may have immune modulatory roles, influencing the inflammatory response and disease progression. Future research directions should focus on elucidating the mechanistic pathways through which these S100 proteins interact with immune cells, potentially leading to novel therapeutic strategies targeting immune responses in TB (Gonzalez et al., 2020; Donato et al., 2013).

Functional interaction and pathway analysis further illuminate the biological significance of S100A12 and S100A8 in TB. The STRING-FRIENDS analysis indicates their involvement in pathways such as neutrophil aggregation and the calprotectin complex (Yang et al., 2024; Heilmann et al., 2019), which are essential for the host’s response to MTB infection. These findings suggest that S100A12 and S100A8 not only act as biomarkers but may also serve as targets for therapeutic intervention (Huoshen et al., 2025). The identification of additional interactions within these pathways opens avenues for drug development aimed at modulating the inflammatory response and enhancing host defense mechanisms. Considering the role of neutrophil aggregation in tuberculosis pathogenesis, targeting these pathways could potentially improve clinical outcomes for patients suffering from active disease (Heida et al., 2017).

Machine learning models utilizing the S100A12 and S100A8 gene signatures demonstrated significant predictive accuracy, with a median AUC 0.8572 in training datasets and 0.8635 in subgroup analysis, indicating their potential utility in clinical diagnostics for early detection of LTBI. The performance of various machine learning approaches highlights the importance of feature selection and model optimization in enhancing diagnostic efficacy (Li et al., 2023; Du et al., 2024). Notably, the Naïve Bayes model exhibited superior performance, suggesting its applicability in diverse clinical settings, which met WHO target product profile requirements [Global Programme on Tuberculosis and Lung Health (GTB), 2014] by (1) utilizing peripheral blood samples, (2) maintaining high sensitivity in HIV co-infected patients (AUC = 0.8490), and (3) achieving excellent discrimination in high-burden low-and middle-income country (LMIC) settings (South Africa (GSE19491 = 0.8258, GSE39940 = 0.9041, GSE40553 = 0.5875, GSE37250 = 0.8730), Malawi (GSE37250 = 0.8732, GSE39940 = 0.8747), and Asian (GSE19491_South Asian = 0.8571, GSE19491_Asian other = 0.8333)). Furthermore, subgroup analyses revealed demographic influences, with reduced prediction efficacy in males (AUC = 0.7760 ~ 0.8951 vs. Female AUC = 0.7714 ~ 0.9551) and improved performance in children individuals (GSE112104_children AUC = 1.0000 vs. Adult AUC = 0.8166 ~ 0.8873), highlighting the need for population-specific validation. The implications of these findings underscore the need for ongoing research to refine machine learning applications in TB diagnostics, paving the way for more accurate and timely identification of patients at risk for progression from LTBI to ATB (Zhao et al., 2015).

However, the validation of these biomarkers across different cohorts revealed variability in expression levels, emphasizing the complexity of biomarker validation in diverse populations (Li et al., 2023). While significant upregulation of S100A12 and S100A8 was observed in specific cohorts, inconsistent results in others may reflect demographic and clinical factors that influence biomarker expression. This variability underscores the necessity for standardized cohort definitions and careful consideration of the characteristics influencing biomarker validation. Future studies should aim to address these challenges, enhancing the robustness of biomarker discovery and validation efforts in tuberculosis research (Mester et al., 2024).

The limitations of this study primarily stem from the lack of wet lab validation, which hinders the confirmation of the identified biomarkers’ functionality. Additionally, the variability in sample size across datasets may affect the robustness of the findings and their generalizability to broader populations. The inconsistent definitions of LTBI and ATB across cohorts further complicate the analysis, leading to potential biases in classification and interpretation of results (Zhao et al., 2015; Mester et al., 2024; Zhou et al., 2023). Moreover, comorbid conditions (such as diabetes mellitus) on LTBI and the exclusion of specific cohorts may overlook critical demographic and clinical factors that could influence biomarker expression, limiting the applicability of our conclusions (Zhou et al., 2023; Kumar and Babu, 2023). Addressing these limitations through standardized definitions, enhanced sample diversity, and future mechanistic studies will be essential for validating the clinical utility of S100A12 and S100A8 in TB diagnostics.

## 5 Conclusion

In conclusion, this study successfully highlights the potential of S100A12 and S100A8 as promising biomarkers for differentiating between ATB and LTBI. The findings not only enhance diagnostic accuracy but also provide insights into the underlying immune mechanisms involved in TB infection. Furthermore, the integration of machine learning models demonstrates the feasibility of employing these biomarkers in clinical settings, paving the way for improved therapeutic strategies. Future research should focus on refining biomarker validation through comprehensive cohort analyses and mechanistic studies, ultimately contributing to better patient outcomes in tuberculosis management.

## Data Availability

The original contributions presented in the study are included in the article/Supplementary material, further inquiries can be directed to the corresponding authors.

## Footnotes

^1^ https://cran.r-project.org/web/packages/MCL/MCL.pdf

^2^ https://cibersort.stanford.edu/

^3^ https://singlecell.broadinstitute.org/

^4^ https://ai.citexs.com/homePath

^5^ https://singlecell.broadinstitute.org/single_cell/study/SCP1749/cellular-ecology-of-m-tuberculosis-granulomas-4-week-dataset#study-summary

^6^ https://singlecell.broadinstitute.org/single_cell/study/SCP257/cellular-ecology-of-m-tuberculosis-granulomas-10-week-dataset#study-summary

^7^ https://singlecell.broadinstitute.org/single_cell/study/SCP814/human-lung-hiv-tb-co-infection-ace2-cells?genes=LHFPL2&cluster=umap&spatialGroups=--&annotation=cell_type__ontology_label--group--study&subsample=all&tab=distribution#study-summary

## Glossary

LTBI: Latent tuberculosis infection
TBI: Tuberculosis infection
ATB: Active tuberculosis
LASSO: Least Absolute Shrinkage Selection Operator
SVM-RFE: Support Vector Machines Recursive Feature Elimination
MCL: Markov Cluster Algorithm
PPI: Protein–Protein Interaction
NB: Naive Bayes
AUC: Area Under Curve
IQR: Inter-Quartile Range
HIV: Human Immunodeficiency Virus
TB: Tuberculosis
AIDS: Acquired immunodeficiency syndrome
MTB: *Mycobacterium tuberculosis*
PTB: Pulmonary tuberculosis
IGRAs: Interferon-gamma release assays
TST: Tuberculin Skin Testing
HC: Health control
WHO: World Health Organization
NMR: Nuclear Magnetic Resonance
NIH GEO: National Institutes of Health Gene Expression Omnibus
DEG: Differential expression gene
SDG: Stable differential gene
ROC: Receiver Operating Characteristic
ANOVA: Analysis of variance
SVM: Support vector machines
ENR: Elastic Net Regression
MLR: Multiple Logistic Regression
RR: Ridge Regression
RFE: Recursive Feature Elimination
FDR: False Discovery Rate
DAMP: Danger-associated molecular pattern
TLR4: Toll-like receptor 4
AGER: Late glycosylation end product receptor
ROS: Reactive oxygen species
LMIC: Low-and middle-income country

## References

[ref1] An Y. Ni R. Zhuang L. Yang L. Ye Z. Li L.. (2025). Tuberculosis vaccines and therapeutic drug: challenges and future directions. Mol. Biomed. 6:4. doi: 10.1186/s43556-024-00243-6, PMID: 39841361 PMC11754781

[ref2] Chen Z. Wang T. Du J. Sun L. Wang G. Ni R.. (2024). Decoding the WHO global tuberculosis report 2024: a critical analysis of global and Chinese key data. Zoonoses 5:5. doi: 10.15212/zoonoses-2024-0061, PMID: 40244188

[ref3] Cheng P. Jiang F. Wang G. Wang J. Xue Y. Wang L.. (2023). Bioinformatics analysis and consistency verification of a novel tuberculosis vaccine candidate HP13138PB. Front. Immunol. 14:1102578. doi: 10.3389/fimmu.2023.1102578, PMID: 36825009 PMC9942524

[ref4] Cole A. M. Kim Y. H. Tahk S. Hong T. Weis P. Waring A. J.. (2001). Calcitermin, a novel antimicrobial peptide isolated from human airway secretions. FEBS Lett. 504, 5–10. doi: 10.1016/s0014-5793(01)02731-4, PMID: 11522286

[ref5] Dannenberg A. M. Bishai W. R. Parrish N. Ruiz R. Johnson W. Zook B. C.. (2000). Efficacies of BCG and vole bacillus (*Mycobacterium microti*) vaccines in preventing clinically apparent pulmonary tuberculosis in rabbits: a preliminary report. Vaccine 19, 796–800. doi: 10.1016/s0264-410x(00)00300-5, PMID: 11115701

[ref6] Davies J. C. Midgley A. Carlsson E. Donohue S. Bruce I. N. Beresford M. W.. (2020). Urine and serum S100A8/A9 and S100A12 associate with active lupus nephritis and may predict response to rituximab treatment. RMD Open 6:e001257. doi: 10.1136/rmdopen-2020-001257, PMID: 32723832 PMC7722276

[ref7] Deng J. Liu L. Yang Q. Wei C. Zhang H. Xin H.. (2021). Urinary metabolomic analysis to identify potential markers for the diagnosis of tuberculosis and latent tuberculosis. Arch. Biochem. Biophys. 704:108876. doi: 10.1016/j.abb.2021.108876, PMID: 33864753

[ref8] Donato R. Cannon B. R. Sorci G. Riuzzi F. Hsu K. Weber D. J.. (2013). Functions of S100 proteins. Curr. Mol. Med. 13, 24–57. doi: 10.2174/156652413804486214 22834835 PMC3707951

[ref9] Du J. Su Y. Qiao J. Gao S. Dong E. Wang R.. (2024). Application of artificial intelligence in diagnosis of pulmonary tuberculosis. Chin. Med. J. 137, 559–561. doi: 10.1097/cm9.0000000000003018, PMID: 38369956 PMC10932516

[ref10] Esterhuyse M. M. Weiner J. 3rd Caron E. Loxton A. G. Iannaccone M. Wagman C.. (2015). Epigenetics and proteomics join transcriptomics in the quest for tuberculosis biomarkers. mBio 6, e01187–e01115. doi: 10.1128/mBio.01187-15, PMID: 26374119 PMC4600108

[ref11] Fortún J. Navas E. (2022). Latent tuberculosis infection: approach and therapeutic schemes. Rev. Esp. Quimioter. 35, 94–96. doi: 10.37201/req/s03.20.2022, PMID: 36285867 PMC9717450

[ref12] Global Programme on Tuberculosis and Lung Health (GTB) (2014). High priority target product profiles for new tuberculosis diagnostics: Report of a consensus meeting. Geneva: World Health Organization.

[ref13] Gong W. Wu X. (2021). Differential diagnosis of latent tuberculosis infection and active tuberculosis: a key to a successful tuberculosis control strategy. Front. Microbiol. 12:745592. doi: 10.3389/fmicb.2021.745592, PMID: 34745048 PMC8570039

[ref14] Gonzalez L. L. Garrie K. Turner M. D. (2020). Role of S100 proteins in health and disease. Mol. Cell Res. 1867:118677. doi: 10.1016/j.bbamcr.2020.118677, PMID: 32057918

[ref15] HaileMariam M. Yu Y. Singh H. Teklu T. Wondale B. Worku A.. (2021). Protein and microbial biomarkers in sputum discern acute and latent tuberculosis in investigation of pastoral Ethiopian cohort. Front. Cell. Infect. Microbiol. 11:595554. doi: 10.3389/fcimb.2021.595554, PMID: 34150670 PMC8212885

[ref16] Heida A. Kobold A. C. M. Wagenmakers L. van de Belt K. van Rheenen P. F. (2017). Reference values of fecal calgranulin C (S100A12) in school aged children and adolescents. Clin. Chem. Lab. Med. 56, 126–131. doi: 10.1515/cclm-2017-0152, PMID: 28708568

[ref17] Heilmann R. M. Xenoulis P. G. Müller K. Stavroulaki E. M. Suchodolski J. S. Steiner J. M. (2019). Association of serum calprotectin (S100A8/A9) concentrations and idiopathic hyperlipidemia in miniature schnauzers. J. Vet. Intern. Med. 33, 578–587. doi: 10.1111/jvim.15460, PMID: 30788872 PMC6430953

[ref18] Huoshen W. Zhu H. Xiong J. Chen X. Mou Y. Hou S.. (2025). Identification of potential biomarkers and therapeutic targets for periodontitis. Int. Dent. J. 75, 1370–1383. doi: 10.1016/j.identj.2024.10.006, PMID: 39532570 PMC11976599

[ref19] Izquierdo-Garcia J. L. Comella-Del-Barrio P. Campos-Olivas R. Villar-Hernández R. Prat-Aymerich C. De Souza-Galvão M. L.. (2020). Discovery and validation of an NMR-based metabolomic profile in urine as TB biomarker. Sci. Rep. 10:22317. doi: 10.1038/s41598-020-78999-4, PMID: 33339845 PMC7749110

[ref20] Jiang F. Han Y. Liu Y. Xue Y. Cheng P. Xiao L.. (2023a). A comprehensive approach to developing a multi-epitope vaccine against *Mycobacterium tuberculosis*: from *in silico* design to *in vitro* immunization evaluation. Front. Immunol. 14:1280299. doi: 10.3389/fimmu.2023.1280299, PMID: 38022558 PMC10652892

[ref21] Jiang F. Liu Y. Xue Y. Cheng P. Wang J. Lian J.. (2023b). Developing a multiepitope vaccine for the prevention of SARS-CoV-2 and monkeypox virus co-infection: a reverse vaccinology analysis. Int. Immunopharmacol. 115:109728. doi: 10.1016/j.intimp.2023.109728, PMID: 36652758 PMC9832108

[ref22] Jiang F. Peng C. Cheng P. Wang J. Lian J. Gong W. (2023c). PP19128R, a multiepitope vaccine designed to prevent latent tuberculosis infection, induced immune responses *in silico* and *in vitro* assays. Vaccines 11:11. doi: 10.3390/vaccines11040856, PMID: 37112768 PMC10145841

[ref23] Jiang F. Sun T. Cheng P. Wang J. Gong W. (2023d). A summary on tuberculosis vaccine development-where to go? J. Pers. Med. 13:408. doi: 10.3390/jpm13030408, PMID: 36983589 PMC10054751

[ref24] Jiang F. Wang L. Wang J. Cheng P. Shen J. Gong W. (2023e). Design and development of a multi-epitope vaccine for the prevention of latent tuberculosis infection. Med. Adv. 1, 361–382. doi: 10.1002/med4.40

[ref25] Kaewseekhao B. Nuntawong N. Eiamchai P. Roytrakul S. Reechaipichitkul W. Faksri K. (2020). Diagnosis of active tuberculosis and latent tuberculosis infection based on Raman spectroscopy and surface-enhanced Raman spectroscopy. Tuberculosis 121:101916. doi: 10.1016/j.tube.2020.101916, PMID: 32279876

[ref26] Kumar N. P. Babu S. (2023). Impact of diabetes mellitus on immunity to latent tuberculosis infection. Front. Clin. Diabetes Healthcare 4:1095467. doi: 10.3389/fcdhc.2023.1095467, PMID: 36993821 PMC10012073

[ref27] Li Y. He Y. Chen S. Wang Q. Yang Y. Shen D.. (2022). S100A12 as biomarker of disease severity and prognosis in patients with idiopathic pulmonary fibrosis. Front. Immunol. 13:810338. doi: 10.3389/fimmu.2022.810338, PMID: 35185901 PMC8854978

[ref28] Li L. S. Yang L. Zhuang L. Ye Z. Y. Zhao W. G. Gong W. P. (2023). From immunology to artificial intelligence: revolutionizing latent tuberculosis infection diagnosis with machine learning. Mil. Med. Res. 10:58. doi: 10.1186/s40779-023-00490-8, PMID: 38017571 PMC10685516

[ref29] Li L. Zhuang L. Yang L. Ye Z. Ni R. An Y.. (2024). Machine learning model based on SERPING1, C1QB, and C1QC: a novel diagnostic approach for latent tuberculosis infection. iLABMED 2, 248–265. doi: 10.1002/ila2.65

[ref30] Lu Y. Wang X. Dong H. Wang X. Yang P. Han L.. (2019). Bioinformatics analysis of microRNA expression between patients with and without latent tuberculosis infections. Exp. Ther. Med. 17, 3977–3988. doi: 10.3892/etm.2019.7424, PMID: 30988779 PMC6447890

[ref31] Mester P. Keller D. Kunst C. Räth U. Rusch S. Schmid S.. (2024). High serum S100A12 as a diagnostic and prognostic biomarker for severity, multidrug-resistant Bacteria superinfection and herpes simplex virus reactivation in COVID-19. Viruses 16:1084. doi: 10.3390/v16071084, PMID: 39066246 PMC11281500

[ref32] Mitterhauser M. Wadsak W. (2014). Imaging biomarkers or biomarker imaging? Pharmaceuticals 7, 765–778. doi: 10.3390/ph7070765, PMID: 24967536 PMC4113731

[ref33] Natarajan S. Ranganathan M. Hanna L. E. Tripathy S. (2022). Transcriptional profiling and deriving a seven-gene signature that discriminates active and latent tuberculosis: An integrative bioinformatics approach. Genes 13:616. doi: 10.3390/genes13040616, PMID: 35456421 PMC9032611

[ref34] Peng C. Jiang F. Liu Y. Xue Y. Cheng P. Wang J.. (2024). Development and evaluation of a promising biomarker for diagnosis of latent and active tuberculosis infection. Infect. Dis. Immunity 4, 10–24. doi: 10.1097/ID9.0000000000000104 PMC13277528 42326546

[ref35] Robison H. M. Escalante P. Valera E. Erskine C. L. Auvil L. Sasieta H. C.. (2019). Precision immunoprofiling to reveal diagnostic signatures for latent tuberculosis infection and reactivation risk stratification. Integr. Biol. 11, 16–25. doi: 10.1093/intbio/zyz001, PMID: 30722034

[ref36] Roszkowski L. Jaszczyk B. Plebańczyk M. Ciechomska M. (2022). S100A8 and S100A12 proteins as biomarkers of high disease activity in patients with rheumatoid arthritis that can be regulated by epigenetic drugs. Int. J. Mol. Sci. 24:710. doi: 10.3390/ijms24010710, PMID: 36614150 PMC9820830

[ref37] Russell D. G. (2007). Who puts the tubercle in tuberculosis? Nat. Rev. Microbiol. 5, 39–47. doi: 10.1038/nrmicro1538, PMID: 17160001

[ref38] Scott N. R. Swanson R. V. Al-Hammadi N. Domingo-Gonzalez R. Rangel-Moreno J. Kriel B. A.. (2020). S100A8/A9 regulates CD11b expression and neutrophil recruitment during chronic tuberculosis. J. Clin. Invest. 130, 3098–3112. doi: 10.1172/jci130546, PMID: 32134742 PMC7259997

[ref39] Shao M. Wu F. Zhang J. Dong J. Zhang H. Liu X.. (2021). Screening of potential biomarkers for distinguishing between latent and active tuberculosis in children using bioinformatics analysis. Medicine 100:e23207. doi: 10.1097/md.0000000000023207, PMID: 33592820 PMC7870233

[ref40] Wang J. Jiang F. Cheng P. Ye Z. Li L. Yang L.. (2024). Construction of novel multi-epitope-based diagnostic biomarker HP16118P and its application in the differential diagnosis of *Mycobacterium tuberculosis* latent infection. Mol. Biomed. 5:15. doi: 10.1186/s43556-024-00177-z, PMID: 38679629 PMC11056354

[ref41] Wang L. Rosé C. D. Foley K. P. Anton J. Bader-Meunier B. Brissaud P.. (2018). S100A12 and S100A8/9 proteins are biomarkers of articular disease activity in Blau syndrome. Rheumatology 57, 1299–1304. doi: 10.1093/rheumatology/key090, PMID: 29635517

[ref42] Yang D. Chen Y. Yu Y. Chen X. (2024). Identification of genes and key pathways associated with the pathophysiology of lung Cancer and atrial fibrillation. Altern. Ther. Health Med. 30, 68–75, PMID: 37883760

[ref43] Yu Y. Shi H. Wang Y. Yu Y. Chen R. (2024). A pilot study of S100A4, S100A8/A9, and S100A12 in dilated cardiomyopathy: novel biomarkers for diagnosis or prognosis? ESC Heart Failure 11, 503–512. doi: 10.1002/ehf2.14605, PMID: 38083998 PMC10804141

[ref44] Zhao X. Pan S. Liu C. (2015). Effect of S100 calcium binding protein A12 on the pathogenesis of preeclampsia. Zhonghua Fu Chan Ke Za Zhi 50, 183–187. doi: 10.3760/cma.j.issn.0529-567x.2015.03.004, PMID: 26268407

[ref45] Zhou G. Guo X. Cai S. Zhang Y. Zhou Y. Long R.. (2023). Diabetes mellitus and latent tuberculosis infection: an updated meta-analysis and systematic review. BMC Infect. Dis. 23:770. doi: 10.1186/s12879-023-08775-y, PMID: 37940866 PMC10631079

[ref46] Zhou Q. Y. Lin W. Zhu X. X. Xu S. L. Ying M. X. Shi L.. (2019). Increased plasma levels of S100A8, S100A9, and S100A12 in chronic spontaneous Urticaria. Indian J. Dermatol. 64, 441–446. doi: 10.4103/ijd.IJD_375_18, PMID: 31896840 PMC6862366

[ref47] Zhuang L. Yang L. Li L. Ye Z. Gong W. (2024a). Mycobacterium tuberculosis: immune response, biomarkers, and therapeutic intervention. MedComm 5:e419. doi: 10.1002/mco2.419, PMID: 38188605 PMC10771061

[ref48] Zhuang L. Zhao Y. Yang L. Li L. Ye Z. Ali A.. (2024b). Harnessing bioinformatics for the development of a promising multi-epitope vaccine against tuberculosis: the ZL9810L vaccine. Decoding Infect. Transmis. 2:100026. doi: 10.1016/j.dcit.2024.100026

