# Supplementary material for: Genome-wide expression in human whole blood for diagnosis of latent tuberculosis infection: a multicohort research
Source: Front Microbiol. 2025 May 9;16:1584360. doi: 10.3389/fmicb.2025.1584360 (PMC12101067; doi:10.3389/fmicb.2025.1584360)
Supplement: Supplementary file 1 [file Data_Sheet_1.pdf]

```
####GEO表达谱的自动整理####
getwd()
#加载包
{
library(tidyverse)
#chooseBioCmirror()
#BiocManager::install('GEOquery')
library(GEOquery)
library(sva)
library(mice)
library(zoo)
library(VIM)
library(CancerSubtypes)
library(tidyverse)
library(openxlsx)
library(limma)
}
###下载数据，如果文件夹中有会直接读入
#chooseBioCmirror()
gset = getGEO('GSEGSE94438', destdir=".", AnnotGPL = T, getGPL = T)
{
#class(gset)
###提取子集
gset[[1]]
#通过pData函数获取分组信息
pdata <- pData(gset[[1]])
#write.csv(pdata,file = "group_GSE107995.csv")
table(pdata$title)
library(stringr)
#设置参考水平
group_list <- ifelse(str_detect(pdata$title,
                                "LTBI"), "LTBI","ATB")
#因子型
group_list = factor(group_list,
                    levels = c("LTBI","ATB"))

##2.2 通过exprs函数获取表达矩阵并校正
exp <- exprs(gset[[1]])
#boxplot(exp,outline=FALSE, notch=T,col=group_list, las=2)
#查看数据范围，进行log转化缩小差距
数据范围 <- range(exp)
#差距不大就不用log转换
exp <- log2(exp+1)
#负值变0
dataExpr <- pmax(exp,0)
#0变NA
dataExpr[dataExpr==0]<-NA
range(dataExpr)
##缺失值及0的处理
library(sva)
library(tidyverse)
library(mice)
library(zoo)
library(VIM)
library(CancerSubtypes)
library(tidyverse)
library(openxlsx)
library(limma)
#处理
#table(is.na(dataExpr))
index=which(is.na(dataExpr))
#res1=data.imputation(dataExpr,fun="median")
res2=data.imputation(dataExpr,fun="mean")
#table(complete.cases(res1))
table(complete.cases(res2))
```

```
#直接删掉整行全是缺失值的
#exp1 <- na.omit(res1)
exp2 <- na.omit(res2)
#矩阵转换为数据框
dataExpr <- as.data.frame(exp2)
}
#数据校正,均一化
{
library(limma)
dataExpr=normalizeBetweenArrays(dataExpr)
数据范围_处理之后 <- range(dataExpr)
exp <- dataExpr
exp<-as.data.frame(exp)
#使用R包转换id
index = gset[[1]]@annotation
#xxx.db就相当于基因是某一个国家的公民，xxx平台是这个国家的公安局
#给基因颁身份证ID的，所以读取注释用xxx.db
#平台注释见excel
GPL<-fData(gset[[1]])   ## 获取平台信息
gpl<-GPL[,c(1,3)]
gpl$`Gene symbol`<-data.frame(sapply(gpl$`Gene symbol`,
                                     function(x)unlist(strsplit(x,"///"))[1]),
                              stringsAsFactors=F)[,1]
exp$ID<-rownames(exp)   # 增加新的一列（最后一列），存放基因ID信息
#匹配ID
exp_symbol<-merge(exp,gpl,by="ID")
exp_symbol<-na.omit(exp_symbol)
table(duplicated(exp_symbol$`Gene symbol`))
exp_unique<-avereps(exp_symbol[,-c(1,ncol(exp_symbol))],
                    ID=exp_symbol$`Gene symbol`)
exp_unique<-as.data.frame(exp_unique)
}
range(exp_unique)
write.csv(exp_unique, file = "GSE39939_exp.csv")


####GEO数据库的手动使用####
getwd()
#加载R包
library(tidyverse)
library(GEOquery)
gset <- getGEO('GSE84076', destdir=".", AnnotGPL = F, getGPL = F)
{
  #class(gset)
  ###提取子集
  gset[[1]]
  #通过pData函数获取分组信息
  pdata <- pData(gset[[1]])
}
write.csv(pdata,file = "GSE84076_group.csv")
#缺失值处理
{
  table(pdata$title)
  library(stringr)
  #设置参考水平
  group_list <- ifelse(str_detect(pdata$title,
                                  "LTBI"), "LTBI","ATB")
  #因子型
  group_list = factor(group_list,
                      levels = c("LTBI","ATB"))

  ##2.2 通过exprs函数获取表达矩阵并校正
  exp <- exprs(gset[[1]])
  #boxplot(exp,outline=FALSE, notch=T,col=group_list, las=2)
  #查看数据范围，进行log转化缩小差距
```

```
  数据范围 <- range(exp)
  #差距不大就不用log转换
  exp <- log2(exp+1)
  #负值变0
  dataExpr <- pmax(exp,0)
  #0变NA
  dataExpr[dataExpr==0]<-NA
  #NaN变NA
  dataExpr[dataExpr=="NaN"]<-NA
  range(dataExpr)
  ##缺失值及0的处理
  library(sva)
  library(tidyverse)
  library(mice)
  library(zoo)
  library(VIM)
  library(CancerSubtypes)
  library(tidyverse)
  library(openxlsx)
  library(limma)
  #处理
  #table(is.na(dataExpr))
  index=which(is.na(dataExpr))
  #res1=data.imputation(dataExpr,fun="median")
  res2=data.imputation(dataExpr,fun="mean")
  #table(complete.cases(res1))
  table(complete.cases(res2))
  #直接删掉整行全是缺失值的
  #exp1 <- na.omit(res1)
  exp2 <- na.omit(res2)
  #矩阵转换为数据框
  dataExpr <- as.data.frame(exp2)
}
#数据校正,均一化
{
  library(limma)
  dataExpr=normalizeBetweenArrays(dataExpr)
  数据范围_处理之后 <- range(dataExpr)
  exp <- dataExpr
  exp<-as.data.frame(exp)
  #使用R包转换id
  index = gset[[1]]@annotation
  #xxx.db就相当于基因是某一个国家的公民，xxx平台是这个国家的公安局
  #给基因颁身份证ID的，所以读取注释用xxx.db
}
#处理
{
  #table(is.na(dataExpr))
  index=which(is.na(dataExpr))
  #res1=data.imputation(dataExpr,fun="median")
  res2=data.imputation(dataExpr,fun="mean")
  #table(complete.cases(res1))
  table(complete.cases(res2))
  #直接删掉整行全是缺失值的
  #exp1 <- na.omit(res1)
  exp2 <- na.omit(res2)
  #矩阵转换为数据框
  dataExpr <- as.data.frame(exp2)
}
数据范围_处理之后 <- range(dataExpr)
exp <- dataExpr
exp<-as.data.frame(exp)
#平台注释见excel
if(!require("illuminaHumanv4.db"))
  BiocManager::install("illuminaHumanv4.db")
```

```
#n
library(illuminaHumanv4.db)
ls("package:illuminaHumanv4.db")
ids <- toTable(illuminaHumanv4SYMBOL)
{
#head查看头几行
head(ids)

length(unique(ids$symbol))
table(sort(table(ids$symbol)))
#id转换
library(tidyverse)
#将matrix转换为数据框frame
exp <- as.data.frame(exp)
#添加探针probe_id,最后一列会添加上探针
exp <- exp %>% mutate(probe_id=rownames(exp))
#探针名匹配基因名
exp <- exp %>% inner_join(ids,by="probe_id")
#基因名去重复
exp <- exp[!duplicated(exp$symbol),]
#symbol变行名
rownames(exp) <- exp$symbol
}
#去除尾部的注释信息
exp <- exp[,-(205:206)]
#储存为文件
write.csv(exp, file = "GSE40553_exp.csv")

####0变为NA，NA再被均值填补####
#加载包
{
  library(tidyverse)
  #chooseBioCmirror()
  #BiocManager::install('GEOquery')
  library(GEOquery)
  library(sva)
  library(mice)
  library(zoo)
  library(VIM)
  library(CancerSubtypes)
  library(tidyverse)
  library(openxlsx)
  library(limma)
}
exp <- read.csv("GSE84076.csv")
rownames(exp) <- exp$Sample
exp <- exp[,-1]
#BiocManager::install("affy")
exp<-as.data.frame(exp)
数据范围 <- range(exp)
{
#差距不大就不用log转换
exp <- log2(exp+1)
#负值变0
dataExpr <- pmax(exp,0)
#0变NA
dataExpr[dataExpr==0]<-NA
range(dataExpr)
##缺失值及0的处理
library(sva)
library(tidyverse)
library(mice)
library(zoo)
library(VIM)
library(CancerSubtypes)
```

```
library(tidyverse)
library(openxlsx)
library(limma)
#处理
#table(is.na(dataExpr))
index=which(is.na(dataExpr))
#res1=data.imputation(dataExpr,fun="median")
res2=data.imputation(dataExpr,fun="mean")
#table(complete.cases(res1))
table(complete.cases(res2))
#直接删掉整行全是缺失值的
#exp1 <- na.omit(res1)
exp2 <- na.omit(res2)
#矩阵转换为数据框
dataExpr <- as.data.frame(exp2)
}
library(limma)
dataExpr=normalizeBetweenArrays(dataExpr)
数据范围_处理之后 <- range(dataExpr)
#处理
{
#table(is.na(dataExpr))
index=which(is.na(dataExpr))
#res1=data.imputation(dataExpr,fun="median")
res2=data.imputation(dataExpr,fun="mean")
#table(complete.cases(res1))
table(complete.cases(res2))
#直接删掉整行全是缺失值的
#exp1 <- na.omit(res1)
exp2 <- na.omit(res2)
#矩阵转换为数据框
dataExpr <- as.data.frame(exp2)
}
数据范围_处理之后 <- range(dataExpr)
exp <- dataExpr
exp<-as.data.frame(exp)
write.csv(exp, file = "GSE84076_exp.csv")

####热图绘制####
setwd("D:\\fan jiang\\临床\\结核生信分析\\GSE54992")
###加载R包
library(tidyverse)
library(GEOquery)
###下载数据，如果文件夹中有会直接读入
gset = getGEO('GSE84402', destdir=".", AnnotGPL = F, getGPL = F)
class(gset)
###提取子集
gset[[1]]
#通过pData函数获取分组信息
pdata <- pData(gset[[1]])
table(pdata$source_name_ch1)
library(stringr)
#设置参考水平
group_list <- ifelse(str_detect(pdata$source_name_ch1, "hepatocellular carcinoma"), "tumor",
                     "normal")
#因子型
group_list = factor(group_list,
                    levels = c("normal","tumor"))

##读取上节课整理好的表达数据exp##
exp <- read.table("exp.txt",sep = "\t",row.names = 1,check.names = F,stringsAsFactors = F,header =
T)
#差异分析
library(limma)
design=model.matrix(~group_list)
```

```
fit=lmFit(exp,design)
fit=eBayes(fit)
deg=topTable(fit,coef=2,number = Inf)
#保存DEG的结果
write.table(deg, file = "deg_all.txt",sep = "\t",row.names = T,col.names = NA,quote = F)

##标记上下调基因，1是指上下调一倍的才纳入
logFC=1
P.Value = 0.05
#k1下调，k2上调
k1 = (deg$P.Value < P.Value)&(deg$logFC < -logFC)
k2 = (deg$P.Value < P.Value)&(deg$logFC > logFC)
#$表示增加列名
deg$change = ifelse(k1,"down",ifelse(k2,"up","stable"))
#分组计数
table(deg$change)

##热图##
#!=, !+=意味着不等于,取出非stable的基因
cg = rownames(deg)[deg$change !="stable"]
#把数据取出来为diff
diff=exp[cg,]

#热图绘制
library(pheatmap)
annotation_col=data.frame(group=group_list)
rownames(annotation_col)=colnames(diff)
pheatmap(diff,
         annotation_col=annotation_col,
         scale = "row",
         show_rownames = F,
         show_colnames =F,
         color = colorRampPalette(c("navy", "white", "red"))(50),
         fontsize = 10,
         fontsize_row=3,
         fontsize_col=3)
dev.off()

####火山图绘制####
setwd("D:\\fan jiang\\临床\\结核生信分析\\GSE54992")
##读取上节课整理好的表达数据exp##
exp <- read.table("exp.txt",sep = "\t",row.names = 1,
                  check.names = F,stringsAsFactors = F,header = T)
#install.packages("ggpubr")
#install.packages("ggthemes")
library(ggpubr)
library(ggthemes)
#logFC=1
#P.Value = 0.05
ggscatter(deg,
          x = "log2FoldChange", y = "logP") +
  theme_base()

#增加基因上下调信息
ggscatter(deg, x = "log2FoldChange", y = "logP",
          color = "change",
          palette = c("blue", "black", "red"),
          size = 1) +
  theme_base()

#添加分界线
ggscatter(deg, x = "log2FoldChange", y = "logP", xlab = "log2FoldChange",
          ylab = "-log10(Adjust P-value)",
          color = "change",
          palette = c("blue", "black", "red"),
```

```
          size = 1) +
  theme_base() +
  geom_hline(yintercept = -log10(0.05), linetype = "dashed") +
  geom_vline(xintercept = c(-1, 1), linetype = "dashed")
dev.off()

#添加基因标签信息
deg$Label = ""   #新加一列label
deg <- deg[order(deg$logP), ]   #对差异基因的p值进行从小到大的排序
deg$Gene <- rownames(deg)
#高表达的基因中，选择fdr值最小的5个
up.genes <- head(deg$Gene[which(deg$change == "UP")], 5)
#低表达的基因中，选择fdr值最小的5个
down.genes <- head(deg$Gene[which(deg$change == "DOWN")], 5)
#将up.genes和down.genes合并，并加入到Label中
deg.top5.genes <- c(as.character(up.genes), as.character(down.genes))
deg$Label[match(deg.top5.genes, deg$Gene)] <- deg.top5.genes

ggscatter(deg, x = "log2FoldChange", y = "logP",
          color = "change",
          palette = c("blue", "black", "red"),
          size = 1,
          label = deg$Label,
          font.label = 8,
          repel = T,
          xlab = "log2FoldChange",
          ylab = "-log10(Adjust P-value)") +
  theme_base() +
  geom_hline(yintercept = -log10(0.05), linetype = "dashed") +
  geom_vline(xintercept = c(-1, 1), linetype = "dashed")

dev.off()


####合并数据集####
merge_eset=inner_join(exp1,exp2,
                      by="symbol")
rownames(merge_eset) <- merge_eset$symbol
merge_eset <- merge_eset[,-1]
dim(merge_eset)
exp <- as.matrix(merge_eset)
dimnames <- list(rownames(exp),colnames(exp))
data <- matrix(as.numeric(as.matrix(exp)),nrow=nrow(exp),dimnames=dimnames)
dim(data)
#查询数据类型
#class(data)
batchType <- c(rep(1,537),
               rep(2,157)
#               ,
#               rep(3,334)
)
modType <- c(rep("Control",370),rep("LTBI",167),
             rep("Control",143),rep("LTBI",14)
#             ,
#             rep("Control",280),rep("LTBI",54)
)
mod  <-  model.matrix(~as.factor(modType))
outTab <- data.frame(ComBat(data,batchType,mod,par.prior=TRUE))

write.table(outTab,file="normalize.txt",sep="\t",quote=F,col.names=F)

#合并第三个数据集进来
merge_eset=inner_join(outTab,exp3,
                      by="symbol")
rownames(merge_eset) <- merge_eset$symbol
```

```
merge_eset <- merge_eset[,-1]
dim(merge_eset)
exp <- as.matrix(merge_eset)
dimnames <- list(rownames(exp),colnames(exp))
data <- matrix(as.numeric(as.matrix(exp)),nrow=nrow(exp),dimnames=dimnames)
dim(data)
#查询数据类型
#class(data)
batchType <- c(rep(1,693),
               rep(2,334))
modType <- c(rep("Control",512),rep("LTBI",181),
             rep("Control",280),rep("LTBI",54)
)
mod  <-  model.matrix(~as.factor(modType))
outTab_final <- data.frame(ComBat(data,batchType,mod,par.prior=TRUE))
write.table(outTab_final,file="normalize_final.txt",sep="\t",quote=F,col.names=F)

####缺失值处理####
getwd()
#if (!requireNamespace("BiocManager", quietly = TRUE))
#  install.packages("BiocManager")
#BiocManager::install("sva",force =TRUE)
#n
library(sva)
library(tidyverse)
library(mice)
library(zoo)
library(VIM)
library(CancerSubtypes)
library(tidyverse)
library(openxlsx)
library(limma)
library(data.table)
{
dataExpr <- read.xlsx("综合表格.xlsx")
#dataExpr <- read.csv("GSE112104_WGCNA_Up_analysis.csv")
#删除第一横行
dataExpr <- dataExpr[-(1:1),]
#根据特定列删除重复值【去重】
dataExpr <- dataExpr %>% distinct(sample, .keep_all = TRUE)
#列名转换
rownames(dataExpr) <- dataExpr$sample
#[,]逗号前位行，逗号后为列
#删除第1到10行：df[-c(1:10),]
#删除第5到10列：df[,-c(5:10)]
#删除第一纵列
dataExpr <- dataExpr[,-(1:1)]
#负值变为0，再用均值填补
dataExpr <- pmax(dataExpr,0)
#0变为NA
dataExpr[dataExpr==0] <- NA
table(is.na(dataExpr))
index=which(is.na(dataExpr))
res1=data.imputation(dataExpr,fun="median")
res2=data.imputation(dataExpr,fun="mean")
table(complete.cases(res1))
table(complete.cases(res2))
#直接删掉整行全是缺失值的
exp1 <- na.omit(res1)
exp2 <- na.omit(res2)
#检查缺失值
table(is.na(exp1))
table(is.na(exp2))
#矩阵转换为数据框
dataExpr <- as.data.frame(exp2)
```

```
table(is.na(dataExpr))
#获取分组信息
library(stringr)
pdata <- read.xlsx("GSE41055group.xlsx")
#设置参考水平
group_list <- ifelse(str_detect(pdata$title,
                                "ATB"), "ATB","LTBI")
#因子型
group_list = factor(group_list,
                    levels = c("LTBI","ATB"))

###数据校正,均一化?
library(limma)
dataExpr=normalizeBetweenArrays(dataExpr)
boxplot(dataExpr,outline=FALSE, notch=T,col=group_list, las=2)

#查看数据范围，进行log转化缩小差距
range(dataExpr)

#log2逆转换
#dataExpr=2^dataExpr
#差距不大就不用log转换
#dataExpr <- log2(dataExpr+1)
}

range(dataExpr)
dev.off()
write.csv(dataExpr,"GSE41055.csv")

####LASSO回归####
getwd()
#setwd("D:\\fan jiang\\临床\\结核生信\\结核生信分析20230312\\LASSO")
rm(list=ls())
#加载包
{
library(glmnet)
library(readxl)
library(plyr)
library(caret)
library(corrplot)
library(ggplot2)
library(Hmisc)
library(openxlsx)
library(Cairo)
}
#创建文件夹
if(!dir.exists("GSE40553分析结果")){dir.create("GSE40553分析结果")}
#读取数据
data <- read.csv("GSE40553_exp.csv")
rownames(data) <- data$X
data <- data[,-1]
data <- t(data)
data <- as.data.frame(data)
group <- read.csv("GSE40553_group.csv")
rownames(group) <- group$geo_accession
group <- group[,-(2:9)]
group <- group[,3:12]
#整合数据集
LASSO <- cbind(group,data)
LASSO <- LASSO[,-1]
library(dplyr)
LASSO_1 <- LASSO %>% filter(grepl('ATB|LTBI',
                                  group))
#ATB=1,LTBI=0, active tuberculosis, latent TB infection
LASSO_1[LASSO_1=="ATB"]<-1
```

```
LASSO_1[LASSO_1=="LTBI"]<-0
#LASSO_1 <- LASSO_1[,-(1:6)]
#data <- read.xlsx("GSE39939_WGCNA_Down_LASSO.xlsx")
#disease.state.ch1
x <- as.matrix(LASSO_1[,-1])
y <- as.double(LASSO_1$`group`)
#alpha取值为0、1或0~1范围数字,
#“0”代表岭回归Ridge regression,
#岭回归不会将较低的集降为零，所以不能用于数据的降维处理
#“1”代表LASSO回归,
#0~1数值表示弹性网络回归Elastic-net regression,
#lambda为正则化参数
fit <- glmnet
fit <- glmnet(x = x,y = y,family="binomial",nlambda = 1000, alpha = 1)
print(fit)
plot(fit,xvar = "lambda")
#print(plot="GSE37250分析结果/GSE37250_WGCNA_Down - LASSO - lambda.jpg")
dev.off()

lasso_fit <- cv.glmnet(x,y,family="binomial", alpha = 1,
                       type.measure = "auc",
                       nlambda = 1000)
plot(lasso_fit)
dev.off()
{
lasso_best <- glmnet(x=x,y=y,alpha = 1,
                     lambda = lasso_fit$lambda.min)
coef(lasso_best)
#结果中有数值的表示是好的变量
coefficient <- coef(lasso_best,s=lasso_best$lambda.min)
coe <- coefficient@x
coe <- as.data.frame(coe)
Active_Index <- which(as.numeric(coefficient)!=0)
active_coefficients <- as.numeric(coefficient)[Active_Index]
variable <- rownames(coefficient)[Active_Index]
variable <- as.data.frame(variable)
variable <- cbind(variable,coe)
}
#查看variable即为合格变量，后面的数值为回归系数,输出csv
write.csv(variable,"GSE40553分析结果/Variable.csv")

####SVM-RFE支持向量机-特征递归消除####
#特征递归消除(RFE，recursive feature elimination) RFE 算法通过增加或移除特定特征变量获得能
#最大化模型性能的最优组合变量。通过将我们的数据进行多次的训练，每一次训练后，
#都会根据权值系数来移除权重比较低的特征，此时，再根据新的特征，继续下一轮的特征，
#这就是递归特征消除这里利用随机森林-RFE挑选最优脂质标志物组合。
#用到的数据格式同LASSO
getwd()
#加载工具包
{
  library(ggplot2)
  library(cowplot)
  library(reshape2)
  library(dplyr)
  library(inflection)
  library(pROC)
  library(openxlsx)
  suppressWarnings(library(caret))
  suppressWarnings(library(randomForest))

  color=c("#dc5a3c","#6eb4b4")
}
  if(!dir.exists("SVM")){dir.create("SVM")}
#读取数据
data <- read.csv("GSE40553_exp.csv")
```

```
rownames(data) <- data$X
data <- data[,-1]
data <- t(data)
data <- as.data.frame(data)
group <- read.csv("GSE40553_group.csv")
rownames(group) <- group$geo_accession
group <- group[,-2]
group <- group[,-(1:3)]
group <- group[,-(3:30)]
#整合数据集
LASSO <- cbind(group,data)
LASSO <- LASSO[,-1]
library(dplyr)
group <- group %>% filter(grepl('ATB|LTBI',group))
LASSO_1 <- LASSO %>% filter(grepl('ATB|LTBI',group))
#LASSO_1[LASSO_1=="ATB"]<-1
#LASSO_1[LASSO_1=="LTBI"]<-0
group$group=factor(group$group,levels = unique(group$group))
dat <- LASSO_1
dat$group=as.factor(group$group)
#设置参数
{
set.seed(123)
#linear regression= lmFuncs
#random forests=rfFuncs
#naive Bayes=nvFuncs
#bagged trees=treebagged
#control <- rfeControl(functions=rfFuncs,method = "repeatdcv",saveDetails = T,
#                       verbose=T,returnResamp = "all",p=0.7,allowParallel=T,
#                       number=10,repeats=10)
control <- rfeControl(
    functions = rfFuncs,
    rerank = FALSE,
    method = "repeatedcv",
    saveDetails = T,
    number = 10,
    repeats = 10,
    verbose = T,
    returnResamp = "all",
    p = 0.7,
    index = NULL,
    indexOut = NULL,
    timingSamps = 0,
    seeds = 123,
    allowParallel = TRUE)
}
#number=10即十折交叉检验
set.seed(123)
rfe.train <- rfe(dat[,-ncol(dat)],
                 dat$group,
                 saveDetails=TRUE,
                 sizes=c(2,4,6,8, seq(10,40,by=3)),
                 rfeControl = rfeControl(functions = caretFuncs,
                                         method = "cv"),
                 methods="svmRadial")
save.image("SVM/rfe.Rdata")
{
#计算所有的重要性
importance=rfe.train$variables %>% group_by(var) %>%
  summarize(Overall=mean(Overall)) %>%
  arrange(-Overall)
write.csv(importance,file="SVM/生物标志物重要性顺序表格.csv")

#找寻关键biomarker
optsize=rfe.train$optsize
```

```
accuracy=rfe.train$results
elbow=uik(accuracy$Variables,accuracy$Accuracy)
n=min(optsize,elbow)
#plot accuracy plot
p1=ggplot(data=accuracy,
          mapping=aes(x=Variables,
                      y=Accuracy*100))+
  geom_line()+
  geom_point(size=4)+
  theme_bw()+
  geom_vline(xintercept=n,
             color="red",
             linetype=2)+
  ylab("Accuracy %(cross-validation)")+
  theme(panel.grid=element_line(color="grey",
                                linetype=2,
                                size=0.1),
        panel.background=element_rect(color="white"))
ggsave(p1,file="SVM/生物标志物组合重要性曲线.pdf",width=8,height=6)

#获得生物标志物变量
var=importance$var[1:n]
d=dat[,var]
d$group=factor(dat$group)
write.csv(d,file = "SVM/生物标志物最终最优组合.csv")
} #基本分析已经结束

#生物标志物PCA分析
data_trans=as.data.frame(d)
color=c("#00478e","#644909",
                 "#4a4c5b",
                 "#995e68",
                 "#3c5488b2",
                 "#00a087b2")
                 color_number=length(unique(data_trans$group))
                 data_trans$group = factor(data_trans$group,
                                           levels=unique(data_trans$group))
                 data=data_trans[,ncol(data_trans)]
                 pca <- prcomp(data)
                 df <- as.data.frame(pca$x)
                 df$group <- group$group
                 y <- summary(pca)
                 weight <- y$importance[2,]
                 p2 <- ggplot(df,aes(PC1,PC2,color=gourp))+
                   theme_classic()+
                   geom_vline(xintercept=0,color="grey",size=0.3)+
                   geom_hline(yintercept=0,color="grey",size=0.3)+
                   geom_point(size=5)+
                   scale_color_manual(values=colors)+
                   theme(panel.grid=element_line(color="grey",linetype=2,size=0.1),
                         panel.background=element_rect(color="black",fill='transparent'),
                         legend/title=element_blank(),
                         legend.text=element_text(size=20),
                         axis.text=element_text(size=18),
                         axis.title=element_text(size=20),legend.position='right')+
                   #ellipse circle
                   stat_ellipse(type="t",linetype=5,level=0.9)+
                   labs(x=paste('PCA1:',round(weight[1]*100,2),'%'),
                        y=paste('PCA2:',round(weight[2]*100,2),'%'))
                 ggsave(file = "分析结果/
            生物标志物最终最优组合_PCA.pdf",plot=p2,width=10,height=8)


####单基因免疫浸润分析####
#library(devtools)
```

```
#if(!require(CIBERSORT))devtools::install_github("Moonerss/CIBERSORT")
#1
getwd()
setwd("D:\\fan jiang\\临床\\结核生信\\
      结核生信分析20230312\\estimation_timer2.0")
library(CIBERSORT)
library(ggplot2)
library(pheatmap)
library(ggpubr)
library(reshape2)
library(tidyverse)
library(glmnet)
library(readxl)
library(plyr)
library(caret)
library(corrplot)
library(ggplot2)
library(Hmisc)
library(openxlsx)
data1 <- read.xlsx("estimation.xlsx",rowNames = 1)
data2 <- read.xlsx("group.xlsx",rowNames = 1)

#看一下数据表达的情况
boxplot(data1,outline=F,notch=F,las=2)

#如果均数不一致需标准化
library(limma)
data1=normalizeBetweenArrays(data1)
boxplot(data1,outline=F,notch=F,las=2)

####RNAseqTool####
####交互式RNAseq数据分析ShinyApp####
# Install devtools
install.packages("devtools")
# Install BiocManager
install.packages("BiocManager")

# Install dependent R packages from Bioconductor
pkgs <- c('clusterProfiler', 'DESeq2', 'Mfuzz')
lapply(pkgs,function(pkg){
  if (!require(pkg, quietly = TRUE))
    BiocManager::install(pkg,update = F)
})
# Install dependent R packages from GitHub
devtools::install_github("vqv/ggbiplot", force = TRUE)
1
devtools::install_github("junjunlab/GseaVis", force = TRUE)

# Install RNAseqTool
devtools::install_github("ChaoXu1997/RNAseqTool")
# Run ShinyApp
RNAseqTool::run_app()

# Remove RNAseqTool
# remove.packages("RNAseqTool")


####drawCell####
#教程链接如下#
#https://mp.weixin.qq.com/s/ZWPkxH4QxmXDktjKMl6MkA#
rm(list = ls())
# devtools::install_github("svalvaro/drawCell")
library(drawCell)
library(tidyverse)
drawCell::drawCellShiny()
```

```
####绘制多重ROC####
#加载R包
#remotes::install_github("cardiomoon/multipleROC")
#2
#remotes::install_git("https://gitee.com/swcyo/multipleROC/")
#绘制单一ROC
{
data <- read.xlsx("箱式图 for ROC.xlsx",sheet = "GSE37250")
library(multipleROC)
df <- as.data.frame(data)
p <- multipleROC(Group~GBP5,data=df)
plot_ROC(p,
         show.points = T,
         show.eta = T,
         show.sens = T,
         show.AUC = T,
         facet = F )
p$auc
p$cutpoint
p$cutoff
}

getwd()
library(multipleROC)
library(openxlsx)

data <- read.csv("GSE37250_exp.csv")
rownames(data) <- data$X
data <- data[,-1]
data <- t(data)
data <- as.data.frame(data)
group <- read.csv("GSE37250_group.csv")
rownames(group) <- group$geo_accession
group <- group[,-2]
group <- group[,-(1:3)]
group <- group[,-(3:30)]
#整合数据集
LASSO <- cbind(group,data)
LASSO <- LASSO[,-1]
library(dplyr)
group <- group %>% filter(grepl('ATB|LTBI',group))
LASSO_1 <- LASSO %>% filter(grepl('ATB|LTBI',group))
LASSO_1[LASSO_1=="ATB"]<-1
LASSO_1[LASSO_1=="LTBI"]<-0
data <- LASSO_1
#多条ROC一同显示
plot_ROC2(yvar="group",
          xvars=c("ANXA3","GPR84","MCEMP1",
                  "MMP9","S100A12","S100A8",
                  "GBP1","GBP5","IFI27",
                  "IFIT3","PLSCR1","RSAD2",
                  "AIM2","CXCR5","NAIP",
                  "NLRC4","BPI","DEFA4",
                  "ELANE","C1QA","FCGBP",
                  "SERPING1","FCAR","FCGR1A",
                  "FCGR1B","LCN2","VNN1",
                  "COL17A1","PLOD2","CYP1B1",
                  "MGST1"),
          dataname="data")

#绘制一个数据集的多个ROC
library(pROC)
p1 <- roc(data$group,data$GBP5)
p2 <- roc(data$group,data$LHFPL2)
```

```
p3 <- roc(data$group,data$NPC2)
plot(smooth(p1),col="#ffb3ffff",legacy.axes=T)
plot(p2,col="#0080ffff",add=T)
plot(p3,col="#00AD9A",add=T)

##配色板
"#E16A86" "#909800" "#00AD9A" "#9183E6" "#ffb3ffff" "#0080ffff"

#求出每个曲线的AUC
AUC1 <- round(auc(p1),3)##AUC
#round(ci(p1),3)##95%CI
AUC2 <- round(auc(p2),3)##AUC
#round(ci(p2),3)##95%CI
AUC3 <- round(auc(p3),3)##AUC
#round(ci(p3),3)##95%CI
#添加图例
legend("bottomright",title = "GSE",
       legend = c("GBP5:     0.498",
                  "LHFPL2:  0.513",
                  "NPC2:      0.49"),
       col=c("#ffb3ffff","#0080ffff","#00AD9A"),
       lty=1)
#保存图片

#ROC面积间的差异检验
roc.test(p1,p3)

#多个ROC曲线分页绘制
p1 <- multipleROC(Group~GBP5,data=data1)
p2 <- multipleROC(Group~LHFPL2,data=data1)
p3 <- multipleROC(Group~NPC2,data=data1)

p4 <- multipleROC(Group~GBP5,data=data2)
p5 <- multipleROC(Group~LHFPL2,data=data2)
p6 <- multipleROC(Group~NPC2,data=data2)

p7 <- multipleROC(Group~GBP5,data=data3)
p8 <- multipleROC(Group~LHFPL2,data=data3)
p9 <- multipleROC(Group~NPC2,data=data3)

p10 <- multipleROC(Group~GBP5,data=data4)
p11 <- multipleROC(Group~LHFPL2,data=data4)
#p12 <- multipleROC(Group~NPC2,data=data4)

p13 <- multipleROC(Group~GBP5,data=data5)
p14 <- multipleROC(Group~LHFPL2,data=data5)
#p15 <- multipleROC(Group~NPC2,data=data5)

p16 <- multipleROC(Group~GBP5,data=data6)
p17 <- multipleROC(Group~LHFPL2,data=data6)
p18 <- multipleROC(Group~NPC2,data=data6)

p19 <- multipleROC(Group~GBP5,data=data7)
p20 <- multipleROC(Group~LHFPL2,data=data7)
p21 <- multipleROC(Group~NPC2,data=data7)

p22 <- multipleROC(Group~GBP5,data=data8)
p23 <- multipleROC(Group~LHFPL2,data=data8)
p24 <- multipleROC(Group~NPC2,data=data8)

p25 <- multipleROC(Group~GBP5,data=data9)
p26 <- multipleROC(Group~LHFPL2,data=data9)
p27 <- multipleROC(Group~NPC2,data=data9)

p28 <- multipleROC(Group~GBP5,data=data10)
```

```
p29 <- multipleROC(Group~LHFPL2,data=data10)
p30 <- multipleROC(Group~NPC2,data=data10)


plot_ROC(list(p1,p2,p3,p4,p5,p6,p7,p8,p9,p10,
              p11,
              #p12,
              p13,p14,
              #p15,
              p16,p17,p18,p19,p20,
              p21,p22,p23,p24,p25,p26,p27,p28,p29,p30),
         show.points = T,
         show.eta = F,
         show.sens = F,
         show.AUC = F,
         facet = T)

ROC统计结果<- data.frame("参数"=c("cutpoint","sens","auc"),
                   "GBP5-1"=c(p1[["cutpoint"]],
                             p1[["sens"]],
                             p1[["spec"]],
                             p1[["auc"]]),
                   "GBP5-2"=c(p4[["cutpoint"]],
                             p4[["sens"]],
                             p4[["spec"]],
                             p4[["auc"]]),
                   "GBP5-3"=c(p7[["cutpoint"]],
                             p7[["sens"]],
                             p7[["spec"]],
                             p7[["auc"]]),
                   "GBP5-4"=c(p10[["cutpoint"]],
                             p10[["sens"]],
                             p10[["spec"]],
                             p10[["auc"]]),
                   "GBP5-5"=c(p13[["cutpoint"]],
                             p13[["sens"]],
                             p13[["spec"]],
                             p13[["auc"]]),
                   "GBP5-6"=c(p16[["cutpoint"]],
                             p16[["sens"]],
                             p16[["spec"]],
                             p16[["auc"]]),
                   "GBP5-7"=c(p19[["cutpoint"]],
                             p19[["sens"]],
                             p19[["spec"]],
                             p19[["auc"]]),
                   "GBP5-8"=c(p22[["cutpoint"]],
                             p22[["sens"]],
                             p22[["spec"]],
                             p22[["auc"]]),
                   "GBP5-9"=c(p25[["cutpoint"]],
                             p25[["sens"]],
                             p25[["spec"]],
                             p25[["auc"]]),
                   "GBP5-10"=c(p28[["cutpoint"]],
                             p28[["sens"]],
                             p28[["spec"]],
                             p28[["auc"]]),
                   "LHFPL2-1"=c(p2[["cutpoint"]],
                              p2[["sens"]],
                              p2[["spec"]],
                              p2[["auc"]]),
                   "LHFPL2-2"=c(p5[["cutpoint"]],
                              p5[["sens"]],
                              p5[["spec"]],
```

```
           p5[["auc"]]),
"LHFPL2-3"=c(p8[["cutpoint"]],
           p8[["sens"]],
           p8[["spec"]],
           p8[["auc"]]),
"LHFPL2-4"=c(p11[["cutpoint"]],
           p11[["sens"]],
           p11[["spec"]],
           p11[["auc"]]),
"LHFPL2-5"=c(p14[["cutpoint"]],
           p14[["sens"]],
           p14[["spec"]],
           p14[["auc"]]),
"LHFPL2-6"=c(p17[["cutpoint"]],
           p17[["sens"]],
           p17[["spec"]],
           p17[["auc"]]),
"LHFPL2-7"=c(p20[["cutpoint"]],
           p20[["sens"]],
           p20[["spec"]],
           p20[["auc"]]),
"LHFPL2-8"=c(p23[["cutpoint"]],
           p23[["sens"]],
           p23[["spec"]],
           p23[["auc"]]),
"LHFPL2-9"=c(p26[["cutpoint"]],
           p26[["sens"]],
           p26[["spec"]],
           p26[["auc"]]),
"LHFPL2-10"=c(p29[["cutpoint"]],
           p29[["sens"]],
           p29[["spec"]],
           p29[["auc"]]),
"NPC2-1"=c(p3[["cutpoint"]],
         p3[["sens"]],
         p3[["spec"]],
         p3[["auc"]]),
"NPC2-2"=c(p6[["cutpoint"]],
         p6[["sens"]],
         p6[["spec"]],
         p6[["auc"]]),
"NPC2-3"=c(p9[["cutpoint"]],
         p9[["sens"]],
         p9[["spec"]],
         p9[["auc"]]),
         #p12[["cutpoint"]],
         #p12[["sens"]],
         #p12[["spec"]],
         #p12[["auc"]],
         #p15[["cutpoint"]],
         #p15[["sens"]],
         #p15[["spec"]],
         #p15[["auc"]],
"NPC2-6"=c(p18[["cutpoint"]],
         p18[["sens"]],
         p18[["spec"]],
         p18[["auc"]]),
"NPC2-7"=c(p21[["cutpoint"]],
         p21[["sens"]],
         p21[["spec"]],
         p21[["auc"]]),
"NPC2-8"=c(p24[["cutpoint"]],
         p24[["sens"]],
         p24[["spec"]],
         p24[["auc"]]),
```

```
                  "NPC2-9"=c(p27[["cutpoint"]],
                          p27[["sens"]],
                          p27[["spec"]],
                          p27[["auc"]]),
                  "NPC2-10"=c(p30[["cutpoint"]],
                          p30[["sens"]],
                          p30[["spec"]],
                          p30[["auc"]]))
write.xlsx(ROC统计结果,"ROC统计结果.xlsx")

####WGCNA####
#教程如下
#https://mp.weixin.qq.com/s?
__biz=MzI5MTcwNjA4NQ==&mid=2247495004&idx=2&sn=f642766c3c78e716012820383bef6c08&chksm=ec0e28d6db79a1
#安装包
if(!require("https://bioconductor.org/biocLite.R"))
  BiocManager::install("https://bioconductor.org/biocLite.R")
#source("https://bioconductor.org/biocLite.R")
biocLite(c("AnnotationDbi", "impute","GO.db", "preprocessCore"))
site="https://mirrors.tuna.tsinghua.edu.cn/CRAN"
install.packages(c("WGCNA", "stringr", "reshape2"), repos=site)

#start
getwd()
###加载包
{
library(WGCNA)
library(dynamicTreeCut)
library(fastcluster)
library(reshape2)
library(stringr)
library(openxlsx)
library(dplyr)
}

#
options(stringsAsFactors = FALSE)
# 打开多线程
enableWGCNAThreads()
##导入数据##
#dataExpr <- read.xlsx("GSE37250_WGCNA_Up.xlsx")
dataExpr <- read.csv("GSE37250_exp.csv")
rownames(dataExpr) <- dataExpr$X
#删除第一纵列
dataExpr <- dataExpr[,-(1:1)]
View(dataExpr)
### 转换为样品在行，基因在列的矩阵
dataExpr <- as.data.frame(t(dataExpr))
### 检测缺失值
gsg = goodSamplesGenes(dataExpr, verbose = 3)
if (!gsg$allOK){
  # Optionally, print the gene and sample names that were removed:
  if (sum(!gsg$goodGenes)>0)
    printFlush(paste("Removing genes:",
                     paste(names(dataExpr)[!gsg$goodGenes], collapse = ",")));
  if (sum(!gsg$goodSamples)>0)
    printFlush(paste("Removing samples:",
                     paste(rownames(dataExpr)[!gsg$goodSamples], collapse = ",")));
  # Remove the offending genes and samples from the data:
  dataExpr = dataExpr[gsg$goodSamples, gsg$goodGenes]
}
nGenes = ncol(dataExpr)
nSamples = nrow(dataExpr)
dim(dataExpr)
```

```
### 查看是否有离群样品
sampleTree = hclust(dist(dataExpr), method = "average")
plot(sampleTree,
     main = "Sample clustering to detect outliers",
     sub="",
     xlab="")
dev.off()

#软阈值的筛选原则是使构建的网络更符合无标度网络特征
powers = c(c(1:10), seq(from = 12, to=30, by=2))
sft = pickSoftThreshold(dataExpr,
                        powerVector=powers,
                        networkType = "unsigned",
                        verbose=5)#稍微费点时间
par(mfrow = c(1,2))
cex1 = 0.9
# 横轴是Soft threshold (power)，纵轴是无标度网络的评估参数，数值越高，
# 网络越符合无标度特征 (non-scale)
plot(sft$fitIndices[,1], -sign(sft$fitIndices[,3])*sft$fitIndices[,2],
     xlab="Soft Threshold (power)",
     ylab="Scale Free Topology Model Fit,signed R^2",type="n",
     main = paste("Scale independence"))
text(sft$fitIndices[,1], -sign(sft$fitIndices[,3])*sft$fitIndices[,2],
     labels=powers,cex=cex1,col="red")
# 筛选标准。R-square=0.85
abline(h=0.85,col="red")

# Soft threshold与平均连通性
plot(sft$fitIndices[,1], sft$fitIndices[,5],
     xlab="Soft Threshold (power)",ylab="Mean Connectivity", type="n",
     main = paste("Mean connectivity"))
text(sft$fitIndices[,1], sft$fitIndices[,5], labels=powers,
     cex=cex1, col="red")
power = sft$powerEstimate
power

###经验power (无满足条件的power时选用)
# 无向网络在power小于15或有向网络power小于30内，没有一个power值可以使
# 无标度网络图谱结构R^2达到0.8，平均连接度较高如在100以上，可能是由于
# 部分样品与其他样品差别太大。这可能由批次效应、样品异质性或实验条件对
# 表达影响太大等造成。可以通过绘制样品聚类查看分组信息和有无异常样品。
# 如果这确实是由有意义的生物变化引起的，也可以使用下面的经验power值。
if (is.na(power)){
  power = ifelse(nSamples<20, ifelse(type="unsigned", 9, 18),
                 ifelse(nSamples<30, ifelse(type="unsigned", 8, 16),
                        ifelse(nSamples<40, ifelse(type="unsigned", 7, 14),
                               ifelse(type="unsigned", 6, 12))
                 )
  )
}

###一步法网络构建：One-step network construction and module detection##
# power: 上一步计算的软阈值
# maxBlockSize: 计算机能处理的最大模块的基因数量 (默认5000)；
#  4G内存电脑可处理8000-10000个，16G内存电脑可以处理2万个，32G内存电脑可
#  以处理3万个
#  计算资源允许的情况下最好放在一个block里面。
# corType: pearson or bicor
# numericLabels: 返回数字而不是颜色作为模块的名字，后面可以再转换为颜色
# saveTOMs：最耗费时间的计算，存储起来，供后续使用
# mergeCutHeight: 合并模块的阈值，越大模块越少
net = blockwiseModules(dataExpr,
                       power = 10,
                       maxBlockSize = 5000,
                       TOMType = "signed",
```

```
                      minModuleSize = 30,#20/25
                      reassignThreshold = 0,
                      mergeCutHeight = 0.25,
                      numericLabels = TRUE,
                      pamRespectsDendro = FALSE,
                      saveTOMs=TRUE,
                      saveTOMFileBase = "MyTOM",
                      corType = "bicor",
                      maxPOutliers= 0,
                      loadTOMs=TRUE,
                      verbose = 3)
# 根据模块中基因数目的多少，降序排列，依次编号为 `1-最大模块数`。
# **0 (grey)**表示**未**分入任何模块的基因。
table(net$colors)
#绘制模块聚类图
mergedColors = labels2colors(net$colors)
table(mergedColors)
pdf(file="5_Dynamic Tree Cut.pdf",width = 8,height = 6)
plotDendroAndColors(net$dendrograms[[1]],
                    mergedColors[net$blockGenes[[1]]],
                                 "Module colors",
                    dendroLabels = F,
                    hang = 0.03,
                    addGuide = T,
                    guideHang = 0.05)
dev.off()

###层级聚类树展示各个模块
## 灰色的为**未分类**到模块的基因。
# Convert labels to colors for plotting
moduleLabels = net$colors
moduleColors = labels2colors(moduleLabels)
# Plot the dendrogram and the module colors underneath
# 如果对结果不满意，还可以recutBlockwiseTrees，节省计算时间
plotDendroAndColors(net$dendrograms[[1]], moduleColors[net$blockGenes[[1]]],
                    "Module colors",
                    dendroLabels = FALSE, hang = 0.03,
                    addGuide = TRUE, guideHang = 0.05)

###绘制模块之间相关性图
# module eigengene, 可以绘制线图，作为每个模块的基因表达趋势的展示
MEs = net$MEs

### 不需要重新计算，改下列名字就好
### 官方教程是重新计算的，起始可以不用这么麻烦
if(!require('stringr')) {
  install.packages('stringr')
  library('stringr')
}
MEs_col = MEs
colnames(MEs_col) = paste0("ME", labels2colors(
  as.numeric(str_replace_all(colnames(MEs),"ME",""))))
MEs_col = orderMEs(MEs_col)

### 根据基因间表达量进行聚类所得到的各模块间的相关性图
# marDendro/marHeatmap 设置下、左、上、右的边距
plotEigengeneNetworks(MEs_col, "Eigengene adjacency heatmap",
                      marDendro = c(3,3,2,4),
                      marHeatmap = c(3,4,2,2),
                      plotDendrograms = T,
                      xLabelsAngle = 90)

## 如果有表型数据，也可以跟ME数据放一起，一起出图
#traitData <- read.xlsx("group.xlsx")
#MEs_colpheno = orderMEs(cbind(MEs_col, traitData))
```

```
#plotEigengeneNetworks(MEs_colpheno, "Eigengene adjacency heatmap",
#                      marDendro = c(3,3,2,4),
#                      marHeatmap = c(3,4,2,2), plotDendrograms = T,
#                      xLabelsAngle = 90)

###可视化基因网络 (TOM plot)
# 如果采用分步计算，或设置的blocksize>=总基因数，直接load计算好的TOM结果
# 否则需要再计算一遍，比较耗费时间
# TOM = TOMsimilarityFromExpr(dataExpr, power=power, corType=corType, networkType=type)
load(net$TOMFiles[1], verbose=T)
TOM <- as.matrix(TOM)

dissTOM = 1-TOM
# Transform dissTOM with a power to make moderately strong
# connections more visible in the heatmap
plotTOM = dissTOM^7
# Set diagonal to NA for a nicer plot
diag(plotTOM) = NA
# Call the plot function

### 这一部分特别耗时，行列同时做层级聚类
TOMplot(plotTOM, net$dendrograms, moduleColors,
        main = "Network heatmap plot, all genes")

###导出网络用于Cytoscape
probes = colnames(dataExpr)
dimnames(TOM) <- list(probes, probes)

# Export the network into edge and node list files Cytoscape can read
# threshold 默认为0.5, 可以根据自己的需要调整，也可以都导出后在
# cytoscape中再调整
cyt = exportNetworkToCytoscape(TOM,
                               edgeFile = paste(exprMat, ".edges.txt", sep=""),
                               nodeFile = paste(exprMat, ".nodes.txt", sep=""),
                               weighted = TRUE, threshold = 0,
                               nodeNames = probes, nodeAttr = moduleColors)
dev.off()

####多平台数据集强行合并####
getwd()
#if (!requireNamespace("BiocManager", quietly = TRUE))
#  install.packages("BiocManager")
#BiocManager::install("sva",force =TRUE)
#n
library(sva)
library(tidyverse)
library(mice)
library(zoo)
library(VIM)
library(CancerSubtypes)
library(tidyverse)
library(openxlsx)
library(limma)
library(data.table)
{
  dataExpr <- read.xlsx("综合表格.xlsx")
  #dataExpr <- read.csv("GSE112104_WGCNA_Up_analysis.csv")
  #获取分组信息
  library(stringr)
  pdata <- read.xlsx("综合表格group.xlsx")
  #设置参考水平
  group_list <- ifelse(str_detect(pdata$title,
                                  "ATB"), "ATB","LTBI")
  #因子型
  group_list = factor(group_list,
```

```
                        levels = c("LTBI","ATB"))
  #删除第一横行
  dataExpr <- dataExpr[-(1:1),]
  #根据特定列删除重复值【去重】
  dataExpr <- dataExpr %>% distinct(sample, .keep_all = TRUE)
  #列名转换
  rownames(dataExpr) <- dataExpr$sample
  #[,]逗号前位行，逗号后为列
  #删除第1到10行：df[-c(1:10),]
  #删除第5到10列：df[,-c(5:10)]
  #删除第一纵列
  dataExpr <- dataExpr[,-(1:1)]
  #负值变为0，再用均值填补
  dataExpr <- pmax(dataExpr,0)
  #0变为NA
  dataExpr[dataExpr==0] <- NA
  table(is.na(dataExpr))
  index=which(is.na(dataExpr))
  res1=data.imputation(dataExpr,fun="median")
  res2=data.imputation(dataExpr,fun="mean")
  table(complete.cases(res1))
  table(complete.cases(res2))
  #直接删掉整行全是缺失值的
  exp1 <- na.omit(res1)
  exp2 <- na.omit(res2)
  #检查缺失值
  table(is.na(exp1))
  table(is.na(exp2))
  #矩阵转换为数据框
  dataExpr <- as.data.frame(exp2)
  table(is.na(dataExpr))

  #数据的长宽转换
  library(tidyverse)
  library(tidyr)
  # longer to wider
  # https://zhuanlan.zhihu.com/p/380003179
  tidyr:::spread.data.frame(dataExpr, key, value,
         fill = NA, convert = FALSE,
         drop = TRUE, sep = NULL)
  data_width <- spread(dataExpr,
                        key = "sample",
                        value = "NPC2")

  ###数据校正,均一化?
  library(limma)
  dataExpr=normalizeBetweenArrays(dataExpr)
  boxplot(dataExpr,outline=FALSE, notch=T,col=group_list, las=2)

  #查看数据范围，进行log转化缩小差距
  range(dataExpr)

  #log2逆转换
  #dataExpr=2^dataExpr
  #差距不大就不用log转换
  #dataExpr <- log2(dataExpr+1)
}

range(dataExpr)
dev.off()
write.csv(dataExpr,"GSE综合效应.csv")

####字符替换####
library(openxlsx)
library(data.table)
```

```
bc <- read.xlsx("GSEWhole for ROC.xlsx")
setnames (bc,
          old=c ("Group"),
          new=c ("default"))

####基于多重逻辑回归DCA####
getwd()
#https://mp.weixin.qq.com/s/dcN1BvmuSO7osWFPPq3pYg
#使用caret包中的createDataPartition()函数来创建内部训练集和验证集。
#install.packages('ggDCA')
#remotes::install_github('yikeshu0611/ggDCA')
library(caret)
library(ggDCA)
library(openxlsx)
LIRI <- read.xlsx("GSEWhole for ROC.xlsx")

#查看R包内置数据View(LIRI)
#制造内部训练集和验证集
train_id=createDataPartition(y = LIRI$Group,p = 0.7,list = FALSE)
train_data=LIRI[train_id,]
test_data=LIRI[-train_id,]

library(rms)
m1 <- lrm(Group~GBP5,train_data)
m2 <- lrm(Group~LHFPL2,train_data)
m3 <- lrm(Group~NPC2,train_data)
m4 <- lrm(Group~GBP5+LHFPL2,train_data)
m5 <- lrm(Group~LHFPL2+NPC2,train_data)
m6 <- lrm(Group~NPC2+GBP5,train_data)
m7 <- lrm(Group~NPC2+GBP5+LHFPL2,train_data)

#不改名绘制
m_train <- dca(m1,m2,m3,m4,m5,m6,m7)
ggplot(m_train)



#改名绘制
m_train <- dca(m1,m2,m3,m4,m5,m6,m7,
               model.names =c('GBP5',
                              'LHFPL2',
                              'NPC2',
                              'GBP5+LHFPL2',
                              'LHFPL2+NPC2',
                              'NPC2+GBP5',
                              'NPC2+GBP5+LHFPL2'))
ggplot(m_train)


#内部验证集
m_test <- dca(m1,m2,m3,m4,m5,m6,m7,
              new.data=test_data,
              model.names =c('GBP5',
                             'LHFPL2',
                             'NPC2',
                             'GBP5+LHFPL2',
                             'LHFPL2+NPC2',
                             'NPC2+GBP5',
                             'NPC2+GBP5+LHFPL2'))
ggplot(m_test)
#计算各项指标平均值
Result1 <- as.data.frame(aggregate(m_test[,1:4],list(m_test[,5]),mean))

##还需要引入外部验证集
```

```
####基于随机森林DCA####
library(randomForest)
library(pROC)
library(foreign)
library(caret)
library(ggDCA)
library(openxlsx)
bc <- read.xlsx("GSEWhole for ROC.xlsx")
#删除第一纵列
bc <- bc[,-(1:1)]
setnames (bc,
          old=c ("Group"),
          new=c ("default"))
bc$default<-as.factor(bc$default)

###设置训练和预测集
set.seed(1)
index <- sample(2,nrow(bc),replace = TRUE,prob=c(0.7,0.3))
traindata <- bc[index==1,]
testdata <- bc[index==2,]
###拟合随机森林模型，默认的mtry的值是自变量除以3
def_ntree<- randomForest(default~GBP5,data=traindata,
                         ntree=500,important=TRUE,proximity=TRUE)
def_pred<-predict(def_ntree,newdata=testdata,type = "prob")##生成概率
def_pred<-as.data.frame(def_pred)
testdata$def_pred<-def_pred$`1`
ggplot(def_pred)

#生成验证集人数和算出实际发生结果的发生率
N=dim(testdata)[1]
testdata$default<-as.numeric(testdata$default)-1
event.rate=mean(testdata$default)

#未发生率
1-event.rate

#建一个净获益的预测数据表
nb=data.frame(threshold=seq(from=0, to=1, by=0.01))
#根据净获益公式可以得到如下
nb["all"]=event.rate- (1-event.rate)*nb$threshold/(1-nb$threshold)
nb["none"]=0
#算出真阳性和假阳性的人数（tp和fp），
#因为需要算的是不同预测下的真阳性和假阳性的人数（tp和fp），
#所以每个阈值都要跑一下，这里要写一个循环
for(t in 1:length(nb$threshold)){
  tp=mean(testdata[testdata["def_pred"]>=nb$threshold[t],
                   "default"])*sum(testdata["def_pred"]>=nb$threshold[t])
  fp=(1-mean(testdata[testdata["def_pred"]>=nb$threshold[t],
                      "default"]))*sum(testdata["def_pred"]>=nb$threshold[t])
  if(sum(testdata["def_pred"]>=nb$threshold[t])==0) {
    tp=0
    fp=0
  }
  nb[t,"def_pred"]=tp/N- fp/N*(nb$threshold[t]/(1-nb$threshold[t]))  #净获益公式
}

#计算各项指标平均值
Result1 <- as.data.frame(aggregate(m_test[,1:4],list(m_test[,5]),mean))

#结果合并
Result <- bind_rows(list(Result1,
                         Result2),.id = "id")
if(!dir.exists("DCA分析结果")){dir.create("DCA分析结果")}
write.csv(m_test,file = "DCA分析结果/Whole.csv")
```

```
####SVM2####
#data("iris")
library(tidyverse)
library(caret)
library(openxlsx)
library(data.table)
library(mlbench)
#示例数据
#data('PimaIndiansDiabetes2',package='mlbench')
data <- read.xlsx("GSEWhole for ROC.xlsx")
#删除第一纵列
data <- data[,-(1:1)]
library(ggplot2)
qplot(GBP5,LHFPL2,data = data, color = Group)
#??ggplot2

library(e1071)
#核函数类型有四种，判断一下那个核函数判断的正确率最高
#1.基于径向基核函数radial
#2.线性核函数linear
#3.多项式核函数polynomial
#4.神经网络核函数sigmoid
mymodel <- svm(Group~.,data=data,kernel="radial")
pred <- predict(mymodel,data)
#建立混淆矩阵
tab <- table(Predicted=pred,Actual=data$Group)
tab
radial <- sum(diag(tab))/sum(tab)

mymodel <- svm(Group~.,data=data,kernel="linear")
pred <- predict(mymodel,data)
tab <- table(Predicted=pred,Actual=data$Group)
tab
linear <- sum(diag(tab))/sum(tab)

mymodel <- svm(Group~.,data=data,kernel="polynomial")
pred <- predict(mymodel,data)
tab <- table(Predicted=pred,Actual=data$Group)
tab
polynomial <- sum(diag(tab))/sum(tab)

mymodel <- svm(Group~.,data=data,kernel="sigmoid")
pred <- predict(mymodel,data)
tab <- table(Predicted=pred,Actual=data$Group)
tab
sigmoid <- sum(diag(tab))/sum(tab)

#最终给出准确率,选择最佳核函数
#使用tune函数进行逆行调整，模型的调整(超参数优化)
set.seed(124)
tmodel <- tune(svm,Group~.,data=data,
               ranges=list(epslon=seq(0,1,0.1,),
                           cost=2^(2:7)))
plot(tmodel)
#参数对应较暗的区域意味着更好的结果在这些区域
#根据图的结果可适当扩大或缩小cost的范围
summary(tmodel)

#最佳模型
mymodel <- tmodel$best.model
summary(mymodel)
pred <- predict(mymodel,data)
tab <- table(Predicted=pred,Actual=data$Group)
tab
best_mymodel <- sum(diag(tab))/sum(tab)
```

```
print(mymodel)

####XGBoost####
#https://mp.weixin.qq.com/s/FgPsjaVhxYrgysU-mrPRRA
library(xgboost)
library(caret)
library(ggDCA)
library(openxlsx)
bc <- read.xlsx("GSEWhole for ROC.xlsx")
if(!dir.exists("XGBoost分析结果")){dir.create("XGBoost分析结果")}

bc<-bc[,c("Group","GBP5","LHFPL2","NPC2")]
bc$Group<-as.factor(bc$Group)
x =model.matrix(Group~.,bc)[,-1]
data_train<- xgb.DMatrix(x , label =as.numeric(bc$Group))

param<- list(objective = "reg:squarederror")
HR_xgb_model<- xgb.train(param, data_train, nrounds = 50)
HR_xgb_model

library("DALEX")
predict_logit<- function(model, x) {
  raw_x <- predict(model, x)
  exp(raw_x)/(1 + exp(raw_x))
}
logit<- function(x) {
  exp(x)/(1+exp(x))
}

explainer_xgb<- explain(HR_xgb_model,
                        data = x,
                        y =as.numeric(bc$Group),
                        predict_function = predict_logit,
                        link = logit,
                        label ="xgboost")
explainer_xgb


sv_xgb_satisfaction_level<-DALEX::model_profile(explainer_xgb,
                                                type = "partial")
head(sv_xgb_satisfaction_level)
plot(sv_xgb_satisfaction_level)

library("breakDown")
library(ggplot2)
nobs<-model_martix_train[1L, , drop = FALSE]
explain_2<- broken(HR_xgb_model, new_observation = nobs,
                   data = model_martix_train)
explain_2

write.csv(m_train,file = "MLR分析结果/训练集-whole.csv")
write.csv(m_test,file = "MLR分析结果/验证集-whole.csv")

####惩罚回归####
getwd()
#加载包
{
library(tidyverse)
library(caret)
library(glmnet)
library(openxlsx)
library(data.table)
}
###数据准备
#加载数据
```

```
#示例数据
#data('Boston',package='MASS')
data <- read.csv("GSE37250_exp.csv")
rownames(data) <- data$X
data <- data[,-1]
data <- t(data)
data <- as.data.frame(data)
group <- read.csv("GSE37250_group.csv")
rownames(group) <- group$geo_accession
#分组信息需要手动处理
group <- group[,-2]
group <- group[,-2]
group <- group[,-2]
group <- group[,-(2:7)]
group <- group[,-(3:30)]
group <- group[,-(3:24)]
#整合数据集
{
  LASSO <- cbind(group,data)
  LASSO <- LASSO[,-1]
  library(dplyr)
  group <- group %>% filter(grepl('ATB|LTBI',group))
  LASSO_1 <- LASSO %>% filter(grepl('ATB|LTBI',group))
  LASSO_1[LASSO_1=="ATB"]<-1
  LASSO_1[LASSO_1=="LTBI"]<-0
  data <- LASSO_1
  data[,c(1)] <- as.numeric(unlist(data[,c(1)]))
  Boston <- data
}
#Boston <- read.xlsx("GSEWhole for ROC.xlsx")
#数据处理
{
#数据格式同lasso
setnames (Boston,
          old=c ("group"),
          new=c ("medv"))
#挑选目的3基因
Boston<- Boston[,grepl("medv|GPR84|S100A12|S100A8",
                       colnames(Boston))]
#分为训练集和验证集
set.seed(123)
trainning.sample <- Boston$medv %>% createDataPartition(p=0.8,list=FALSE)
train.data <- Boston[trainning.sample,]
test.data <- Boston[-trainning.sample,]
y <- train.data$medv
}

####计算岭回归####
#首次的第一个模型：12
{x <- model.matrix(medv~GPR84+S100A12, train.data)[,-1]
#确定lambda取值
set.seed(123)
cv <- cv.glmnet(x, y, alpha = 0)
cv$lambda.min
# 计算岭回归
model <- glmnet(x, y, alpha = 0, lambda =cv$lambda.min)
coef(model)
#选择用于预测的数据矩阵
x.test <- model.matrix(medv ~., test.data%>% select(1,2,3))[,-1]
#验证集分类的预测
predictions <- model %>% predict(x.test)
library(ROCR)
#模型1的AUC质量评估
#使用AUC评估模型质量
#使用predict方法得到全模型对原始数据的预测概率probFull,
```

```
#然后用prediction方法将预测概率转化为标准化
#https://zhuanlan.zhihu.com/p/342089730
probFull <- predict(model, x.test, type = "response")
predictFull <- prediction(probFull, test.data$medv)
#perfFull <- performance(predictFull, measure = "tpr", x.measure = "fpr")
#plot(perfFull)
#plot(perfFull, col = "blue")
#AUC1 <- performance(predictFull, measure = "auc")@y.values
#sens_spec_1 <- performance(predictFull, measure="sens", x.measure="spec")
#plot(sens_spec_1)
#AUC1 <- AUC1[[1]]
##模型的ROC评估
{#模型的数值
  value <- as.data.frame(predictFull@predictions[[1]])
  #验证集的分组
  group <- as.data.frame(test.data$medv)
  #整合数据集供ROC检验
  df <- cbind(group,value)
  library(dplyr)
  df <- tibble::rownames_to_column(df,"id")
  names(df) <- c("ID","Group","Model")
  library(multipleROC)
  df <- as.data.frame(df)
  p <- multipleROC(Group~Model,data=df)
  plot_ROC(p,
           show.points = T,
           show.eta = T,
           show.sens = T,
           show.AUC = T,
           facet = F )
}
ROC统计结果<- data.frame("参数"=c("cutpoint","sens","auc"),
                    "Model"=c(p[["cutpoint"]],
                              p[["sens"]],
                              p[["spec"]],
                              p[["auc"]]))
ROC统计结果1<-ROC统计结果
}

#第一个模型：12
{x <- model.matrix(medv~GPR84+S100A12, train.data)[,-1]
  #确定lambda取值
  set.seed(123)
  cv <- cv.glmnet(x, y, alpha = 0)
  cv$lambda.min
  # 计算岭回归
  model <- glmnet(x, y, alpha = 0, lambda =cv$lambda.min)
  coef(model)
  #选择用于预测的数据矩阵
  x.test <- model.matrix(medv ~., test.data%>% select(1,2,3))[,-1]
  #验证集分类的预测
  predictions <- model %>% predict(x.test)
  library(ROCR)
  #模型1的AUC质量评估
  #使用AUC评估模型质量
  #使用predict方法得到全模型对原始数据的预测概率probFull,
  #然后用prediction方法将预测概率转化为标准化
  #https://zhuanlan.zhihu.com/p/342089730
  probFull <- predict(model, x.test, type = "response")
  predictFull <- prediction(probFull, test.data$medv)
  #perfFull <- performance(predictFull, measure = "tpr", x.measure = "fpr")
  #plot(perfFull)
  #plot(perfFull, col = "blue")
  #AUC1 <- performance(predictFull, measure = "auc")@y.values
  #sens_spec_1 <- performance(predictFull, measure="sens", x.measure="spec")
```

```
  #plot(sens_spec_1)
  #AUC1 <- AUC1[[1]]
  ##模型的ROC评估
  {#模型的数值
    value <- as.data.frame(predictFull@predictions[[1]])
    #验证集的分组
    group <- as.data.frame(test.data$medv)
    #整合数据集供ROC检验
    df <- cbind(group,value)
    library(dplyr)
    df <- tibble::rownames_to_column(df,"id")
    names(df) <- c("ID","Group","Model")
    library(multipleROC)
    df <- as.data.frame(df)
    p <- multipleROC(Group~Model,data=df)
    plot_ROC(p,
             show.points = T,
             show.eta = T,
             show.sens = T,
             show.AUC = T,
             facet = F )
  }
  ROC统计结果<- data.frame("参数"=c("cutpoint","sens","auc"),
                       "Model"=c(p[["cutpoint"]],
                                 p[["sens"]],
                                 p[["spec"]],
                                 p[["auc"]]))
  ROC统计结果1<- merge(ROC统计结果1,ROC统计结果,by = "参数")
}
#第二个模型：13
{x <- model.matrix(medv~GPR84+S100A8, train.data)[,-1]
  set.seed(123)
  cv <- cv.glmnet(x, y, alpha = 0)
  cv$lambda.min
  model <- glmnet(x, y, alpha = 0, lambda =cv$lambda.min)
  coef(model)
  x.test <- model.matrix(medv ~., test.data%>% select(1,2,4))[,-1]
  predictions <- model %>% predict(x.test)
  probFull <- predict(model, x.test, type = "response")
  predictFull <- prediction(probFull, test.data$medv)
  #perfFull <- performance(predictFull, measure = "tpr", x.measure = "fpr")
  #plot(perfFull, col = "blue",add = T)
  #AUC2 <- performance(predictFull, measure = "auc")@y.values
  #AUC2 <- AUC2[[1]]
  ##模型的ROC评估
  {#模型的数值
    value <- as.data.frame(predictFull@predictions[[1]])
    #验证集的分组
    group <- as.data.frame(test.data$medv)
    #整合数据集供ROC检验
    df <- cbind(group,value)
    library(dplyr)
    df <- tibble::rownames_to_column(df,"id")
    names(df) <- c("ID","Group","Model")
    library(multipleROC)
    df <- as.data.frame(df)
    p <- multipleROC(Group~Model,data=df)
    plot_ROC(p,
             show.points = T,
             show.eta = T,
             show.sens = T,
             show.AUC = T,
             facet = F )
  }
  ROC统计结果<- data.frame("参数"=c("cutpoint","sens","auc"),
```

```
                    "Model"=c(p[["cutpoint"]],
                              p[["sens"]],
                              p[["spec"]],
                              p[["auc"]]))
  ROC统计结果1<- merge(ROC统计结果1,ROC统计结果,by = "参数")
}
#第三个模型：23
{x <- model.matrix(medv~S100A12+S100A8, train.data)[,-1]
  set.seed(123)
  cv <- cv.glmnet(x, y, alpha = 0)
  cv$lambda.min
  model <- glmnet(x, y, alpha = 0, lambda =cv$lambda.min)
  coef(model)
  x.test <- model.matrix(medv ~., test.data%>% select(1,3,4))[,-1]
  predictions <- model %>% predict(x.test)
  probFull <- predict(model, x.test, type = "response")
  predictFull <- prediction(probFull, test.data$medv)
  #perfFull <- performance(predictFull, measure = "tpr", x.measure = "fpr")
  #plot(perfFull, col = "blue",add = T)
  #AUC3 <- performance(predictFull, measure = "auc")@y.values
  #AUC3 <- AUC3[[1]]
  ##模型的ROC评估
  {#模型的数值
    value <- as.data.frame(predictFull@predictions[[1]])
    #验证集的分组
    group <- as.data.frame(test.data$medv)
    #整合数据集供ROC检验
    df <- cbind(group,value)
    library(dplyr)
    df <- tibble::rownames_to_column(df,"id")
    names(df) <- c("ID","Group","Model")
    library(multipleROC)
    df <- as.data.frame(df)
    p <- multipleROC(Group~Model,data=df)
    plot_ROC(p,
             show.points = T,
             show.eta = T,
             show.sens = T,
             show.AUC = T,
             facet = F )
  }
  ROC统计结果<- data.frame("参数"=c("cutpoint","sens","auc"),
                    "Model"=c(p[["cutpoint"]],
                              p[["sens"]],
                              p[["spec"]],
                              p[["auc"]]))
  ROC统计结果1<- merge(ROC统计结果1,ROC统计结果,by = "参数")
}
#第四个模型：123
{x <- model.matrix(medv~., train.data)[,-1]
  set.seed(123)
  cv <- cv.glmnet(x, y, alpha = 0)
  cv$lambda.min
  model <- glmnet(x, y, alpha = 0, lambda =cv$lambda.min)
  coef(model)
  x.test <- model.matrix(medv ~., test.data%>% select(1:4))[,-1]
  predictions <- model %>% predict(x.test)
  probFull <- predict(model, x.test, type = "response")
  predictFull <- prediction(probFull, test.data$medv)
  #perfFull <- performance(predictFull, measure = "tpr", x.measure = "fpr")
  #plot(perfFull, col = "blue",add = T)
  #AUC4 <- performance(predictFull, measure = "auc")@y.values
  #AUC4 <- AUC4[[1]]
  ##模型的ROC评估
  {#模型的数值
```

```r
    value <- as.data.frame(predictFull@predictions[[1]])
    #验证集的分组
    group <- as.data.frame(test.data$medv)
    #整合数据集供ROC检验
    df <- cbind(group,value)
    library(dplyr)
    df <- tibble::rownames_to_column(df,"id")
    names(df) <- c("ID","Group","Model")
    library(multipleROC)
    df <- as.data.frame(df)
    p <- multipleROC(Group~Model,data=df)
    plot_ROC(p,
             show.points = T,
             show.eta = T,
             show.sens = T,
             show.AUC = T,
             facet = F )
  }
  ROC统计结果<- data.frame("参数"=c("cutpoint","sens","auc"),
                      "Model"=c(p[["cutpoint"]],
                                p[["sens"]],
                                p[["spec"]],
                                p[["auc"]]))
  ROC统计结果1<- merge(ROC统计结果1,ROC统计结果,by = "参数")
}

#数据整理，更改列名
r<- ROC统计结果1
rownames(r) <- r$参数
r <- r[,-(1)]
#列名改为数字
colnames(r) <- 1:32
original_cols <- colnames(r)
colnames(r) <- paste("Model" ,"No",original_cols,sep="_")
class(r)
#储存结果
if(!dir.exists("Ridge prediction result")){dir.create("Ridge prediction result")}
write.csv(r,"Ridge prediction result/Ridge.csv")

####写入exls不同分页####
library(rJava)
library(xlsxjars)
library(xlsx)
if(!dir.exists("Ridge prediction result")){dir.create("Ridge prediction result")}
write.xlsx(Ridge_prediction_1, file="Ridge prediction result/Ridge9.xlsx",
           sheetName="12",
           row.names=FALSE)
write.xlsx(Ridge_prediction_2, file="Ridge prediction result/Ridge9.xlsx",
           sheetName="13",
           append=TRUE,
           row.names=FALSE)     #append用于追加不同sheet
write.xlsx(Ridge_prediction_3, file="Ridge prediction result/Ridge9.xlsx",
           sheetName="23",
           append=TRUE,
           row.names=FALSE)     #append用于追加不同sheet
write.xlsx(Ridge_prediction_4, file="Ridge prediction result/Ridge9.xlsx",
           sheetName="123",
           append=TRUE,
           row.names=FALSE)     #append用于追加不同sheet
#因为写入xlsx的与读入的有冲突，所以要清楚加载的包
library(pacman)
pacman::p_unload(pacman::p_loaded(), character.only = TRUE)

#平均精准度（此处应有误）
#Mean_Accuracy_1 <- mean(predictions)
```

```
#predictions <- model %>% predict(x.test) %>% as.vector()
#模型性能
Result1 <- data.frame(
  row.names = "Ridge regression",
  RMSE = RMSE(predictions, test.data$medv),
  Rsquare = R2(predictions, test.data$medv),
  Mean_Accuracy = Mean_Accuracy_1
)


####计算LASSO回归####
library(tidyverse)
library(caret)
library(glmnet)
library(openxlsx)
library(data.table)
###数据准备
#加载数据
#示例数据
#data('Boston',package='MASS')
data <- read.csv("GSE40553_exp.csv")
rownames(data) <- data$X
data <- data[,-1]
data <- t(data)
data <- as.data.frame(data)
group <- read.csv("GSE40553_group.csv")
rownames(group) <- group$geo_accession
#分组信息需要手动处理
group <- group[,-2]
group <- group[,-2]
group <- group[,-2]
group <- group[,-(2:7)]
group <- group[,-(2:4)]
group <- group[,-(3:30)]
group <- group[,-(3:24)]
#整合数据集
{
  LASSO <- cbind(group,data)
  LASSO <- LASSO[,-1]
  library(dplyr)
  group <- group %>% filter(grepl('ATB|LTBI',group))
  LASSO_1 <- LASSO %>% filter(grepl('ATB|LTBI',group))
  LASSO_1[LASSO_1=="ATB"]<-1
  LASSO_1[LASSO_1=="LTBI"]<-0
  data <- LASSO_1
  data[,c(1)] <- as.numeric(unlist(data[,c(1)]))
  Boston <- data
}
#Boston <- read.xlsx("GSEWhole for ROC.xlsx")
#数据处理
{
  #数据格式同lasso
  setnames (Boston,
            old=c ("group"),
            new=c ("medv"))
  #挑选目的3基因
  Boston<- Boston[,grepl("medv|GPR84|S100A12|S100A8",
                         colnames(Boston))]
  #分为训练集和验证集
  set.seed(123)
  trainning.sample <- Boston$medv %>% createDataPartition(p=0.8,list=FALSE)
  train.data <- Boston[trainning.sample,]
  test.data <- Boston[-trainning.sample,]
  y <- train.data$medv
}
```

```
#首次的第一个模型：12
{
  x <- model.matrix(medv~GPR84+S100A12, train.data)[,-1]
  #确定lambda取值
  set.seed(123)
  cv <- cv.glmnet(x, y, alpha = 1)
  cv$lambda.min
  # 计算岭回归
  model <- glmnet(x, y, alpha = 1, lambda =cv$lambda.min)
  coef(model)
  #选择用于预测的数据矩阵
  x.test <- model.matrix(medv ~., test.data%>% select(1,2,3))[,-1]
  #验证集分类的预测
  predictions <- model %>% predict(x.test)
  library(ROCR)
  probFull <- predict(model, x.test, type = "response")
  predictFull <- prediction(probFull, test.data$medv)
  ##模型的ROC评估
  {#模型的数值
    value <- as.data.frame(predictFull@predictions[[1]])
    #验证集的分组
    group <- as.data.frame(test.data$medv)
    #整合数据集供ROC检验
    df <- cbind(group,value)
    library(dplyr)
    df <- tibble::rownames_to_column(df,"id")
    names(df) <- c("ID","Group","Model")
    library(multipleROC)
    df <- as.data.frame(df)
    p <- multipleROC(Group~Model,data=df)
    plot_ROC(p,
             show.points = T,
             show.eta = T,
             show.sens = T,
             show.AUC = T,
             facet = F )
  }
  ROC统计结果<- data.frame("参数"=c("cutpoint","sens","auc"),
                      "Model"=c(p[["cutpoint"]],
                                p[["sens"]],
                                p[["spec"]],
                                p[["auc"]]))
  ROC统计结果1<-ROC统计结果
}

#第一个模型：12
{
  x <- model.matrix(medv~GPR84+S100A12, train.data)[,-1]
  #确定lambda取值
  set.seed(123)
  cv <- cv.glmnet(x, y, alpha = 1)
  cv$lambda.min
  # 计算岭回归
  model <- glmnet(x, y, alpha = 1, lambda =cv$lambda.min)
  coef(model)
  #选择用于预测的数据矩阵
  x.test <- model.matrix(medv ~., test.data%>% select(1,2,3))[,-1]
  #验证集分类的预测
  predictions <- model %>% predict(x.test)
  library(ROCR)
  probFull <- predict(model, x.test, type = "response")
  predictFull <- prediction(probFull, test.data$medv)
  ##模型的ROC评估
  {#模型的数值
```

```
    value <- as.data.frame(predictFull@predictions[[1]])
    #验证集的分组
    group <- as.data.frame(test.data$medv)
    #整合数据集供ROC检验
    df <- cbind(group,value)
    library(dplyr)
    df <- tibble::rownames_to_column(df,"id")
    names(df) <- c("ID","Group","Model")
    library(multipleROC)
    df <- as.data.frame(df)
    p <- multipleROC(Group~Model,data=df)
    plot_ROC(p,
             show.points = T,
             show.eta = T,
             show.sens = T,
             show.AUC = T,
             facet = F )
  }
  ROC统计结果<- data.frame("参数"=c("cutpoint","sens","auc"),
                      "Model"=c(p[["cutpoint"]],
                                p[["sens"]],
                                p[["spec"]],
                                p[["auc"]]))
  #ROC统计结果1<-ROC统计结果
  ROC统计结果1<- merge(ROC统计结果1,ROC统计结果,by = "参数")
}
#第二个模型：13
{x <- model.matrix(medv~GPR84+S100A8, train.data)[,-1]
  set.seed(123)
  cv <- cv.glmnet(x, y, alpha = 1)
  cv$lambda.min
  model <- glmnet(x, y, alpha = 1, lambda =cv$lambda.min)
  coef(model)
  x.test <- model.matrix(medv ~., test.data%>% select(1,2,4))[,-1]
  predictions <- model %>% predict(x.test)
  probFull <- predict(model, x.test, type = "response")
  predictFull <- prediction(probFull, test.data$medv)
  ##模型的ROC评估
  {#模型的数值
    value <- as.data.frame(predictFull@predictions[[1]])
    #验证集的分组
    group <- as.data.frame(test.data$medv)
    #整合数据集供ROC检验
    df <- cbind(group,value)
    library(dplyr)
    df <- tibble::rownames_to_column(df,"id")
    names(df) <- c("ID","Group","Model")
    library(multipleROC)
    df <- as.data.frame(df)
    p <- multipleROC(Group~Model,data=df)
    plot_ROC(p,
             show.points = T,
             show.eta = T,
             show.sens = T,
             show.AUC = T,
             facet = F )
  }
  ROC统计结果<- data.frame("参数"=c("cutpoint","sens","auc"),
                      "Model"=c(p[["cutpoint"]],
                                p[["sens"]],
                                p[["spec"]],
                                p[["auc"]]))
  #ROC统计结果1<-ROC统计结果
  ROC统计结果1<- merge(ROC统计结果1,ROC统计结果,by = "参数")
}
```

```
#第三个模型：23
{x <- model.matrix(medv~S100A12+S100A8, train.data)[,-1]
  set.seed(123)
  cv <- cv.glmnet(x, y, alpha = 1)
  cv$lambda.min
  model <- glmnet(x, y, alpha = 1, lambda =cv$lambda.min)
  coef(model)
  x.test <- model.matrix(medv ~., test.data%>% select(1,3,4))[,-1]
  predictions <- model %>% predict(x.test)
  probFull <- predict(model, x.test, type = "response")
  predictFull <- prediction(probFull, test.data$medv)
  ##模型的ROC评估
  {#模型的数值
    value <- as.data.frame(predictFull@predictions[[1]])
    #验证集的分组
    group <- as.data.frame(test.data$medv)
    #整合数据集供ROC检验
    df <- cbind(group,value)
    library(dplyr)
    df <- tibble::rownames_to_column(df,"id")
    names(df) <- c("ID","Group","Model")
    library(multipleROC)
    df <- as.data.frame(df)
    p <- multipleROC(Group~Model,data=df)
    plot_ROC(p,
             show.points = T,
             show.eta = T,
             show.sens = T,
             show.AUC = T,
             facet = F )
  }
  ROC统计结果<- data.frame("参数"=c("cutpoint","sens","auc"),
                      "Model"=c(p[["cutpoint"]],
                                p[["sens"]],
                                p[["spec"]],
                                p[["auc"]]))
  #ROC统计结果1<-ROC统计结果
  ROC统计结果1<- merge(ROC统计结果1,ROC统计结果,by = "参数")
}
#第四个模型：123
{x <- model.matrix(medv~., train.data)[,-1]
  set.seed(123)
  cv <- cv.glmnet(x, y, alpha = 1)
  cv$lambda.min
  model <- glmnet(x, y, alpha = 1, lambda =cv$lambda.min)
  coef(model)
  x.test <- model.matrix(medv ~., test.data%>% select(1:4))[,-1]
  predictions <- model %>% predict(x.test)
  probFull <- predict(model, x.test, type = "response")
  predictFull <- prediction(probFull, test.data$medv)
  ##模型的ROC评估
  {#模型的数值
    value <- as.data.frame(predictFull@predictions[[1]])
    #验证集的分组
    group <- as.data.frame(test.data$medv)
    #整合数据集供ROC检验
    df <- cbind(group,value)
    library(dplyr)
    df <- tibble::rownames_to_column(df,"id")
    names(df) <- c("ID","Group","Model")
    library(multipleROC)
    df <- as.data.frame(df)
    p <- multipleROC(Group~Model,data=df)
    plot_ROC(p,
             show.points = T,
```

```
            show.eta = T,
            show.sens = T,
            show.AUC = T,
            facet = F )
  }
  ROC统计结果<- data.frame("参数"=c("cutpoint","sens","auc"),
                      "Model"=c(p[["cutpoint"]],
                                p[["sens"]],
                                p[["spec"]],
                                p[["auc"]]))
  #ROC统计结果1<-ROC统计结果
  ROC统计结果1<- merge(ROC统计结果1,ROC统计结果,by = "参数")
}

##模型的ROC评估
{#模型的数值
  value <- as.data.frame(predictFull@predictions[[1]])
  #验证集的分组
  group <- as.data.frame(test.data$medv)
  #整合数据集供ROC检验
  df <- cbind(group,value)
  library(dplyr)
  df <- tibble::rownames_to_column(df,"id")
  names(df) <- c("ID","Group","Model")
  library(multipleROC)
  df <- as.data.frame(df)
  p <- multipleROC(Group~Model,data=df)
  plot_ROC(p,
          show.points = T,
          show.eta = T,
          show.sens = T,
          show.AUC = T,
          facet = F )
}
ROC统计结果<- data.frame("参数"=c("cutpoint","sens","auc"),
                    "Model"=c(p[["cutpoint"]],
                              p[["sens"]],
                              p[["spec"]],
                              p[["auc"]]))
#ROC统计结果1<-ROC统计结果
ROC统计结果1<- merge(ROC统计结果1,ROC统计结果,by = "参数")

#数据整理，更改列名
{
r<- ROC统计结果1
rownames(r) <- r$参数
r <- r[,-(1)]
#列名改为数字
colnames(r) <- 1:32
original_cols <- colnames(r)
colnames(r) <- paste("Model" ,"No",original_cols,sep="_")
class(r)
#储存结果
if(!dir.exists("LASSO prediction result")){dir.create("LASSO prediction result")}
write.csv(r,"LASSO prediction result/LASSO.csv")
}
####计算弹性网络回归####
#Elastic-Net Regression
library(tidyverse)
library(caret)
library(glmnet)
library(openxlsx)
library(data.table)
###数据准备
#加载数据
```

```
#示例数据
#data('Boston',package='MASS')
data <- read.csv("GSE40553_exp.csv")
rownames(data) <- data$X
data <- data[,-1]
data <- t(data)
data <- as.data.frame(data)
group <- read.csv("GSE40553_group.csv")
rownames(group) <- group$geo_accession
#分组信息需要手动处理
group <- group[,-2]
group <- group[,-2]
group <- group[,-2]
group <- group[,-(2:7)]
group <- group[,-(2:4)]
group <- group[,-(3:30)]
group <- group[,-(3:24)]
#整合数据集
{
  LASSO <- cbind(group,data)
  LASSO <- LASSO[,-1]
  library(dplyr)
  group <- group %>% filter(grepl('ATB|LTBI',group))
  LASSO_1 <- LASSO %>% filter(grepl('ATB|LTBI',group))
  LASSO_1[LASSO_1=="ATB"]<-1
  LASSO_1[LASSO_1=="LTBI"]<-0
  data <- LASSO_1
  data[,c(1)] <- as.numeric(unlist(data[,c(1)]))
  Boston <- data
}
#Boston <- read.xlsx("GSEWhole for ROC.xlsx")
#数据处理
{
  #数据格式同lasso
  setnames (Boston,
            old=c ("group"),
            new=c ("medv"))
  #挑选目的3基因
  Boston<- Boston[,grepl("medv|GPR84|S100A12|S100A8",
                         colnames(Boston))]
  #按顺序排列
  target <- c("medv","GPR84","S100A12","S100A8")
  # 然后将 dataframe 按照新的列名顺序排列
  Boston <- Boston[,target]
  #分为训练集和验证集
  set.seed(123)
  trainning.sample <- Boston$medv %>% createDataPartition(p=0.8,list=FALSE)
  train.data <- Boston[trainning.sample,]
  test.data <- Boston[-trainning.sample,]
  y <- train.data$medv
}

#首次的模型1-12
{
  x <- model.matrix(medv~GPR84+S100A12, train.data)[,-1]
  set.seed(123)
  model <- train(
    medv~GPR84+S100A12,train.data %>% select(1,2,3), method = 'glmnet',
    trControl = trainControl('cv',number = 10),
    tuneLength = 10
  )
  model$bestTune
  coef(model$finalModel,model$bestTune$lambda)
  #数据预测
  x.test <- model.matrix(medv~.,
```

```
                      test.data %>% select(1,2,3))[,-1]
  #验证集分类的预测
  predictions <- model %>% predict(x.test)
  library(ROCR)
  probFull <- predict(model, x.test, type = "raw")
  predictFull <- prediction(probFull, test.data$medv)
  ##模型的ROC评估
  {#模型的数值
    value <- as.data.frame(predictFull@predictions[[1]])
    #验证集的分组
    group <- as.data.frame(test.data$medv)
    #整合数据集供ROC检验
    df <- cbind(group,value)
    library(dplyr)
    df <- tibble::rownames_to_column(df,"id")
    names(df) <- c("ID","Group","Model")
    library(multipleROC)
    df <- as.data.frame(df)
    p <- multipleROC(Group~Model,data=df)
    plot_ROC(p,
             show.points = T,
             show.eta = T,
             show.sens = T,
             show.AUC = T,
             facet = F )
  }
  ROC统计结果<- data.frame("参数"=c("cutpoint","sens","auc"),
                     "Model"=c(p[["cutpoint"]],
                              p[["sens"]],
                              p[["spec"]],
                              p[["auc"]]))
  ROC统计结果1<-ROC统计结果
}

#四种模型的计算
{
#模型1-12
{
  x <- model.matrix(medv~GPR84+S100A12, train.data)[,-1]
  set.seed(123)
  model <- train(
    medv~GPR84+S100A12,train.data %>% select(1,2,3), method = 'glmnet',
    trControl = trainControl('cv',number = 10),
    tuneLength = 10
  )
  model$bestTune
  coef(model$finalModel,model$bestTune$lambda)
  #数据预测
  x.test <- model.matrix(medv~GPR84+S100A12,
                      test.data %>% select(1,2,3))[,-1]
  #验证集分类的预测
  predictions <- model %>% predict(x.test)
  library(ROCR)
  probFull <- predict(model, x.test, type = "raw")
  predictFull <- prediction(probFull, test.data$medv)
  ##模型的ROC评估
  {#模型的数值
    value <- as.data.frame(predictFull@predictions[[1]])
    #验证集的分组
    group <- as.data.frame(test.data$medv)
    #整合数据集供ROC检验
    df <- cbind(group,value)
    library(dplyr)
    df <- tibble::rownames_to_column(df,"id")
    names(df) <- c("ID","Group","Model")
```

```
    library(multipleROC)
    df <- as.data.frame(df)
    p <- multipleROC(Group~Model,data=df)
    plot_ROC(p,
             show.points = T,
             show.eta = T,
             show.sens = T,
             show.AUC = T,
             facet = F )
  }
  ROC统计结果<- data.frame("参数"=c("cutpoint","sens","auc"),
                       "Model"=c(p[["cutpoint"]],
                                 p[["sens"]],
                                 p[["spec"]],
                                 p[["auc"]]))
  #ROC统计结果1<-ROC统计结果
  ROC统计结果1<- merge(ROC统计结果1,ROC统计结果,by = "参数")
}
#模型2-13
{
  set.seed(123)
  model <- train(
    medv~GPR84+S100A8,train.data %>% select(1,2,4), method = 'glmnet',
    trControl = trainControl('cv',number = 10),
    tuneLength = 10
  )
  model$bestTune
  coef(model$finalModel,model$bestTune$lambda)
  #数据预测
  x.test <- model.matrix(medv~GPR84+S100A8, test.data %>% select(1,2,4))[,-1]
  #验证集分类的预测
  predictions <- model %>% predict(x.test)
  library(ROCR)
  probFull <- predict(model, x.test, type = "raw")
  predictFull <- prediction(probFull, test.data$medv)
  ##模型的ROC评估
  {#模型的数值
    value <- as.data.frame(predictFull@predictions[[1]])
    #验证集的分组
    group <- as.data.frame(test.data$medv)
    #整合数据集供ROC检验
    df <- cbind(group,value)
    library(dplyr)
    df <- tibble::rownames_to_column(df,"id")
    names(df) <- c("ID","Group","Model")
    library(multipleROC)
    df <- as.data.frame(df)
    p <- multipleROC(Group~Model,data=df)
    plot_ROC(p,
             show.points = T,
             show.eta = T,
             show.sens = T,
             show.AUC = T,
             facet = F )
  }
  ROC统计结果<- data.frame("参数"=c("cutpoint","sens","auc"),
                       "Model"=c(p[["cutpoint"]],
                                 p[["sens"]],
                                 p[["spec"]],
                                 p[["auc"]]))
  #ROC统计结果1<-ROC统计结果
  ROC统计结果1<- merge(ROC统计结果1,ROC统计结果,by = "参数")
}
#模型3-23
{
```

```
  set.seed(123)
  model <- train(
    medv~S100A12+S100A8,train.data %>% select(1,3,4), method = 'glmnet',
    trControl = trainControl('cv',number = 10),
    tuneLength = 10
  )
  model$bestTune
  coef(model$finalModel,model$bestTune$lambda)
  #数据预测
  x.test <- model.matrix(medv~S100A12+S100A8, test.data %>% select(1,3,4))[,-1]
  #验证集分类的预测
  predictions <- model %>% predict(x.test)
  library(ROCR)
  probFull <- predict(model, x.test, type = "raw")
  predictFull <- prediction(probFull, test.data$medv)
  ##模型的ROC评估
  {#模型的数值
    value <- as.data.frame(predictFull@predictions[[1]])
    #验证集的分组
    group <- as.data.frame(test.data$medv)
    #整合数据集供ROC检验
    df <- cbind(group,value)
    library(dplyr)
    df <- tibble::rownames_to_column(df,"id")
    names(df) <- c("ID","Group","Model")
    library(multipleROC)
    df <- as.data.frame(df)
    p <- multipleROC(Group~Model,data=df)
    plot_ROC(p,
             show.points = T,
             show.eta = T,
             show.sens = T,
             show.AUC = T,
             facet = F )
  }
  ROC统计结果<- data.frame("参数"=c("cutpoint","sens","auc"),
                      "Model"=c(p[["cutpoint"]],
                                p[["sens"]],
                                p[["spec"]],
                                p[["auc"]]))
  #ROC统计结果1<-ROC统计结果
  ROC统计结果1<- merge(ROC统计结果1,ROC统计结果,by = "参数")
}
#模型4-123
{
  set.seed(123)
  model <- train(
    medv~.,train.data, method = 'glmnet',
    trControl = trainControl('cv',number = 10),
    tuneLength = 10
  )
  model$bestTune
  coef(model$finalModel,model$bestTune$lambda)
  #数据预测
  x.test <- model.matrix(medv~., test.data)[,-1]
  #验证集分类的预测
  predictions <- model %>% predict(x.test)
  library(ROCR)
  probFull <- predict(model, x.test, type = "raw")
  predictFull <- prediction(probFull, test.data$medv)
  ##模型的ROC评估
  {#模型的数值
    value <- as.data.frame(predictFull@predictions[[1]])
    #验证集的分组
    group <- as.data.frame(test.data$medv)
```

```
    #整合数据集供ROC检验
    df <- cbind(group,value)
    library(dplyr)
    df <- tibble::rownames_to_column(df,"id")
    names(df) <- c("ID","Group","Model")
    library(multipleROC)
    df <- as.data.frame(df)
    p <- multipleROC(Group~Model,data=df)
    plot_ROC(p,
             show.points = T,
             show.eta = T,
             show.sens = T,
             show.AUC = T,
             facet = F )
  }
  ROC统计结果<- data.frame("参数"=c("cutpoint","sens","auc"),
                      "Model"=c(p[["cutpoint"]],
                                p[["sens"]],
                                p[["spec"]],
                                p[["auc"]]))
  #ROC统计结果1<-ROC统计结果
  ROC统计结果1<- merge(ROC统计结果1,ROC统计结果,by = "参数")
}
}

##模型的ROC评估
{#模型的数值
  value <- as.data.frame(predictFull@predictions[[1]])
  #验证集的分组
  group <- as.data.frame(test.data$medv)
  #整合数据集供ROC检验
  df <- cbind(group,value)
  library(dplyr)
  df <- tibble::rownames_to_column(df,"id")
  names(df) <- c("ID","Group","Model")
  library(multipleROC)
  df <- as.data.frame(df)
  p <- multipleROC(Group~Model,data=df)
  plot_ROC(p,
           show.points = T,
           show.eta = T,
           show.sens = T,
           show.AUC = T,
           facet = F )
}
ROC统计结果<- data.frame("参数"=c("cutpoint","sens","auc"),
                    "Model"=c(p[["cutpoint"]],
                              p[["sens"]],
                              p[["spec"]],
                              p[["auc"]]))
#ROC统计结果1<-ROC统计结果
ROC统计结果1<- merge(ROC统计结果1,ROC统计结果,by = "参数")

#数据整理，更改列名
{
  r<- ROC统计结果1
  rownames(r) <- r$参数
  r <- r[,-(1)]
  #列名改为数字
  colnames(r) <- 1:32
  original_cols <- colnames(r)
  colnames(r) <- paste("Model" ,"No",original_cols,sep="_")
  class(r)
  #储存结果
  if(!dir.exists("ENR prediction result")){dir.create("ENR prediction result")}
```

```
  write.csv(r,"ENR prediction result/LASSO.csv")
}

####逻辑回归（Logistic regression）####
#加载包
library(tidyverse)
library(caret)
library(glmnet)
library(openxlsx)
library(data.table)
library(mlbench)
###数据准备
#加载数据
#示例数据
#data('Boston',package='MASS')
data <- read.csv("GSE40553_exp.csv")
rownames(data) <- data$X
data <- data[,-1]
data <- t(data)
data <- as.data.frame(data)
group <- read.csv("GSE40553_group.csv")
rownames(group) <- group$geo_accession
#分组信息需要手动处理
group <- group[,-2]
group <- group[,-2]
group <- group[,-2]
group <- group[,-(2:7)]
group <- group[,-(2:4)]
group <- group[,-(3:30)]
group <- group[,-(3:24)]
#整合数据集
{
  LASSO <- cbind(group,data)
  LASSO <- LASSO[,-1]
  library(dplyr)
  group <- group %>% filter(grepl('ATB|LTBI',group))
  LASSO_1 <- LASSO %>% filter(grepl('ATB|LTBI',group))
  LASSO_1[LASSO_1=="ATB"]<-1
  LASSO_1[LASSO_1=="LTBI"]<-0
  data <- LASSO_1
  data[,c(1)] <- as.numeric(unlist(data[,c(1)]))
  Boston <- data
}
#Boston <- read.xlsx("GSEWhole for ROC.xlsx")
#数据处理
{
  #数据格式同lasso
  setnames (Boston,
            old=c ("group"),
            new=c ("medv"))
  #挑选目的3基因
  Boston<- Boston[,grepl("medv|GPR84|S100A12|S100A8",
                         colnames(Boston))]
  #按顺序排列
  target <- c("medv","GPR84","S100A12","S100A8")
  # 然后将 dataframe 按照新的列名顺序排列
  Boston <- Boston[,target]
  #分为训练集和验证集
  set.seed(123)
  trainning.sample <- Boston$medv %>% createDataPartition(p=0.8,list=FALSE)
  train.data <- Boston[trainning.sample,]
  test.data <- Boston[-trainning.sample,]
  y <- train.data$medv
}
```

```
#第一个数据集的评估
{
#第1个模型：12
{
  model <- glm(medv~GPR84+S100A12,
               data=train.data,family=binomial)
  x.test <- model.matrix(medv~GPR84+S100A12,
                         test.data%>% select(1,2,3))[,-1]
  predictions <- model %>% predict(as.data.frame(x.test))
  probFull <- predict(model,as.data.frame(x.test),type = "response")
  predictFull <- prediction(probFull, test.data$medv)
  ##模型的ROC评估
  {#模型的数值
    value <- as.data.frame(predictFull@predictions[[1]])
    #验证集的分组
    group <- as.data.frame(test.data$medv)
    #整合数据集供ROC检验
    df <- cbind(group,value)
    library(dplyr)
    df <- tibble::rownames_to_column(df,"id")
    names(df) <- c("ID","Group","Model")
    library(multipleROC)
    df <- as.data.frame(df)
    p <- multipleROC(Group~Model,data=df)
    plot_ROC(p,
             show.points = T,
             show.eta = T,
             show.sens = T,
             show.AUC = T,
             facet = F )
  }
  ROC统计结果<- data.frame("参数"=c("cutpoint","sens","auc"),
                     "Model"=c(p[["cutpoint"]],
                               p[["sens"]],
                               p[["spec"]],
                               p[["auc"]]))
  ROC统计结果1<-ROC统计结果
}
#第2个模型：13
{
  model <- glm(medv~GPR84+S100A8,
               data=train.data,family=binomial)
  x.test <- model.matrix(medv~GPR84+S100A8,
                         test.data%>% select(1,2,4))[,-1]
  predictions <- model %>% predict(as.data.frame(x.test))
  probFull <- predict(model,as.data.frame(x.test),type = "response")
  predictFull <- prediction(probFull, test.data$medv)
  ##模型的ROC评估
  {#模型的数值
    value <- as.data.frame(predictFull@predictions[[1]])
    #验证集的分组
    group <- as.data.frame(test.data$medv)
    #整合数据集供ROC检验
    df <- cbind(group,value)
    library(dplyr)
    df <- tibble::rownames_to_column(df,"id")
    names(df) <- c("ID","Group","Model")
    library(multipleROC)
    df <- as.data.frame(df)
    p <- multipleROC(Group~Model,data=df)
    plot_ROC(p,
             show.points = T,
             show.eta = T,
             show.sens = T,
             show.AUC = T,
```

```
            facet = F )
  }
  ROC统计结果<- data.frame("参数"=c("cutpoint","sens","auc"),
                      "Model"=c(p[["cutpoint"]],
                               p[["sens"]],
                               p[["spec"]],
                               p[["auc"]]))
  #ROC统计结果1<-ROC统计结果
  ROC统计结果1<- merge(ROC统计结果1,ROC统计结果,by = "参数")
}
#第3个模型：23
{
  model <- glm(medv~S100A12+S100A8,
               data=train.data,family=binomial)
  x.test <- model.matrix(medv~S100A12+S100A8,
                       test.data%>% select(1,3,4))[,-1]
  predictions <- model %>% predict(as.data.frame(x.test))
  probFull <- predict(model,as.data.frame(x.test),type = "response")
  predictFull <- prediction(probFull, test.data$medv)
  ##模型的ROC评估
  {#模型的数值
    value <- as.data.frame(predictFull@predictions[[1]])
    #验证集的分组
    group <- as.data.frame(test.data$medv)
    #整合数据集供ROC检验
    df <- cbind(group,value)
    library(dplyr)
    df <- tibble::rownames_to_column(df,"id")
    names(df) <- c("ID","Group","Model")
    library(multipleROC)
    df <- as.data.frame(df)
    p <- multipleROC(Group~Model,data=df)
    plot_ROC(p,
            show.points = T,
            show.eta = T,
            show.sens = T,
            show.AUC = T,
            facet = F )
  }
  ROC统计结果<- data.frame("参数"=c("cutpoint","sens","auc"),
                      "Model"=c(p[["cutpoint"]],
                               p[["sens"]],
                               p[["spec"]],
                               p[["auc"]]))
  #ROC统计结果1<-ROC统计结果
  ROC统计结果1<- merge(ROC统计结果1,ROC统计结果,by = "参数")
}
#第4个模型：123
{
  model <- glm(medv~., data=train.data,family=binomial)
  x.test <- model.matrix(medv ~., test.data%>% select(1:4))[,-1]
  predictions <- model %>% predict(as.data.frame(x.test))
  probFull <- predict(model,as.data.frame(x.test),type = "response")
  predictFull <- prediction(probFull, test.data$medv)
  ##模型的ROC评估
  {#模型的数值
    value <- as.data.frame(predictFull@predictions[[1]])
    #验证集的分组
    group <- as.data.frame(test.data$medv)
    #整合数据集供ROC检验
    df <- cbind(group,value)
    library(dplyr)
    df <- tibble::rownames_to_column(df,"id")
    names(df) <- c("ID","Group","Model")
    library(multipleROC)
```

```
    df <- as.data.frame(df)
    p <- multipleROC(Group~Model,data=df)
    plot_ROC(p,
             show.points = T,
             show.eta = T,
             show.sens = T,
             show.AUC = T,
             facet = F )
  }
  ROC统计结果<- data.frame("参数"=c("cutpoint","sens","auc"),
                     "Model"=c(p[["cutpoint"]],
                               p[["sens"]],
                               p[["spec"]],
                               p[["auc"]]))
  #ROC统计结果1<-ROC统计结果
  ROC统计结果1<- merge(ROC统计结果1,ROC统计结果,by = "参数")
}
}

#其他数据集的评估
{
#第1个模型：12
{
  model <- glm(medv~GPR84+S100A12,
               data=train.data,family=binomial)
  x.test <- model.matrix(medv~GPR84+S100A12,
                         test.data%>% select(1,2,3))[,-1]
  predictions <- model %>% predict(as.data.frame(x.test))
  probFull <- predict(model,as.data.frame(x.test),type = "response")
  predictFull <- prediction(probFull, test.data$medv)
  ##模型的ROC评估
  {#模型的数值
    value <- as.data.frame(predictFull@predictions[[1]])
    #验证集的分组
    group <- as.data.frame(test.data$medv)
    #整合数据集供ROC检验
    df <- cbind(group,value)
    library(dplyr)
    df <- tibble::rownames_to_column(df,"id")
    names(df) <- c("ID","Group","Model")
    library(multipleROC)
    df <- as.data.frame(df)
    p <- multipleROC(Group~Model,data=df)
    plot_ROC(p,
             show.points = T,
             show.eta = T,
             show.sens = T,
             show.AUC = T,
             facet = F )
  }
  ROC统计结果<- data.frame("参数"=c("cutpoint","sens","auc"),
                     "Model"=c(p[["cutpoint"]],
                               p[["sens"]],
                               p[["spec"]],
                               p[["auc"]]))
  #ROC统计结果1<-ROC统计结果
  ROC统计结果1<- merge(ROC统计结果1,ROC统计结果,by = "参数")
}
#第2个模型：13
{
  model <- glm(medv~GPR84+S100A8,
               data=train.data,family=binomial)
  x.test <- model.matrix(medv~GPR84+S100A8,
                         test.data%>% select(1,2,4))[,-1]
  predictions <- model %>% predict(as.data.frame(x.test))
```

```r
  probFull <- predict(model,as.data.frame(x.test),type = "response")
  predictFull <- prediction(probFull, test.data$medv)
  ##模型的ROC评估
  {#模型的数值
    value <- as.data.frame(predictFull@predictions[[1]])
    #验证集的分组
    group <- as.data.frame(test.data$medv)
    #整合数据集供ROC检验
    df <- cbind(group,value)
    library(dplyr)
    df <- tibble::rownames_to_column(df,"id")
    names(df) <- c("ID","Group","Model")
    library(multipleROC)
    df <- as.data.frame(df)
    p <- multipleROC(Group~Model,data=df)
    plot_ROC(p,
             show.points = T,
             show.eta = T,
             show.sens = T,
             show.AUC = T,
             facet = F )
  }
  ROC统计结果<- data.frame("参数"=c("cutpoint","sens","auc"),
                      "Model"=c(p[["cutpoint"]],
                                p[["sens"]],
                                p[["spec"]],
                                p[["auc"]]))
  #ROC统计结果1<-ROC统计结果
  ROC统计结果1<- merge(ROC统计结果1,ROC统计结果,by = "参数")
}
#第3个模型：23
{
  model <- glm(medv~S100A12+S100A8,
               data=train.data,family=binomial)
  x.test <- model.matrix(medv~S100A12+S100A8,
                         test.data%>% select(1,3,4))[,-1]
  predictions <- model %>% predict(as.data.frame(x.test))
  probFull <- predict(model,as.data.frame(x.test),type = "response")
  predictFull <- prediction(probFull, test.data$medv)
  ##模型的ROC评估
  {#模型的数值
    value <- as.data.frame(predictFull@predictions[[1]])
    #验证集的分组
    group <- as.data.frame(test.data$medv)
    #整合数据集供ROC检验
    df <- cbind(group,value)
    library(dplyr)
    df <- tibble::rownames_to_column(df,"id")
    names(df) <- c("ID","Group","Model")
    library(multipleROC)
    df <- as.data.frame(df)
    p <- multipleROC(Group~Model,data=df)
    plot_ROC(p,
             show.points = T,
             show.eta = T,
             show.sens = T,
             show.AUC = T,
             facet = F )
  }
  ROC统计结果<- data.frame("参数"=c("cutpoint","sens","auc"),
                      "Model"=c(p[["cutpoint"]],
                                p[["sens"]],
                                p[["spec"]],
                                p[["auc"]]))
  #ROC统计结果1<-ROC统计结果
```

```
  ROC统计结果1<- merge(ROC统计结果1,ROC统计结果,by = "参数")
}
#第4个模型：123
{
  model <- glm(medv~., data=train.data,family=binomial)
  x.test <- model.matrix(medv ~., test.data%>% select(1:4))[,-1]
  predictions <- model %>% predict(as.data.frame(x.test))
  probFull <- predict(model,as.data.frame(x.test),type = "response")
  predictFull <- prediction(probFull, test.data$medv)
  ##模型的ROC评估
  {#模型的数值
    value <- as.data.frame(predictFull@predictions[[1]])
    #验证集的分组
    group <- as.data.frame(test.data$medv)
    #整合数据集供ROC检验
    df <- cbind(group,value)
    library(dplyr)
    df <- tibble::rownames_to_column(df,"id")
    names(df) <- c("ID","Group","Model")
    library(multipleROC)
    df <- as.data.frame(df)
    p <- multipleROC(Group~Model,data=df)
    plot_ROC(p,
             show.points = T,
             show.eta = T,
             show.sens = T,
             show.AUC = T,
             facet = F )
  }
  ROC统计结果<- data.frame("参数"=c("cutpoint","sens","auc"),
                      "Model"=c(p[["cutpoint"]],
                               p[["sens"]],
                               p[["spec"]],
                               p[["auc"]]))
  #ROC统计结果1<-ROC统计结果
  ROC统计结果1<- merge(ROC统计结果1,ROC统计结果,by = "参数")
}
}

##模型的ROC评估
{#模型的数值
  value <- as.data.frame(predictFull@predictions[[1]])
  #验证集的分组
  group <- as.data.frame(test.data$medv)
  #整合数据集供ROC检验
  df <- cbind(group,value)
  library(dplyr)
  df <- tibble::rownames_to_column(df,"id")
  names(df) <- c("ID","Group","Model")
  library(multipleROC)
  df <- as.data.frame(df)
  p <- multipleROC(Group~Model,data=df)
  plot_ROC(p,
           show.points = T,
           show.eta = T,
           show.sens = T,
           show.AUC = T,
           facet = F )
}
ROC统计结果<- data.frame("参数"=c("cutpoint","sens","auc"),
                    "Model"=c(p[["cutpoint"]],
                             p[["sens"]],
                             p[["spec"]],
                             p[["auc"]]))
#ROC统计结果1<-ROC统计结果
```

```
ROC统计结果1<- merge(ROC统计结果1,ROC统计结果,by = "参数")

#数据整理，更改列名
{
  r<- ROC统计结果1
  rownames(r) <- r$参数
  r <- r[,-(1)]
  #列名改为数字
  colnames(r) <- 1:32
  original_cols <- colnames(r)
  colnames(r) <- paste("Model" ,"No",original_cols,sep="_")
  class(r)
  #储存结果
  if(!dir.exists("MLR prediction result")){dir.create("MLR prediction result")}
  write.csv(r,"MLR prediction result/MLR.csv")
}

####SVM1####
##加载包
library(tidyverse)
library(caret)
library(glmnet)
library(openxlsx)
library(data.table)
library(mlbench)
library(ROCR)
###数据准备
#加载数据
#示例数据
#data('Boston',package='MASS')
data <- read.csv("GSE40553_exp.csv")
rownames(data) <- data$X
data <- data[,-1]
data <- t(data)
data <- as.data.frame(data)
group <- read.csv("GSE40553_group.csv")
rownames(group) <- group$geo_accession
#分组信息需要手动处理
group <- group[,-2]
group <- group[,-2]
group <- group[,-2]
group <- group[,-(2:7)]
group <- group[,-(2:4)]
group <- group[,-(3:30)]
group <- group[,-(3:24)]
#整合数据集
{
  LASSO <- cbind(group,data)
  LASSO <- LASSO[,-1]
  library(dplyr)
  group <- group %>% filter(grepl('ATB|LTBI',group))
  LASSO_1 <- LASSO %>% filter(grepl('ATB|LTBI',group))
  LASSO_1[LASSO_1=="ATB"]<-1
  LASSO_1[LASSO_1=="LTBI"]<-0
  data <- LASSO_1
  data[,c(1)] <- as.numeric(unlist(data[,c(1)]))
  Boston <- data
}
#Boston <- read.xlsx("GSEWhole for ROC.xlsx")
#数据处理
{
  #数据格式同lasso
  setnames (Boston,
            old=c ("group"),
            new=c ("medv"))
```

```
  #挑选目的3基因
  Boston<- Boston[,grepl("medv|GPR84|S100A12|S100A8",
                         colnames(Boston))]
  #按顺序排列
  target <- c("medv","GPR84","S100A12","S100A8")
  # 然后将 dataframe 按照新的列名顺序排列
  Boston <- Boston[,target]
  #分为训练集和验证集
  set.seed(123)
  trainning.sample <- Boston$medv %>% createDataPartition(p=0.8,list=FALSE)
  train.data <- Boston[trainning.sample,]
  test.data <- Boston[-trainning.sample,]
  y <- train.data$medv
}

#首次的第1个模型：12
{
  model <- train(medv~GPR84+S100A12,
                 train.data%>%select(1,2,3),
                 method = 'svmLinear',
                 trControl = trainControl('cv',number = 10),
                 preProcess = c('center','scale'))
  x.test <- model.matrix(medv~GPR84+S100A12,
                         test.data%>%select(1,2,3))[,-1]
  predictions <- model %>% predict(as.data.frame(x.test))
  probFull <- predict(model,as.data.frame(x.test),type = "raw")
  predictFull <- prediction(probFull, test.data$medv)
  ##模型的ROC评估
  {#模型的数值
    value <- as.data.frame(predictFull@predictions[[1]])
    #验证集的分组
    group <- as.data.frame(test.data$medv)
    #整合数据集供ROC检验
    df <- cbind(group,value)
    library(dplyr)
    df <- tibble::rownames_to_column(df,"id")
    names(df) <- c("ID","Group","Model")
    library(multipleROC)
    df <- as.data.frame(df)
    p <- multipleROC(Group~Model,data=df)
    plot_ROC(p,
             show.points = T,
             show.eta = T,
             show.sens = T,
             show.AUC = T,
             facet = F )
  }
  ROC统计结果<- data.frame("参数"=c("cutpoint","sens","auc"),
                      "Model"=c(p[["cutpoint"]],
                                p[["sens"]],
                                p[["spec"]],
                                p[["auc"]]))
  ROC统计结果1<-ROC统计结果
}

#拟合回归模型
{
#第1个模型：12
{
  model <- train(medv~GPR84+S100A12,
                 train.data%>%select(1,2,3),
                 method = 'svmLinear',
                 trControl = trainControl('cv',number = 10),
                 preProcess = c('center','scale'))
  x.test <- model.matrix(medv~GPR84+S100A12,
```

```
                          test.data%>%select(1,2,3))[,-1]
  predictions <- model %>% predict(as.data.frame(x.test))
  probFull <- predict(model,as.data.frame(x.test),type = "raw")
  predictFull <- prediction(probFull, test.data$medv)
  ##模型的ROC评估
  {#模型的数值
    value <- as.data.frame(predictFull@predictions[[1]])
    #验证集的分组
    group <- as.data.frame(test.data$medv)
    #整合数据集供ROC检验
    df <- cbind(group,value)
    library(dplyr)
    df <- tibble::rownames_to_column(df,"id")
    names(df) <- c("ID","Group","Model")
    library(multipleROC)
    df <- as.data.frame(df)
    p <- multipleROC(Group~Model,data=df)
    plot_ROC(p,
             show.points = T,
             show.eta = T,
             show.sens = T,
             show.AUC = T,
             facet = F )
  }
  ROC统计结果<- data.frame("参数"=c("cutpoint","sens","auc"),
                        "Model"=c(p[["cutpoint"]],
                                  p[["sens"]],
                                  p[["spec"]],
                                  p[["auc"]]))
  #ROC统计结果1<-ROC统计结果
  ROC统计结果1<- merge(ROC统计结果1,ROC统计结果,by = "参数")
}
#第2个模型：13
{
  model <- train(medv~GPR84+S100A8,
                 train.data%>%select(1,2,4),
                 method = 'svmLinear',
                 trControl = trainControl('cv',number = 10),
                 preProcess = c('center','scale'))
  x.test <- model.matrix(medv~GPR84+S100A8,
                          test.data%>%select(1,2,4))[,-1]
  predictions <- model %>% predict(as.data.frame(x.test))
  probFull <- predict(model,as.data.frame(x.test),type = "raw")
  predictFull <- prediction(probFull, test.data$medv)
  ##模型的ROC评估
  {#模型的数值
    value <- as.data.frame(predictFull@predictions[[1]])
    #验证集的分组
    group <- as.data.frame(test.data$medv)
    #整合数据集供ROC检验
    df <- cbind(group,value)
    library(dplyr)
    df <- tibble::rownames_to_column(df,"id")
    names(df) <- c("ID","Group","Model")
    library(multipleROC)
    df <- as.data.frame(df)
    p <- multipleROC(Group~Model,data=df)
    plot_ROC(p,
             show.points = T,
             show.eta = T,
             show.sens = T,
             show.AUC = T,
             facet = F )
  }
  ROC统计结果<- data.frame("参数"=c("cutpoint","sens","auc"),
```

```
                    "Model"=c(p[["cutpoint"]],
                              p[["sens"]],
                              p[["spec"]],
                              p[["auc"]]))
  #ROC统计结果1<-ROC统计结果
  ROC统计结果1<- merge(ROC统计结果1,ROC统计结果,by = "参数")
}
#第3个模型：23
{
  model <- train(medv~S100A12+S100A8,
                 train.data%>%select(1,3,4),
                 method = 'svmLinear',
                 trControl = trainControl('cv',number = 10),
                 preProcess = c('center','scale'))
  x.test <- model.matrix(medv~S100A12+S100A8,
                         test.data%>%select(1,3,4))[,-1]
  predictions <- model %>% predict(as.data.frame(x.test))
  probFull <- predict(model,as.data.frame(x.test),type = "raw")
  predictFull <- prediction(probFull, test.data$medv)
  ##模型的ROC评估
  {#模型的数值
    value <- as.data.frame(predictFull@predictions[[1]])
    #验证集的分组
    group <- as.data.frame(test.data$medv)
    #整合数据集供ROC检验
    df <- cbind(group,value)
    library(dplyr)
    df <- tibble::rownames_to_column(df,"id")
    names(df) <- c("ID","Group","Model")
    library(multipleROC)
    df <- as.data.frame(df)
    p <- multipleROC(Group~Model,data=df)
    plot_ROC(p,
             show.points = T,
             show.eta = T,
             show.sens = T,
             show.AUC = T,
             facet = F )
  }
  ROC统计结果<- data.frame("参数"=c("cutpoint","sens","auc"),
                    "Model"=c(p[["cutpoint"]],
                              p[["sens"]],
                              p[["spec"]],
                              p[["auc"]]))
  #ROC统计结果1<-ROC统计结果
  ROC统计结果1<- merge(ROC统计结果1,ROC统计结果,by = "参数")
}
#第4个模型：123
{
  model <- train(medv~.,
                 train.data,
                 method = 'svmLinear',
                 trControl = trainControl('cv',number = 10),
                 preProcess = c('center','scale'))
  x.test <- model.matrix(medv~.,
                         test.data)[,-1]
  predictions <- model %>% predict(as.data.frame(x.test))
  probFull <- predict(model,as.data.frame(x.test),type = "raw")
  predictFull <- prediction(probFull, test.data$medv)
  ##模型的ROC评估
  {#模型的数值
    value <- as.data.frame(predictFull@predictions[[1]])
    #验证集的分组
    group <- as.data.frame(test.data$medv)
    #整合数据集供ROC检验
```

```
    df <- cbind(group,value)
    library(dplyr)
    df <- tibble::rownames_to_column(df,"id")
    names(df) <- c("ID","Group","Model")
    library(multipleROC)
    df <- as.data.frame(df)
    p <- multipleROC(Group~Model,data=df)
    plot_ROC(p,
             show.points = T,
             show.eta = T,
             show.sens = T,
             show.AUC = T,
             facet = F )
  }
  ROC统计结果<- data.frame("参数"=c("cutpoint","sens","auc"),
                     "Model"=c(p[["cutpoint"]],
                               p[["sens"]],
                               p[["spec"]],
                               p[["auc"]]))
  #ROC统计结果1<-ROC统计结果
  ROC统计结果1<- merge(ROC统计结果1,ROC统计结果,by = "参数")
}
}

##模型的ROC评估
{#模型的数值
  value <- as.data.frame(predictFull@predictions[[1]])
  #验证集的分组
  group <- as.data.frame(test.data$medv)
  #整合数据集供ROC检验
  df <- cbind(group,value)
  library(dplyr)
  df <- tibble::rownames_to_column(df,"id")
  names(df) <- c("ID","Group","Model")
  library(multipleROC)
  df <- as.data.frame(df)
  p <- multipleROC(Group~Model,data=df)
  plot_ROC(p,
           show.points = T,
           show.eta = T,
           show.sens = T,
           show.AUC = T,
           facet = F )
}
ROC统计结果<- data.frame("参数"=c("cutpoint","sens","auc"),
                     "Model"=c(p[["cutpoint"]],
                               p[["sens"]],
                               p[["spec"]],
                               p[["auc"]]))
#ROC统计结果1<-ROC统计结果
ROC统计结果1<- merge(ROC统计结果1,ROC统计结果,by = "参数")

#数据整理，更改列名
{
  r<- ROC统计结果1
  rownames(r) <- r$参数
  r <- r[,-(1)]
  #列名改为数字
  colnames(r) <- 1:32
  original_cols <- colnames(r)
  colnames(r) <- paste("Model" ,"No",original_cols,sep="_")
  class(r)
  #储存结果
  if(!dir.exists("SVM prediction result")){dir.create("SVM prediction result")}
  write.csv(r,"SVM prediction result/SVM.csv")
```

```
}

####NaiveBayes####
#加载包
{
library(tidyverse)
library(caret)
library(openxlsx)
library(data.table)
library(mlbench)
library(klaR)
library(tidyverse)
library(caret)
library(glmnet)
library(openxlsx)
library(data.table)
library(mlbench)
library(ROCR)
}
###数据准备
#加载数据
#示例数据
#data('Boston',package='MASS')
data <- read.csv("GSE94438_exp.csv")
rownames(data) <- data$Sample
data <- data[,-1]
data <- t(data)
data <- as.data.frame(data)
group <- read.csv("GSE40553_group.csv")
rownames(group) <- group$geo_accession
#分组信息需要手动处理
group <- group[,-2]
group <- group[,-2]
group <- group[,-2]
group <- group[,-(2:7)]
group <- group[,-(2:4)]
group <- group[,-(3:30)]
group <- group[,-(3:24)]
#整合数据集
{
  LASSO <- cbind(group,data)
  LASSO <- LASSO[,-1]
  library(dplyr)
  group <- group %>% filter(grepl('ATB|LTBI',group))
  LASSO_1 <- LASSO %>% filter(grepl('ATB|LTBI',group))
  LASSO_1[LASSO_1=="ATB"]<-1
  LASSO_1[LASSO_1=="LTBI"]<-0
  data <- LASSO_1
  data[,c(1)] <- as.numeric(unlist(data[,c(1)]))
  Boston <- data
}
#Boston <- read.xlsx("GSEWhole for ROC.xlsx")
#数据处理
{
  #数据格式同lasso
  setnames (Boston,
            old=c ("group"),
            new=c ("medv"))
  #挑选目的3基因
  Boston<- Boston[,grepl("medv|GPR84|S100A12|S100A8",
                         colnames(Boston))]
  #按顺序排列
  target <- c("medv","GPR84","S100A12","S100A8")
  # 然后将 dataframe 按照新的列名顺序排列
  Boston <- Boston[,target]
```

```
  #分为训练集和验证集
  set.seed(123)
  trainning.sample <- Boston$medv %>% createDataPartition(p=0.8,list=FALSE)
  train.data <- Boston[trainning.sample,]
  test.data <- Boston[-trainning.sample,]
  y <- train.data$medv
  #因子转换
  train.data$medv<-as.factor(train.data$medv)
}

#首次运算的第1个模型：12
{
  model <- NaiveBayes(medv~GPR84+S100A12,
                      data = train.data %>% dplyr::select(1,2,3))
  x.test <- model.matrix(medv~GPR84+S100A12,
                         test.data %>% dplyr::select(1,2,3))[,-1]
  predictions <- model %>% predict(x.test)
  probFull <- predict(model,x.test,type = "raw")
  ##模型的ROC评估
  {#模型的数值
    value <- as.data.frame(probFull[["posterior"]])
    #验证集的分组
    group <- as.data.frame(test.data$medv)
    #整合数据集供ROC检验
    df <- cbind(group,value)
    library(dplyr)
    df <- tibble::rownames_to_column(df,"id")
    names(df) <- c("ID","Group","Model")
    library(multipleROC)
    df <- as.data.frame(df)
    p <- multipleROC(Group~Model,data=df)
    plot_ROC(p,
             show.points = T,
             show.eta = T,
             show.sens = T,
             show.AUC = T,
             facet = F )
  }
  ROC统计结果<- data.frame("参数"=c("cutpoint","sens","auc"),
                       "Model"=c(p[["cutpoint"]],
                                 p[["sens"]],
                                 p[["spec"]],
                                 p[["auc"]]))
  ROC统计结果1<-ROC统计结果
}

#模型计算
{
#第1个模型：12
{
  model <- NaiveBayes(medv~GPR84+S100A12,
                      data = train.data %>% dplyr::select(1,2,3))
  x.test <- model.matrix(medv~GPR84+S100A12,
                         test.data %>% dplyr::select(1,2,3))[,-1]
  predictions <- model %>% predict(x.test)
  probFull <- predict(model,x.test,type = "raw")
  ##模型的ROC评估
  {#模型的数值
    value <- as.data.frame(probFull[["posterior"]])
    #验证集的分组
    group <- as.data.frame(test.data$medv)
    #整合数据集供ROC检验
    df <- cbind(group,value)
    library(dplyr)
    df <- tibble::rownames_to_column(df,"id")
```

```
    names(df) <- c("ID","Group","Model")
    library(multipleROC)
    df <- as.data.frame(df)
    p <- multipleROC(Group~Model,data=df)
    plot_ROC(p,
             show.points = T,
             show.eta = T,
             show.sens = T,
             show.AUC = T,
             facet = F )
  }
  ROC统计结果<- data.frame("参数"=c("cutpoint","sens","auc"),
                     "Model"=c(p[["cutpoint"]],
                               p[["sens"]],
                               p[["spec"]],
                               p[["auc"]]))
  #ROC统计结果1<-ROC统计结果
  ROC统计结果1<- merge(ROC统计结果1,ROC统计结果,by = "参数")
}
#第2个模型：13
{
  model <- NaiveBayes(medv~GPR84+S100A8,
                    data = train.data%>%dplyr::select(1,2,4))
  x.test <- model.matrix(medv~GPR84+S100A8,
                       test.data%>%dplyr::select(1,2,4))[,-1]
  predictions <- model %>% predict(x.test)
  probFull <- predict(model,x.test,type = "raw")
  ##模型的ROC评估
  {#模型的数值
    value <- as.data.frame(probFull[["posterior"]])
    #验证集的分组
    group <- as.data.frame(test.data$medv)
    #整合数据集供ROC检验
    df <- cbind(group,value)
    library(dplyr)
    df <- tibble::rownames_to_column(df,"id")
    names(df) <- c("ID","Group","Model")
    library(multipleROC)
    df <- as.data.frame(df)
    p <- multipleROC(Group~Model,data=df)
    plot_ROC(p,
             show.points = T,
             show.eta = T,
             show.sens = T,
             show.AUC = T,
             facet = F )
  }
  ROC统计结果<- data.frame("参数"=c("cutpoint","sens","auc"),
                     "Model"=c(p[["cutpoint"]],
                               p[["sens"]],
                               p[["spec"]],
                               p[["auc"]]))
  #ROC统计结果1<-ROC统计结果
  ROC统计结果1<- merge(ROC统计结果1,ROC统计结果,by = "参数")
}
#第3个模型：23
{
  model <- NaiveBayes(medv~S100A12+S100A8,
                    data = train.data%>%dplyr::select(1,3,4))
  x.test <- model.matrix(medv~S100A12+S100A8,
                       test.data%>%dplyr::select(1,3,4))[,-1]
  predictions <- model %>% predict(x.test)
  probFull <- predict(model,x.test,type = "raw")
  ##模型的ROC评估
  {#模型的数值
```

```
    value <- as.data.frame(probFull[["posterior"]])
    #验证集的分组
    group <- as.data.frame(test.data$medv)
    #整合数据集供ROC检验
    df <- cbind(group,value)
    library(dplyr)
    df <- tibble::rownames_to_column(df,"id")
    names(df) <- c("ID","Group","Model")
    library(multipleROC)
    df <- as.data.frame(df)
    p <- multipleROC(Group~Model,data=df)
    plot_ROC(p,
             show.points = T,
             show.eta = T,
             show.sens = T,
             show.AUC = T,
             facet = F )
  }
  ROC统计结果<- data.frame("参数"=c("cutpoint","sens","auc"),
                     "Model"=c(p[["cutpoint"]],
                               p[["sens"]],
                               p[["spec"]],
                               p[["auc"]]))
  #ROC统计结果1<-ROC统计结果
  ROC统计结果1<- merge(ROC统计结果1,ROC统计结果,by = "参数")
}
#第4个模型：123
{
  model <- NaiveBayes(medv~., data = train.data)
  x.test <- model.matrix(medv~.,
                         test.data)[,-1]
  predictions <- model %>% predict(x.test)
  probFull <- predict(model,x.test,type = "raw")
  ##模型的ROC评估
  {#模型的数值
    value <- as.data.frame(probFull[["posterior"]])
    #验证集的分组
    group <- as.data.frame(test.data$medv)
    #整合数据集供ROC检验
    df <- cbind(group,value)
    library(dplyr)
    df <- tibble::rownames_to_column(df,"id")
    names(df) <- c("ID","Group","Model")
    library(multipleROC)
    df <- as.data.frame(df)
    p <- multipleROC(Group~Model,data=df)
    plot_ROC(p,
             show.points = T,
             show.eta = T,
             show.sens = T,
             show.AUC = T,
             facet = F )
  }
  ROC统计结果<- data.frame("参数"=c("cutpoint","sens","auc"),
                     "Model"=c(p[["cutpoint"]],
                               p[["sens"]],
                               p[["spec"]],
                               p[["auc"]]))
  #ROC统计结果1<-ROC统计结果
  ROC统计结果1<- merge(ROC统计结果1,ROC统计结果,by = "参数")
}
}

##模型的ROC评估
{#模型的数值
```

```
  value <- as.data.frame(probFull[["posterior"]])
  #验证集的分组
  group <- as.data.frame(test.data$medv)
  #整合数据集供ROC检验
  df <- cbind(group,value)
  library(dplyr)
  df <- tibble::rownames_to_column(df,"id")
  names(df) <- c("ID","Group","Model")
  library(multipleROC)
  df <- as.data.frame(df)
  p <- multipleROC(Group~Model,data=df)
  plot_ROC(p,
           show.points = T,
           show.eta = T,
           show.sens = T,
           show.AUC = T,
           facet = F )
}
ROC统计结果<- data.frame("参数"=c("cutpoint","sens","auc"),
                    "Model"=c(p[["cutpoint"]],
                              p[["sens"]],
                              p[["spec"]],
                              p[["auc"]]))
#ROC统计结果1<-ROC统计结果
ROC统计结果1<- merge(ROC统计结果1,ROC统计结果,by = "参数")

#数据整理，更改列名
{
  r<- ROC统计结果1
  rownames(r) <- r$参数
  r <- r[,-(1)]
  #列名改为数字
  colnames(r) <- 1:32
  original_cols <- colnames(r)
  colnames(r) <- paste("Model" ,"No",original_cols,sep="_")
  class(r)
  #储存结果
  if(!dir.exists("NB prediction result")){dir.create("NB prediction result")}
  write.csv(r,"NB prediction result/NB.csv")
}

####多个数据集log2逆转换强行合并####
library(glmnet)
library(readxl)
library(plyr)
library(caret)
library(corrplot)
library(ggplot2)
library(Hmisc)
library(openxlsx)
library(Cairo)
data <- read.xlsx("GSEWhole.xlsx")
pdata <- read.xlsx("GSEWhole_group.xlsx")
#设置参考水平
group_list <- ifelse(str_detect(pdata$title,
                                "ATB"), "ATB","LTBI")
#因子型
group_list = factor(group_list,
                    levels = c("LTBI","ATB"))

####xCell 进行免疫浸润分析####
getwd()
#根据64种免疫细胞和基质细胞的基因表达数据进行细胞类型的分析
##安装包
install.packages("ggplot2")
```

```
install.packages("tidyverse")
install.packages("reshape2")
install.packages("corrplot")
install.packages("devtools")
library(devtools)
devtools::install_github('dviraran/xCell')

##载入需要的R包
library(tidyverse)
library(ggplot2)
library(reshape2)
library(corrplot)
library(xCell)
library(openxlsx)
library(data.table)
library(mlbench)
library(ROCR)
#读取数据
exp <- read.csv("GSE37250_WGCNA_Down_analysis.csv")
group <- read.csv("GSE37250_Group.csv")
Group <- group$group

#数据格式修改
rownames(exp) <- exp$X
exp <- exp[,-1]
#转置
exp <- t(exp)
exp <- as.matrix(exp)
##数据分析
#xCell计算免疫细胞矩阵
#??xCellAnalysis
xcell<-xCellAnalysis(exp,
                     signatures = xCell.data$signatures,
                     genes = xCell.data$genes,
                     spill = NULL,
                     rnaseq = TRUE,
                     file.name = NULL,
                     scale = TRUE,
                     alpha = 0.5,
                     save.raw = FALSE,
                     parallel.sz = 4,
                     parallel.type = "SOCK",
                     cell.types.use = NULL)
#保存xCell计算结果
write.table(xcell,file="xCell.txt",row.names=T,header=T,sep="\t",quote=F)
#目标基因集
genelist<-
c("IGF2","CRABP1","PCP4","DEFB1","HOXB8","MMP7","LYPD2","AOC1","RAB4B","PFN2","PIGV","RAD23A")
#提取基因集的表达矩阵
goal_exp<-filter(exp,rownames(exp) %in%genelist)
#合并目标基因集表达矩阵和免疫细胞矩阵
combine<-rbind(goal_exp,xcell)
#计算相关系数
comcor<-cor(t(combine))
#计算显著性差异
comp<-cor.mtest(comcor,conf.level=0.95)
pval<-comp$p
#获取目标基因相关性矩阵
goalcor<-select(as.data.frame(comcor),genelist)%>%rownames_to_column(var="celltype")
goalcor<-filter(goalcor,!(celltype %in% genelist))
##长宽数据转换
goalcor<-melt(goalcor,id.vars="celltype")
colnames(goalcor)<-c("celltype","Gene","correlation")
#获取目标基因集pvalue矩阵
pval<-select(as.data.frame(pval),genelist)%>%rownames_to_column(var="celltype")
```

```
pval<-filter(pval,!(celltype %in% genelist))
#长宽数据转换
pval<-melt(pval,id.vars="celltype")
colnames(pval)<-c("celltype","gene","pvalue")
#将pvalue和correlation两个文件合并
final<-left_join(goalcor,pval,by=c("celltype"="celltype","Gene"="gene"))

##绘图
#添加一列,来判断pvalue值范围
final$sign<-case_when(final$pvalue<0.05 &final$pvalue>0.01 ~"*",
                      final$pvalue<0.01 &final$pvalue>0.001 ~"**",
                      final$pvalue<0.001 ~"***",
                      final$pvalue>0.05 ~"")
ggplot(data=final,aes(x=Gene,y=celltype))+
  geom_tile(aes(fill=correlation),colour="white",size=1)+
  scale_fill_gradient2(low="#2b8cbe",mid="white",high="#e41a1c")+
  geom_text(aes(label=sign),colour="black")+
  theme_minimal()+
  theme(axis.text.x=element_text(angle=45,hjust=1,size=12),
        axis.text.y=element_text(size=12),
        axis.title.x=element_blank(),
        axis.title.y=element_blank(),
        axis.ticks.x=element_blank(),
        axis.ticks.y=element_blank()) +
  guides(fill=guide_legend(title="* p<0.05\n\n** p<0.01\n\n*** p<0.001\n\ncorrelation"))
ggsave("correlation.pdf",width=12,height=13)

####绘制分组箱式图####
##载入需要的R包
library(tidyverse)
library(ggplot2)
library(reshape2)
library(corrplot)
library(openxlsx)
library(data.table)
library(mlbench)
library(ROCR)
library(dplyr)
library(tidyr)
library(tibble)
#install.packages("ggpubr")
library(ggpubr)
#读取数据
data <- read.xlsx("Model-whole.xlsx")
group <- read.xlsx("model group.xlsx")
data <- cbind(data,group)
#筛选并命名为新名称
#data1 <- data %>% filter(时间<=ymd('20220716'))
#data1 <- data1 %>% mutate(类型='上半月')

#将类型转换为因子，保持画图的时候顺序不变
data$Model_category=factor(data$Model_category)

#这一部分筛选出每个类型中最大的值，为添加P值定位而准备的
tibble:::as_tibble.data.frame(data)
tibble:::repaired_names(data)
location <- data %>% group_by(Model_category) %>% slice_max(auc)
location$x <- seq(1,7,by=1)
head(location,3)

# 绘制小提琴图
ggplot(data,aes(Ridge,auc,fill=Gene))+
  geom_violin(scale = "width",alpha=0.8,width=0.5,size=0.8)+ #画小提琴图
  scale_fill_manual(values = c("#F7903D","4D85BD"))+          #分组添加颜色
  stat_compare_means(aes(group=Gene),                         #按分组进行统计检验
```

```
                     method = "t.test",
                     paired = F,                             #非配对t检验
                     symnum.args = list(cutpoint=c(0,0.001,0.01,0.05,1),
                                        symbols=c("***","**","*","ns")),
                     label = "p.signif",
                     label.y = location$Gene+0.02,      #添加显著性符号的位置
                     size=4.5)+                               #显著性符号的大小
  geom_segment(data=location,                                 #在显著性符号下面添加一条短线
               aes(x=x,y=Gene,
                   xend=x+0.2,yend=auc),
               size=1)+
  xlab("")+                                                   #X标签
  ylab("AUC")+                                           #y标签
  theme_bw()+
  theme(panel.grid.major=element_blank(),
        panel.grid.minor=element_blank(),
        panel.border=element_rect(size=1.2),                  #给边框加粗
        axis.text.x = element_text(angle=60,size=10,vjust = 1,hjust =1,color = "black"),
        axis.text.y = element_text(size =10),
        legend.position = c(0.9,0.85))

####八种免疫浸润分析####
#https://mp.weixin.qq.com/s/JqO7rVBMGGmOXRA8w8nDSg
if (!requireNamespace("IOBR", quietly = TRUE))
  devtools::install_github("IOBR/IOBR")

#加载包
library(IOBR)
library(openxlsx)

#读取数据
expr_coad <- read.csv("GSE37250_WGCNA_Down_analysis.csv")

# MCPcounter
im_mcpcounter <- deconvo_tme(eset = expr_coad,
                             method = "mcpcounter"
)
##
## >>> Running MCP-counter

# EPIC
im_epic <- deconvo_tme(eset = expr_coad,
                       method = "epic",
                       arrays = F
)

# xCell
im_xcell <- deconvo_tme(eset = expr_coad,
                        method = "xcell",
                        arrays = F
)

# CIBERSORT
im_cibersort <- deconvo_tme(eset = expr_coad,
                            method = "cibersort",
                            arrays = F,
                            perm = 1000
)

# IPS
im_ips <- deconvo_tme(eset = expr_coad,
                      method = "ips",
                      plot = F
)
```

```
# quanTIseq
im_quantiseq <- deconvo_tme(eset = expr_coad,
                            method = "quantiseq",
                            scale_mrna = T
)

# ESTIMATE
im_estimate <- deconvo_tme(eset = expr_coad,
                           method = "estimate"
)

# TIMER
im_timer <- deconvo_tme(eset = expr_coad
                        ,method = "timer"
                        ,group_list = rep("coad",dim(expr_coad)[2])
)

#整合
tme_combine <- im_mcpcounter %>%
  inner_join(im_epic, by="ID") %>%
  inner_join(im_xcell, by="ID") %>%
  inner_join(im_cibersort, by="ID") %>%
  inner_join(im_ips, by= "ID") %>%
  inner_join(im_quantiseq, by="ID") %>%
  inner_join(im_estimate, by= "ID") %>%
  inner_join(im_timer, by= "ID")


####单基因免疫浸润分析CIBERSORT####
#library(devtools)
#if(!require(CIBERSORT))devtools::install_github("Moonerss/CIBERSORT")
#1
getwd()
setwd("D:\\临床\\结核生信\\结核生信分析20230425\\5.三基因signature的数据查明")
#加载包
{
  library(CIBERSORT)
  library(ggplot2)
  library(pheatmap)
  library(ggpubr)
  library(reshape2)
  library(tidyverse)
  library(glmnet)
  library(readxl)
  library(plyr)
  library(caret)
  library(corrplot)
  library(Hmisc)
  library(openxlsx)
  library(data.table)
}
getwd()
###数据读入
#Group <- read.csv("GSE37250_Group.csv")
data1 <- read.csv("GSE41055.csv")

#浸润分析及结果表格
{
  rownames(data1) <- data1$X
  data1 <- data1[,-1]
  #看一下数据表达的情况
  boxplot(data1,outline=F,notch=F,las=2)

  #如果均数不一致需标准化
  library(limma)
```

```
  data1=normalizeBetweenArrays(data1)
  boxplot(data1,outline=F,notch=F,las=2)

  #加载LM22肿免疫细胞marker
  #perm表示是否采用随机排列法，越小越不严谨，可以尝试1000
  data(LM22)
  result <- cibersort(sig_matrix = LM22,
                      mixture_file = data1,perm = 1000,QN=T)#时间稍微有点长
  result <- as.data.frame(result)
  #因为短杠无法识别所以需要重新命名
  setnames(result,"P-value","Pvalue")
  #筛选P小于0.05的
  #result = result %>% filter(Pvalue<0.05)
  #pvalue越小越可信
  #correlation原表达矩阵乘以细胞占比后的数据矩阵与原表达矩阵的相关性
  #RMSE均方根误差，越小效果越好
  #保存csv文件结果
  write.csv(result,"TME.csv")
}

#后续想直接自动实现Wilcon test
{
  ###处理
  res <- result
  #插入一列名为sample
  names(res)[1]<-"sample"
  #将列名设为第一列
  res[,1] <- rownames(res)

  dataExpr <- res
  dataExpr <-dataExpr[,-1]
  dataExpr <-dataExpr[,-(22:24)]
  #0变为NA
  dataExpr[dataExpr==0] <- NA
  table(is.na(dataExpr))
  index=which(is.na(dataExpr))
  res1=data.imputation(dataExpr,fun="median")
  res2=data.imputation(dataExpr,fun="mean")
  table(complete.cases(res1))
  table(complete.cases(res2))
  #直接删掉整行全是缺失值的
  exp1 <- na.omit(res1)
  exp2 <- na.omit(res2)
  #检查缺失值
  table(is.na(exp1))
  table(is.na(exp2))
  #矩阵转换为数据框
  dataExpr <- as.data.frame(exp2)
  sample <- as.data.frame(rownames(dataExpr))
  dataExpr <- cbind(dataExpr,sample)
  names(dataExpr)[names(dataExpr) == 'rownames(dataExpr)'] <- 'sample'
  #将分组信息补入
  data <- merge(dataExpr,Group)
  #更改标题中的 为_
  #提取细胞种类
  Colnames <- as.data.frame(colnames(data))
  gsub(" ","_",Colnames)
  colnames(data) <- c(Colnames)



  rownames(data) <- data$sample
  data <- data[,-1]

  #显著性检验
```

```
  library(stats)
  B_cells_naive <- wilcox.test(B_cells_naive ~ group,data=data)


  wilcox.test(B_cells_memory ~ group,data=data, var.equal = TRUE)




  Plasma_cells <- wilcox.test(Plasma_cells ~ group,data=res)
  T_cells_CD8 <- wilcox.test(T_cells_CD8 ~ group,data=res)
  T_cells_CD4_naive <- wilcox.test(T_cells_CD4_naive ~ group,data=res)
  T_cells_CD4_memory_resting <- wilcox.test(T_cells_CD4_memory_resting ~ group,data=res)
  T_cells_CD4_memory_activated <- wilcox.test(T_cells_CD4_memory_activated ~ group,data=res)
  T_cells_follicular_helper <- wilcox.test(T_cells_follicular_helper ~ group,data=res)
  B_cells_memory <- wilcox.test(B_cells_memory ~ group,data=res)
  B_cells_memory <- wilcox.test(B_cells_memory ~ group,data=res)
  B_cells_memory <- wilcox.test(B_cells_memory ~ group,data=res)
  B_cells_memory <- wilcox.test(B_cells_memory ~ group,data=res)
  B_cells_naive <- wilcox.test(B_cells_naive ~ group,data=res)
  B_cells_memory <- wilcox.test(B_cells_memory ~ group,data=res)
  B_cells_memory <- wilcox.test(B_cells_memory ~ group,data=res)
  B_cells_memory <- wilcox.test(B_cells_memory ~ group,data=res)
  B_cells_memory <- wilcox.test(B_cells_memory ~ group,data=res)
  B_cells_memory <- wilcox.test(B_cells_memory ~ group,data=res)
  B_cells_naive <- wilcox.test(B_cells_naive ~ group,data=res)
  B_cells_memory <- wilcox.test(B_cells_memory ~ group,data=res)
  B_cells_memory <- wilcox.test(B_cells_memory ~ group,data=res)
  B_cells_memory <- wilcox.test(B_cells_memory ~ group,data=res)
  B_cells_memory <- wilcox.test(B_cells_memory ~ group,data=res)
  B_cells_memory <- wilcox.test(B_cells_memory ~ group,data=res)
  P_Monocytes <- wilcox.test(Monocytes ~ group,data=res)


  #按组别计算统计信息（中位数和四分位数范围（IQR））。可以使用dplyr软件包。
  install.packages("dplyr")
  library(dplyr)
  group_by(data, group) %>%
    summarise(
      count = n(),
      mean = mean(weight, na.rm = TRUE),
      sd = sd(weight, na.rm = TRUE)
    )
  #看一下数据表达的情况
  boxplot(data,outline=F,notch=F,las=2)
}

####mantel test相关性热图####
#https://mp.weixin.qq.com/s/6SQ90t5O4b1H-NviYHf6ag
#相关包的安装与载入
{
  library(linkET)
  library(ggplot2)
  library(ggtext)
  library(dplyr)
  library(vegan)
  #配色主题包载入：
  library(cols4all)
}

#组合网络热图绘制
#读入非热图数据：
varespec <- read.xlsx("箱式图 for ROC.xlsx",sheet = "GSE37250")
varespec <- read.xlsx("箱式图 for ROC.xlsx",sheet = "GSE39939")
varespec <- read.xlsx("箱式图 for ROC.xlsx",sheet = "GSE39940")
```

```
varespec <- read.xlsx("箱式图 for ROC.xlsx",sheet = "GSE101705")
varespec <- read.xlsx("箱式图 for ROC.xlsx",sheet = "GSE112104")
varespec <- read.xlsx("箱式图 for ROC.xlsx",sheet = "GSE19491")
varespec <- read.xlsx("箱式图 for ROC.xlsx",sheet = "GSE25534")
varespec <- read.xlsx("箱式图 for ROC.xlsx",sheet = "GSE28623")
varespec <- read.xlsx("箱式图 for ROC.xlsx",sheet = "GSE41055")

#读入热图数据：
otu.tab <- read.csv("TME.csv")

#数据处理
{
  rownames(varespec) <- varespec$sample
  varespec <- varespec[,-(1:2)]
  varespec <- varespec[,-3]
  varechem <- as.data.frame(otu.tab)
  rownames(varechem) <- varechem$X
  varechem <- varechem[,-(24:26)]
  varechem <- varechem[,-1]
}

#绘图及计算
{
  #计算环境因子相关性系数：
  cor2 <- correlate(varechem)
  corr2 <- cor2 %>% as_md_tbl()
  write.csv(corr2, file = "pearson_correlate(env&env).csv", row.names = TRUE)

  #mantel test:
  mantel <- mantel_test(varespec, varechem,
                        mantel_fun = 'mantel', #支持4种："mantel"使用
vegan::mantel()；"mantel.randtest"使用ade4::mantel.randtest()；"mantel.rtest"使用
ade4::mantel.rtest()；"mantel.partial"使用vegan::mantel.partial()
                        spec_select = list(GBP5 = 1,
                                           LHFPL2 = 2))
}
#保存数据及绘图
{
  write.csv(mantel, file = "mantel_result(bio&env).csv", row.names = TRUE)
  #对mantel的r和P值重新赋值（设置绘图标签）：
  mantel2 <- mantel %>%
    mutate(r = cut(r, breaks = c(-Inf, 0.25, 0.5, Inf),
                   labels = c("<0.25", "0.25-0.5", ">=0.5")),
           p = cut(p, breaks = c(-Inf, 0.001, 0.01, 0.05, Inf),
                   labels = c("<0.001", "0.001-0.01", "0.01-0.05", ">= 0.05")))
  head(mantel2)

  #首先，绘制相关性热图(和上文相同)：
  p4 <- qcorrplot(cor2,
                  grid_col = "grey50",
                  grid_size = 0.2,
                  type = "upper",
                  diag = FALSE) +
    geom_square() +
    scale_fill_gradientn(colours = c4a('rd_bu',30),
                         limits = c(-1, 1))
  p4

  #添加显著性标签：
  p5 <- p4 +
    geom_mark(size = 4,
              only_mark = T,
              sig_level = c(0.05, 0.01, 0.001),
              sig_thres = 0.05,
              colour = 'white')
```

```
  p5
  #在相关性热图上添加mantel连线：
  p6 <- p5 +
    geom_couple(data = mantel2,
                aes(colour = p, size = r),
                curvature = nice_curvature())
  p6
  #继续美化连线：
  p7 <- p6 +
    scale_size_manual(values = c(0.5, 1.0, 1.5, 2.0)) + #连线粗细
    scale_colour_manual(values = c4a('brewer.set2',4)) + #连线配色
    #修改图例：
    guides(size = guide_legend(title = "Mantel's r",
                               override.aes = list(colour = "grey35"),
                               order = 2),
           colour = guide_legend(title = "Mantel's p",
                                 override.aes = list(size = 3),
                                 order = 1),
           fill = guide_colorbar(title = "Pearson's r", order = 3))
  p7
}


####Upset####
# 加载R包，没有安装请先安装  install.packages("包名")
library(UpSetR) #Upset图（upset 包，适用样本数 2-7）
library(VennDiagram)
library(openxlsx)
# 读取数据文件
upset_dat <- read.xlsx("汇总.xlsx")# 这里读取了网络上的demo数据，将此处换成你自己电脑里的文件
upset_list <- list(upset_dat[,1],
                   upset_dat[,2],
                   upset_dat[,3],
                   upset_dat[,4],
                   upset_dat[,5],
                   upset_dat[,6],
                   upset_dat[,7],
                   upset_dat[,8],
                   upset_dat[,9],
                   upset_dat[,10],
                   upset_dat[,11],
                   upset_dat[,12],
                   upset_dat[,13],
                   upset_dat[,14],
                   upset_dat[,15],
                   upset_dat[,16])   # 制作Upset图搜所需要的列表文件
names(upset_list) <- colnames(upset_dat[1:16])    # 把列名赋值给列表的key值

#作图
upset(fromList(upset_list),  # fromList一个函数，用于将列表转换为与UpSetR兼容的数据形式。
      nsets = 100,     # 绘制的最大集合个数
      nintersects = 40, #绘制的最大交集个数，NA则全部绘制
      order.by = "freq", # 矩阵中的交点是如何排列的。 "freq"根据交集个数排序，"degree"根据
      keep.order = F, # 保持设置与使用sets参数输入的顺序一致。默认值是FALSE，它根据集合的大小排序。
      mb.ratio = c(0.6,0.4),   # 左侧和上方条形图的比例关系
      text.scale = 2 # 文字标签的大小
      )
# 更多参数 ?upset查看
# 查看交集详情,并导出结果
inter <- get.venn.partitions(upset_list)
for (i in 1:nrow(inter)) inter[i,'values'] <- paste(inter[[i,'..values..']], collapse = '|')
inter <- subset(inter, select = -..values.. )
inter <- subset(inter, select = -..set.. )
write.table(inter, "result.csv", row.names = FALSE, sep = ',', quote = FALSE)
```

```
####统计多个ROC结果####
getwd()
library(multipleROC)
library(openxlsx)

data <- read.csv("GSE40553_exp.csv")
rownames(data) <- data$X
data <- data[,-1]
data <- t(data)
data <- as.data.frame(data)
group <- read.csv("GSE40553_group.csv")
rownames(group) <- group$geo_accession
#分组信息需要手动处理
group <- group[,-2]
group <- group[,-2]
group <- group[,-2]
group <- group[,-(2:7)]
group <- group[,-(3:30)]
#整合数据集
{
LASSO <- cbind(group,data)
LASSO <- LASSO[,-1]
library(dplyr)
group <- group %>% filter(grepl('ATB|LTBI',group))
LASSO_1 <- LASSO %>% filter(grepl('ATB|LTBI',group))
LASSO_1[LASSO_1=="ATB"]<-1
LASSO_1[LASSO_1=="LTBI"]<-0
data <- LASSO_1
data[,c(1)] <- as.numeric(unlist(data[,c(1)]))
}
#多个ROC曲线分页绘制
{
p1 <-  multipleROC(group~ ANXA3 ,data=data)
p2 <-  multipleROC(group~ GPR84 ,data=data)
p3 <-  multipleROC(group~ MCEMP1 ,data=data)
p4 <-  multipleROC(group~ MMP9 ,data=data)
p5 <-  multipleROC(group~ S100A12 ,data=data)
p6 <-  multipleROC(group~ S100A8 ,data=data)
p7 <-  multipleROC(group~ GBP1 ,data=data)
p8 <-  multipleROC(group~ GBP5 ,data=data)
p9 <-  multipleROC(group~ IFI27 ,data=data)
p10 <-  multipleROC(group~ IFIT3 ,data=data)
p11 <-  multipleROC(group~ PLSCR1 ,data=data)
p12 <-  multipleROC(group~ RSAD2 ,data=data)
p13 <-  multipleROC(group~ AIM2 ,data=data)
p14 <-  multipleROC(group~ CXCR5 ,data=data)
p15 <-  multipleROC(group~ NAIP ,data=data)
p16 <-  multipleROC(group~ NLRC4 ,data=data)
p17 <-  multipleROC(group~ BPI ,data=data)
p18 <-  multipleROC(group~ DEFA4 ,data=data)
p19 <-  multipleROC(group~ ELANE ,data=data)
p20 <-  multipleROC(group~ C1QA ,data=data)
p21 <-  multipleROC(group~ FCGBP ,data=data)
p22 <-  multipleROC(group~ SERPING1 ,data=data)
p23 <-  multipleROC(group~ FCAR ,data=data)
p24 <-  multipleROC(group~ FCGR1A ,data=data)
p25 <-  multipleROC(group~ FCGR1B ,data=data)
p26 <-  multipleROC(group~ LCN2 ,data=data)
p27 <-  multipleROC(group~ VNN1 ,data=data)
p28 <-  multipleROC(group~ COL17A1 ,data=data)
p29 <-  multipleROC(group~ PLOD2 ,data=data)
p30 <-  multipleROC(group~ CYP1B1 ,data=data)
p31 <-  multipleROC(group~ MGST1 ,data=data)
}
#统计结果
```

```
ROC统计结果<- data.frame("参数"=c("cutpoint","sens","auc"),
                    " p1 "=c( p1[["cutpoint"]], p1[["auc"]], p1[["sens"]], p1[["spec"]]),
                    " p2 "=c( p2[["cutpoint"]], p2[["auc"]], p2[["sens"]], p2[["spec"]]),
                    " p3 "=c( p3[["cutpoint"]], p3[["auc"]], p3[["sens"]], p3[["spec"]]),
                    " p4 "=c( p4[["cutpoint"]], p4[["auc"]], p4[["sens"]], p4[["spec"]]),
                    " p5 "=c( p5[["cutpoint"]], p5[["auc"]], p5[["sens"]], p5[["spec"]]),
                    " p6 "=c( p6[["cutpoint"]], p6[["auc"]], p6[["sens"]], p6[["spec"]]),
                    " p7 "=c( p7[["cutpoint"]], p7[["auc"]], p7[["sens"]], p7[["spec"]]),
                    " p8 "=c( p8[["cutpoint"]], p8[["auc"]], p8[["sens"]], p8[["spec"]]),
                    " p9 "=c( p9[["cutpoint"]], p9[["auc"]], p9[["sens"]], p9[["spec"]]),
                    " p10 "=c( p10[["cutpoint"]], p10[["auc"]], p10[["sens"]], p10[["spec"]]),
                    " p11 "=c( p11[["cutpoint"]], p11[["auc"]], p11[["sens"]], p11[["spec"]]),
                    " p12 "=c( p12[["cutpoint"]], p12[["auc"]], p12[["sens"]], p12[["spec"]]),
                    " p13 "=c( p13[["cutpoint"]], p13[["auc"]], p13[["sens"]], p13[["spec"]]),
                    " p14 "=c( p14[["cutpoint"]], p14[["auc"]], p14[["sens"]], p14[["spec"]]),
                    " p15 "=c( p15[["cutpoint"]], p15[["auc"]], p15[["sens"]], p15[["spec"]]),
                    " p16 "=c( p16[["cutpoint"]], p16[["auc"]], p16[["sens"]], p16[["spec"]]),
                    " p17 "=c( p17[["cutpoint"]], p17[["auc"]], p17[["sens"]], p17[["spec"]]),
                    " p18 "=c( p18[["cutpoint"]], p18[["auc"]], p18[["sens"]], p18[["spec"]]),
                    " p19 "=c( p19[["cutpoint"]], p19[["auc"]], p19[["sens"]], p19[["spec"]]),
                    " p20 "=c( p20[["cutpoint"]], p20[["auc"]], p20[["sens"]], p20[["spec"]]),
                    " p21 "=c( p21[["cutpoint"]], p21[["auc"]], p21[["sens"]], p21[["spec"]]),
                    " p22 "=c( p22[["cutpoint"]], p22[["auc"]], p22[["sens"]], p22[["spec"]]),
                    " p23 "=c( p23[["cutpoint"]], p23[["auc"]], p23[["sens"]], p23[["spec"]]),
                    " p24 "=c( p24[["cutpoint"]], p24[["auc"]], p24[["sens"]], p24[["spec"]]),
                    #" p25 "=c( p25[["cutpoint"]], p25[["auc"]], p25[["sens"]], p25[["spec"]]),
                    " p26 "=c( p26[["cutpoint"]], p26[["auc"]], p26[["sens"]], p26[["spec"]]),
                    " p27 "=c( p27[["cutpoint"]], p27[["auc"]], p27[["sens"]], p27[["spec"]]),
                    " p28 "=c( p28[["cutpoint"]], p28[["auc"]], p28[["sens"]], p28[["spec"]]),
                    " p29 "=c( p29[["cutpoint"]], p29[["auc"]], p29[["sens"]], p29[["spec"]]),
                    " p30 "=c( p30[["cutpoint"]], p30[["auc"]], p30[["sens"]], p30[["spec"]]),
                    " p31 "=c( p31[["cutpoint"]], p31[["auc"]], p31[["sens"]], p31[["spec"]]))
write.xlsx(ROC统计结果,"ROC统计结果.xlsx")

####t检验####
library(reshape2)
#读入文件，合并分组信息，数据重排
alpha <- read.csv('GSE94438.csv')
rownames(alpha) <- alpha$Sample
alpha <- alpha[,-1]
group  <- read.csv('GSE94438_group.csv')
rownames(group) <- group$X
group <- group[,-(3:10)]
alpha <- cbind(group,alpha)
alpha <- alpha[,-1]
library(dplyr)
library(ggpubr)
#group <- group %>% filter(grepl('ATB|LTBI',group))
alpha <- alpha %>% filter(grepl('ATB|LTBI',group))
alpha$group<-as.factor(alpha$group)
##配色板
"#E16A86" "#909800" "#00AD9A" "#9183E6" "#ffb3ffff" "#0080ffff"
##统计检验S100A12,S100A8
compare_means(S100A12~group, alpha, method = "wilcox.test",
              paired = FALSE,)
p <- ggboxplot(alpha, x = "group", y = "S100A12",
               color = "supp", palette = "jco",
               add = "jitter")
#  Add p-value
p + stat_compare_means()
# Change method
p + stat_compare_means(method = "wilcox.test")

####新的技能记得添加####
####新的技能记得添加####
```

####新的技能记得添加####
####新的技能记得添加####
####新的技能记得添加####
